# Profiling dynamic decision-makers

**DOI:** 10.1371/journal.pone.0266366

**Published:** 2022-04-14

**Authors:** Konrad Grabiszewski, Alex Horenstein

**Affiliations:** 1 Mohammed Bin Salman College, King Abdullah Economic City, Saudi Arabia; 2 Department of Economics, University of Miami, Coral Gables, FL, United States of America; Hanken School of Economics, FINLAND

## Abstract

From CEOs confronting competition to children playing board games, our professional and personal lives are full of dynamic decisions. Naturally, while playing the role of a decision-maker, people differ. To comprehend and analyze how they differ, first it is necessary to construct a profiling method that classifies dynamic decision-makers. Developing such a method is the main objective of our article. We equate dynamic decision-making with backward inducting. We rely on response times to construct the profiles. Our method has both descriptive power and predictive power: a subject’s profile resembles her reasoning process and forecasts the likelihood of her correctly backward inducting. To test the proposed profiling method, we use data generated by 22 different finite dynamic scenarios from the mobile app *Blues and Reds*. Our sample consists of 35,826 observations from 6,463 subjects located in 141 countries. We construct the profiles of our subjects, and, in a variety of exercises supported by an array of robustness checks, we successfully establish the predictive power of our profiling method.

*You ask what is the use of classification, arrangement, systemization? I answer you: order and simplification are the first steps toward the mastery of a subject—the actual enemy is the unknown*.

Thomas Mann

## Introduction

From Plato’s typology of political regimes to Linnaean taxonomy of organisms to Mendeleev’s periodic table, science has been built on the classification, arrangement, and systemization of knowledge for advancing our comprehension of natural and social phenomena. Social sciences also rely on categorization as a fundamental scientific activity: classification of markets (economics), typology of generic strategies (management), taxonomy of consumer behavior (marketing)—to name but a few from the plethora of readily available examples.

In the same spirit, this article develops a method of *profiling* dynamic decision-makers. The focus is on decisions in dynamic settings because of how omnipresent and important such settings are in our lives. In particular, we analyze scenarios in which backward inductions yields the optimal outcome for a decision-maker.

What type of dynamic decision-maker is she/he? What type of dynamic decision-maker am I? Addressing these questions calls for a system of classifying people in their roles as dynamic decision-makers. With this motivation in mind, we propose a profiling method that satisfies two natural requirements ([1]): descriptive power and predictive power. A person’s profile portrays her reasoning process (descriptive power) and forecasts her behavior (predictive power).

To collect the experimental data, we use a novel methodology that takes advantage of the omnipresence of mobile technology and opens attractive, yet relatively unexplored, opportunities to conduct global large-scale experiments. More precisely, we employed a team of developers to create *Blues and Reds*, a mobile app available for free on iOS and Android devices since August 2017. The app’s objective is to run experiments—due to their nature called “mobile experiments”—and collect data. Everyone with access to Google Play or the App Store can become a subject in a mobile experiment, and there are billions of people with such access.

Data in this article comes from 22 dynamic tasks in *Blues and Reds*, each played by a human subject against Artificial Intelligence (AI). Subjects either win or lose; there are no ties. Importantly, tasks that subjects face are designed in a way to clearly separate those who correctly backward induct (winners) from those who fail at backward induction (losers). Each task is a multi-round decision-making problem. The number of rounds varies from 3 to 6, and subjects choose at odd rounds while AI moves at even rounds.

Upon installing the app, a user of a mobile device becomes a subject in our experiment and goes through the mandatory tutorial that serves a role of experimental instructions. *Blues and Reds* collects the following data for each subject and each task: (i) performance record (win or loss) and (ii) response times (*RT*) measured in seconds at every round. We use response time data to construct the subjects’ profiles describing their dynamic reasoning. We use winning/losing records to test how well these profiles predict whether subjects behave consistently with backward induction.

To describe dynamic reasoning, we exploit the fact that multi-round tasks are inherently associated with a time allocation problem: while solving a task, every time it is their turn to make a choice, subjects reason what action to choose which, eventually, leads to a distribution of total thinking time across all the rounds at which they make decisions. Naturally, we are confined within the standard “as if” approach. More precisely, we do not expect people to consciously go through a process of deciding how many seconds to spend on thinking at each round. Rather, the subjects behave *as if* making such a decision. In other words, the observed distribution of thinking time is an outcome of subject’s reasoning process. While we do not know what and how precisely a subject thinks, however, we observe subject’s response times—evidence and description of the said reasoning. To capture the complexity of a person’s dynamic reasoning, the proposed profiling method constructs a two-dimensional vector.

The first dimension captures how savvy a subject is or, more precisely, how closely her dynamic reasoning resembles the thinking process required to successfully backward induct. We recognize that when it comes to backward inducting, all reasoning time should be allocated to the first round. Longer response times at later rounds indicate a suboptimality of the initially made decision. With that in mind, we compute a subject’s relative response time at round 1 as $RRT1=\frac{RT1}{TT}$ with *RT*1 being the response time at the first round and *TT* the total response time (sum of round-based *RT*s). For a savvy subject, *RRT*1 is higher in comparison to a naive subject.

The second dimension depicts how fast of a thinker a subject is. This is captured by the total response time *TT*. Following the literature, we consider *TT* as a measure of cognitive effort a subjects exerts in solving a task ([1–6]). Faster thinkers have lower *TT* compared to slower subjects.

Next, we advance the view that a lexicographic ranking is a natural order of profiles. Therefore, Ann with profile (*RRT*1_*A*_, *TT*_*A*_) is ranked higher than Bob with profile (*RRT*1_*B*_, *TT*_*B*_) if she is either savvier than him (*RRT*1_*A*_ > *RRT*1_*B*_) or, assuming they are equally savvy, faster (*TT*_*A*_ < *TT*_*B*_).

Our key empirical hypothesis is that profiles have predictive power; that is, a subject with a higher profile is more likely to behave in accordance with the backward induction algorithm. To test our hypothesis, we conduct a series of empirical exercises accompanied by several robustness checks. All the evidence validates the proposed profiling as a prediction tool.

Including the current article, data from *Blues and Reds* has been used to pursue different projects in experimental economics. In [7], we test game-form recognition. We design the same interactive problem in two different formats. One of them is more complex and requires the subjects to correctly recognize the game they play. We find that, in general, people struggle with game-form recognition which also proves to be more challenging than backward induction.

In [8], we develop an empirical measure of tree complexity. To that end, we test various metrics: average response time at the first round, average total response time, and the percentage of subjects who fail at backward induction. We find that the first two statistics work best to rank trees in terms of their complexity.

From the articles based on data from *Blues and Reds*, the closest is [9] where the main goal is to understand the relationship between *RRT*1 and *TT*. We find this relationship to be non-monotonic: for low values of *RRT*1, *TT* is negatively correlated with *RRT*1 but this correlation becomes positive when *RRT*1 is high enough.

In addition, in [9], we also investigate the power of *RRT*1 in predicting a subject’s success at backward induction. First, we treat *RRT*1 as an unconditional metric. Dividing the sample into *RRT*1-terciles shows that *RRT*1 is positively correlated with a subject’s correctly backward inducting. Second, we consider *RRT*1 conditional on *TT*. Now, we split the sample into *TT*-quintiles, and then, each quintile into *RRT*1-terciles. Conditional on *TT*, it is again shown that *RRT*1 is a good predictor of a subject’s backward inducting. These results are also confirmed using logit regression.

In this article, we go further and deeper with the analysis of *RRT*1 and *TT*. Rather than focusing on unconditional or conditional *RRT*1, we consider a pair (*RRT*1, *TT*) as a predictor. This allows us to distribute decision-makers in the two-dimensional typology with savvy/naive on one axis and fast/slow on another. Importantly, we treat *TT* as conditional on *RRT*1 because of the lexicographic ranking we advance. This article sheds new light on the usefulness of metrics built on subjects’ response times in the context of predicting their behavior.

In the literature, we find numerous examples of profiling subjects and establishing a connection between profiles and behavior (e.g., [10–15]). In general, these studies profile subjects using some form of cognitive test; the Raven Progressive Matrices ([16]) and Cognitive Reflection Test ([17]) being the most popular choices. This is where our paper distinguishes itself. Instead of measuring general cognitive skills—that is, skills related to reasoning in a plethora of contexts—we narrow down our focus only on skills that are required to make dynamic choices. Here, we follow the logic of designing task-specific tests to measure task-specific skills. For instance, to identify subject’s mathematical (or linguistic, scientific, etc.) abilities, we conduct an exam composed of mathematical (linguistic, scientific, etc.) exercises rather than a general aptitude test.

Since the second dimension of the proposed profiling relates to the speed of thinking, the closest articles related to this paper are [1, 18], and the concept of fast/slow thinking in [19]. However, the unique feature of our article is its emphases on dynamic tasks and a two-, rather than one-, dimensional structure of the proposed profiling.

Methodologically, this article belongs to the experimental literature that relies on measuring response times. In economics, this literature started with [20] and, since then, has grown exponentially (see [3, 4], and [5] for literature reviews). In particular, [21, 22] also rely on response times to predict behavior—the former in the context of individual choices and the latter in global games. However, both studies differ from our article in terms of the setting (we focus on dynamic tasks) and goal (we not only predict behavior but also develop a profiling method).

Due to our data-collection method, it is important to mention innovative methodologies like newspaper-based experiments (e.g., [23]) and online experiments (e.g., Ariel Rubinstein’s https://arielrubinstein.org/gt/, [24–26]). One of the many advantages of mobile experiments is the ease of engaging large groups of people as subjects, which is made even more effective and efficient by advertising tools like Google AdWords.

The remainder of this article proceeds as follows. Section “Experimental design” provides a detailed description of *Blues and Reds* as an experiment. Links to download *Blues and Reds* from Google Play and the App Store are on the website www.bluesandreds.com. Section “Profiles: describing reasoning” presents the construction of profiles. Section “Profiles: predicting behavior” establishes the predictive power of our profiling method. Section “Conclusions” concludes. Several robustness checks for the empirical exercises and additional empirical results are provided in Appendixes A and B in S1 Appendix. Appendix C in S1 Appendix provides the screenshot from the mandatory tutorial (experimental instructions). Finally, Appendix D in S1 Appendix contains the screenshots of all the 22 tasks that generated data for this article.

## Experimental design

### *Blues and Reds* as an experiment

*Blues and Reds* consists of dynamic tasks in which a human subject plays against Artificial Intelligence (AI) that was programmed to implement the backward induction algorithm. This article uses data from 22 tasks played after the completion of the mandatory tutorial.

**Fig 1:**
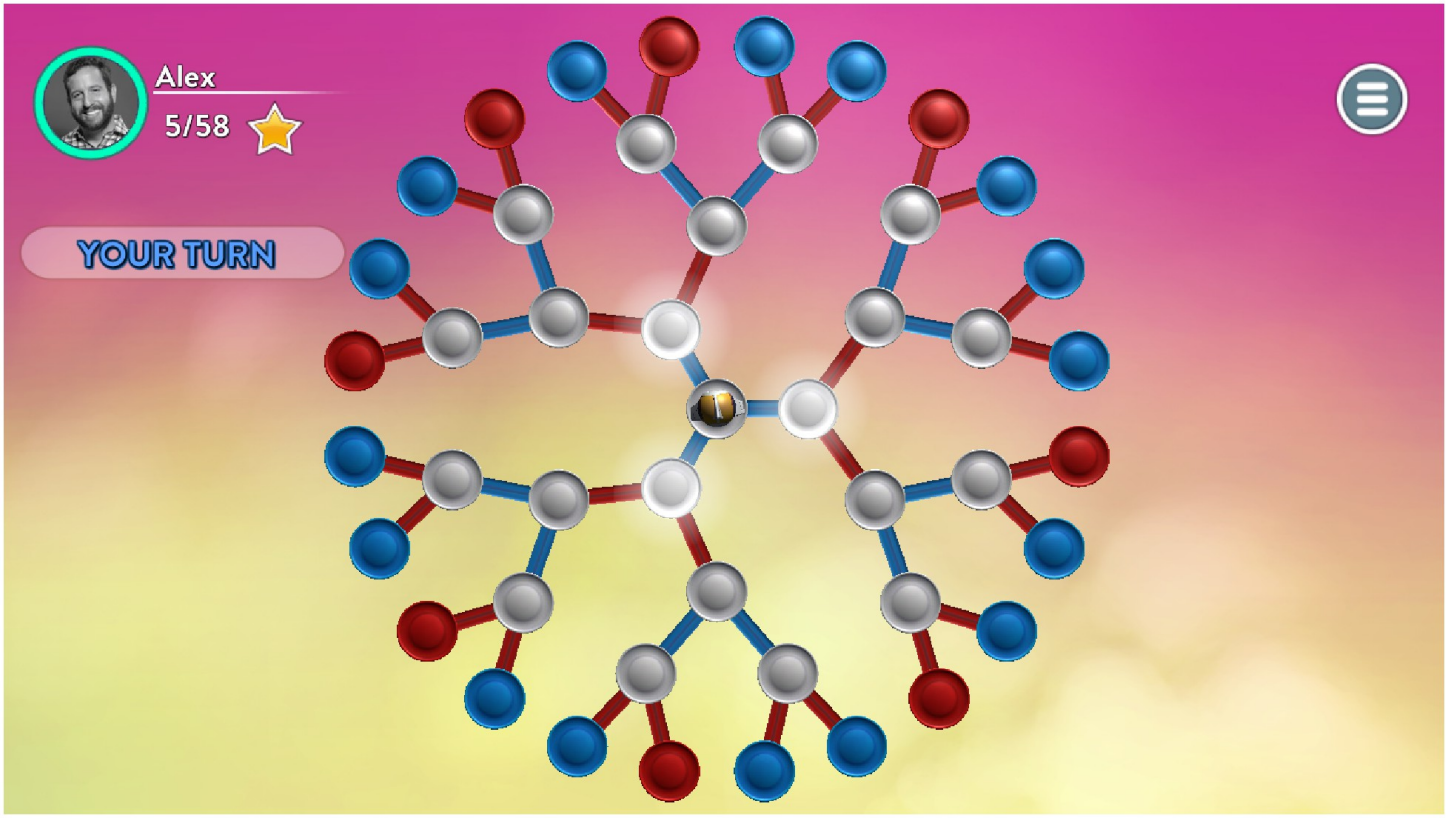
An example of a task from *Blues and Reds*.

Each of the 22 tasks resembles a tree with perfect and complete information. Fig 1 depicts an example of a task in *Blues and Reds*; Appendix D in S1 Appendix includes the screenshots of all the 22 tasks.

A task starts with the subject choosing which blue bridge the RoboToken (golden sphere) crosses. Then AI selects a red bridge for the RoboToken and the process repeats. The subject chooses at odd rounds, and AI at even rounds. If the RoboToken ends at a blue node, the subject wins; otherwise, the subject loses.

It is possible for the subject to win each task. However, this requires following the path that is the same as the unique winning path selected by the backward induction algorithm. Deviating from that path results in the subject losing as AI was designed to exploit the subject’s mistakes. Subjects are instructed that AI plays against them. There is no payoff uncertainty ([27]) as subjects learn the payoff implications of blue and red nodes and that they solve problems with zero-sum payoff structure. (See Appendix C in S1 Appendix for the screenshots of the mandatory tutorial that serves the role of experimental instructions.) In the next section, we argue that a subject’s winning is indicative of her correctly backward inducting.

Task have a symmetrical structure: the number of actions at each node of a given round is the same. A 3-round task is denoted as *N*_1_.*N*_2_.*N*_3_ where *N*_*i*_ is the number of actions at the ith round. A 4-round task is labeled as *N*_1_.*N*_2_.*N*_3_.*N*_4_, and so on. Fig 1 depicts the 3.2.2.2 task. The first column in Table 1 in Section “Data description” includes the list of all tasks from the dataset. The sequence in which the tasks appear to the subjects is randomized for each subject.

**Table 1:** Summary statistics.

Task	*N*	%*Win*	*RRT*1				*TT*			
			Mean	Sth. Dev.	Min	Max	Mean	Sth. Dev.	Min	Max
2.2.2	1,638	94%	0.71	0.11	0.13	0.93	19.05	7.17	5	50
2.2.3	1,729	94%	0.74	0.10	0.06	0.93	21.07	7.22	6	49
2.3.2	1,630	92%	0.71	0.11	0.10	0.92	21.04	7.86	8	50
2.3.3	1,637	93%	0.78	0.11	0.13	0.96	22.98	9.82	8	62
3.2.2	1,666	91%	0.75	0.11	0.15	0.94	21.03	7.76	7	49
3.3.2	1,647	90%	0.77	0.12	0.12	0.96	23.18	9.56	8	61
3.2.3	1,628	91%	0.77	0.11	0.01	0.96	21.94	8.44	8	56
3.3.3	1,638	90%	0.80	0.12	0.18	0.96	25.64	9.63	5	58
4.2.2	1,717	89%	0.77	0.12	0.11	0.95	22.68	9.79	6	62
2.2.2.2	1,660	67%	0.72	0.19	0.12	0.96	30.10	14.60	6	85
2.2.2.3	1,610	79%	0.78	0.15	0.19	0.98	33.24	17.01	6	93
2.2.3.2	1,674	77%	0.77	0.16	0.12	0.98	34.55	16.91	9	90
2.3.2.2	1,606	56%	0.73	0.20	0.12	0.98	35.98	18.44	6	101
3.2.2.2	1,575	70%	0.79	0.16	0.14	0.98	38.44	20.65	5	118
2.2.2.4	1,602	83%	0.79	0.13	0.18	0.96	32.57	15.96	7	87
2.2.4.2	1,673	73%	0.77	0.17	0.07	0.97	40.44	20.88	7	112
2.4.2.2	1,641	81%	0.78	0.14	0.18	0.96	35.84	17.94	9	96
4.2.2.2	1,614	70%	0.79	0.16	0.14	0.98	38.63	21.69	8	121
2.2.2.2.2	1,545	72%	0.69	0.19	0.06	0.95	59.43	32.15	10	184
3.2.2.2.2	1,550	67%	0.68	0.22	0.04	0.97	66.26	42.55	12	235
4.2.2.2.2	1,566	48%	0.67	0.23	0.02	0.98	95.08	78.10	10	534
2.2.2.2.2.2	1,580	47%	0.61	0.21	0.08	0.97	81.55	60.14	11	328

*Notes*. *N* denotes the number of subjects who played a given task. %*Win* is the percentage of subjects who backward inducted. For *RRT*1 and *TT*, this table provides the mean, standard deviation, and minimal and maximal values.

To become a subject in the experiment, it is necessary to install *Blues and Reds* on a mobile device (smartphone, tablet) from either Google Play or the App Store. To promote the app and find subjects, we relied on AdWords and media exposure.

Except for the tutorial, subjects can play each of the 22 tasks only once (no matter whether they win or lose). This is to incentivize subjects to reason about the choices they make. Once a task is finished with a subject’s winning or losing, the next task becomes available. Subjects do not choose a task they play, this is determined by a random sequence of tasks assigned to them.

While *Blues and Reds* offers no financial rewards, subjects collect in-app awards for correctly solving the tasks. These non-monetary incentives in tandem with the satisfaction of winning, or discontent in losing, substitute for cash incentives ([1, 28–30]). This is especially true in our experiment as subjects self-select to install the app and, consequently, have already revealed interest in solving the types of problems contained in *Blues and Reds*.

### Winning and backward inducting

Each of the 22 tasks from *Blues and Reds* that generated data for this article was designed in a way to test for the ability to backward induct. A subject who correctly implements the backward induction algorithm is guaranteed a win in each task. As mentioned in the Introduction, we interpret the results within the “as if” methodology implying that we do not expect that people actually know the backward induction algorithm and literally apply it. Instead, what we hypothesize is that their behavior is consistent with what backward induction advances. In other words, backward induction correctly models choices that people make.

At the same time, a subject who does not reason in a way consistent with backward induction, or does so but erroneously, automatically loses the task. This statement requires further elaboration since a subject might only seemingly violate backward induction—a well-known problem of significant concern in the presence of social preferences or belief in the opponent’s irrationality.

First, there is a problem of social preferences. As an example, take the centipede game ([31]). Two players alternate in deciding whether to stop or go. If Ann chooses to stop, then she gains more (in terms of monetary gains) compared to what she would gain if Bob stops at the next round but less if the opponent chooses to go. Backward induction predicts that, under standard assumptions, the first player stops the game at the first node. Since the first experiment using the centipede game ([32]), the literature has repeatedly rejected this prediction.

A typical explanation is the notion of altruism. Other explanations include mistakes ([33]), payoff uncertainty ([27]), lack of (common) knowledge of rationality ([34]), and non-standard preferences ([35]. (See [36] for the most recent survey of experiments based on the centipede game.) As [32] indicate, “if it is believed that there is some likelihood that each player may be an altruist, then it can pay a selfish player to try to mimic the behavior of an altruist in an attempt to develop a reputation for passing.” In other words, the centipede game does not test backward induction. Rather, it tests the joint hypothesis of altruism *and* backward induction.

Consequently, in the centipede game, if Ann does not stop, it is not crystal clear what the right explanation of her behavior should be. If altruism does not enter her utility, then not stopping is an indication of her violating backward induction. However, it also is possible she is a champion of backward induction who is driven by altruism; in this case, it would be erroneous to conclude that not stopping is an indicator of Ann’s inability to backward induct.

Second, there is a problem of belief that the opponents are not fully rational ([14, 15, 34, 37, 38]). In the centipede game, if Ann assigns the probability high enough to Bob being irrational, then she will not stop expecting Bob not to stop either so she can stop at a later stage and gain more. Therefore, it would be a mistake to consider Ann’s choice as a violation of backward induction.

While phenomena like social preferences and players’ irrationality are of interest and importance—after all, we never make decisions in a lab-like clean environment—they also create noise that, as discussed, make it difficult if not impossible to test for backward induction. Consequently, a subject’s choices need not reflect whether she correctly backward inducts. Without such knowledge, it is impossible to determine how much of subject’s behavior is driven by her backward-inducting skills and how much is to be contributed to other factors.

Removing noise when testing for backward-inducting skills is of critical importance. To that end *Blues and Reds* consists only of winner-takes-all tasks. As [39] observe, behavior in tasks with such a payoff structure “does not depend on social preferences or beliefs about the rationality of one’s opponent. This allows for a purer measure of players’ ability to recognize and implement backward induction strategies.” However, even in zero-sum games it is possible for social preferences to be present; for instance, a parent loses on purpose in a chess or tennis match played against their own child. To err on the side of caution, *Blues and Reds* was designed as a human vs AI rather than a human vs human experiment. As [40] argue, playing against the computer “turns off social preferences (and beliefs that other players express social preferences) by having human subjects bargain with robot players who play subgame perfectly and maximize their own earnings, and believe the humans will too.”

**Fig 2:**
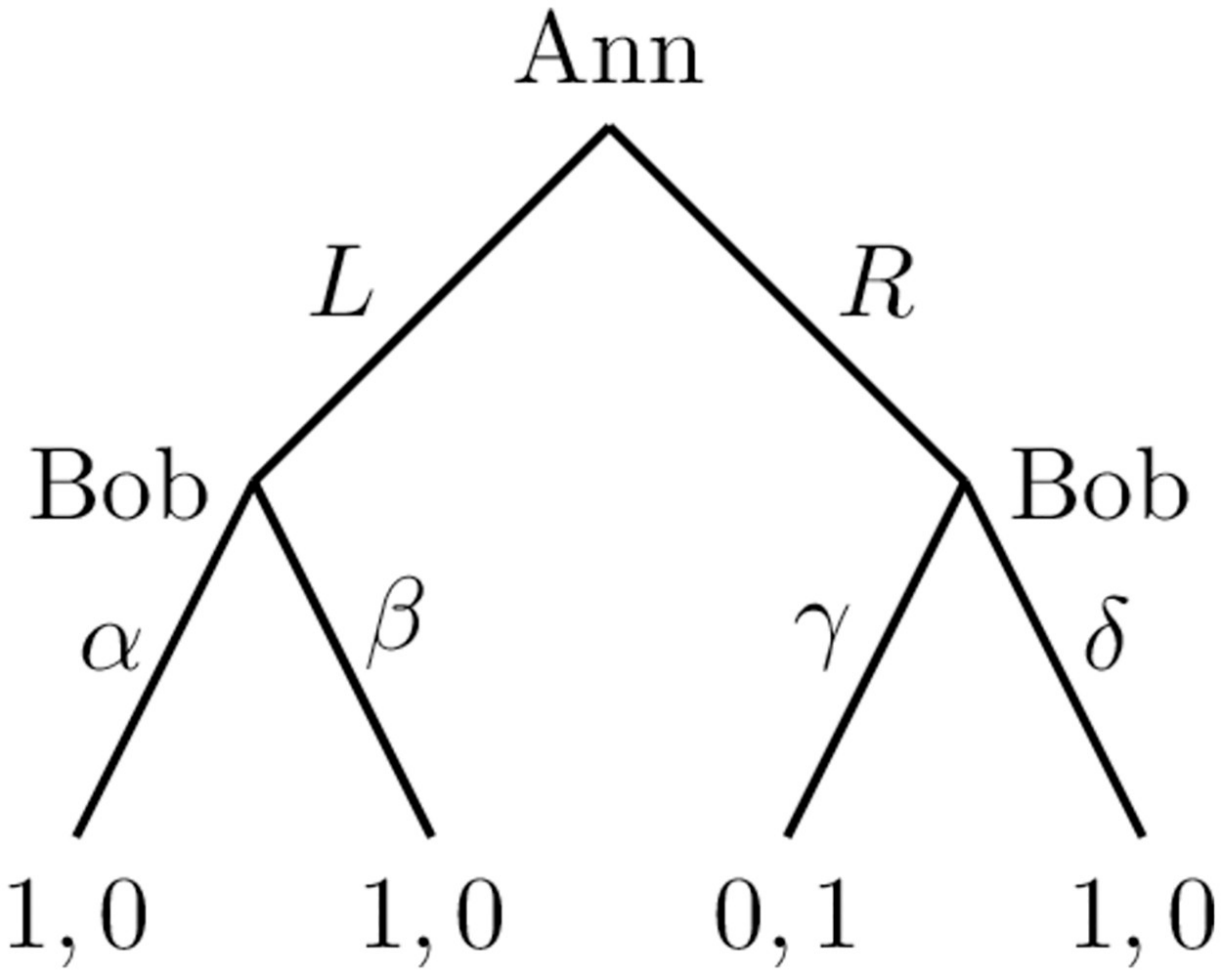
Zero-sum payoffs and belief in opponent’s rationality.

To elaborate on how the zero-sum payoffs structure allows us to disregard subjects’ belief in AI’s rationality (or lack of it), consider a simple tree depicted in Fig 2 with payoffs in the alphabetical order. In the context of our experiment, Ann plays the role of the subject while Bob is AI.

Backward induction indicates that Ann must choose *L* which guarantees her utility 1. Suppose, however, that she assigns probability *p* to Bob being irrational. More precisely, Bob will choose *δ* with probability *p* if Ann opts for *R*. However, even if the probability *p* is strictly non-zero, it is suboptimal for Ann to go with *R*. This is because *L* yields utility 1 while the expected utility from *R* is *p*. In other words, for backward-inducting Ann, *L* strictly dominates *R*.

To sum, subjects in *Blues and Reds* who do not reason in accordance with correctly implemented backward induction lose automatically. Those who do make their choices as prescribed by backward induction win with certainty.

### Data description

Data was collected between August 15, 2017 and February 6, 2018. For each task and each subject, *Blues and Reds* records the following data:

1. Whether a subject wins or loses.
2. For each round, response time (*RT*) spent on selecting an action. *RT*s are measured in seconds. Subjects act at odd rounds. Consequently, in 3- and 4-round tasks, we record *RT*1 and *RT*3—response time at round 1 and 3, respectively, and in 5- and 6-rounds tasks, we additionally record *RT*5.

From the data, the following three variables are constructed:

1. *TT*. This is a subject’s total response time in a task; i.e., the sum of round-based *RT*s.
2. *RRT*1. This is a subject’s relative time spent at the first round defined as $RRT1=\frac{RT1}{TT}$, where *RT*1 is a subject’s response time at the first round of a task.
3. *Win*. This variable takes the value of 1 if a subject wins and is otherwise 0.

A conservative data-cleaning procedure was applied, and observations with *TT* above the 95th percentile within each task were removed from the sample. The final data consists of 35,826 observations generated from 6,463 subjects located in 141 countries.

Table 1 presents the number of subjects, percentage of subjects who won (i.e., behave in accordance with backward induction), and summary statistics for *RRT*1 and *TT* for each task in the experiment.

## Profiles: Describing reasoning

### Fictional example

The objective of this section is to reason about reasoning in dynamic tasks within the context of backward induction and response times. We start with four fictional subjects who played the task 3.2.2.2 (Fig 1) and whose response times and total times are presented in Table 2. In *Blues and Reds*, subjects only choose at odd rounds; hence, the data consists only of *RT*1 and *RT*3.

**Table 2:** Response times of four fictional subjects in the task 3.2.2.2.

Subject	*RT*1	*RT*3	*TT*
Ann	15	5	20
Bob	30	10	40
Chris	8	12	20
David	16	24	40

Given the data in Table 2, the following challenges are of interest: (1) designing the subjects’ profiles that depict their dynamic reasoning processes, (2) proposing a ranking of profiles that orders subjects from the least to the most likely to behave in accordance with backward induction, and (3) testing whether that ranking indeed predicts behavior consistent with backward induction.

### Two-dimensional profile

Solving dynamic tasks is a cognitive challenge that requires spending time on reasoning. In a multi-round task, thinking takes place every times an action is to be selected. Recall that in our experiment subjects select actions at odd rounds. Hence, in 3- and 4-round tasks, they choose actions twice. In 5- and 6-rounds tasks, they make three choices.

Consequently, subjects face a time allocation problem inherent to dynamic tasks. As already mentioned in the Introduction, we interpret data in the context of decision-makers *as if* solving this time-allocation problem. As in a static decision problem, they decide how much reasoning time to allocate to the whole problem (*TT*). In addition, they also distribute that total time across all the rounds they select actions at (round-based *RT*s). This problem generates two variables of particular interest.

First, we look at total time *TT* that captures cognitive effort the subjects exert ([1–6]). We say that fast subjects are those with low *TT* and slow subjects are characterized by high *TT*. In terms of their thinking speed, in Table 2, Ann and Chris are fast, while Bob and David are slow.

Second, we compute the time allocated to thinking at the first round (*RT*1) as a fraction of the total reasoning time (*TT*), that is, the relative time a subject spends thinking before selecting an action in the first round (*RRT*1). When it comes to backward inducting, reasoning at the first round is crucial for selecting an optimal choice. This is especially relevant in *Blues and Reds* as making a mistake in any round results in a loss.

If a correct decision is made in round 1, then, during the following rounds, a subject only spends time on physically picking the right actions but no longer has to re-think what to choose. In this case, we observe a high value of *RRT*1. Time spent in later rounds indicates a possible flaw in the subject’s reasoning (e.g., making a mistake in the first round). Here, we record a low value of RRT1.

Since high *RRT*1 is consistent with correctly following the backward induction algorithm, subjects with higher *RRT*1 are called savvy while those with lower *RRT*1 are labeled as naive. In Table 2, Ann and Bob are equally savvy (*RRT*1 = 0.75) and savvier than Chris and David who are equally naive (*RRT*1 = 0.4).

To summarize, a profile is a two-dimensional vector (*RRT*1, *TT*) with *RRT*1 and *TT* capturing how savvy/naive and fast/slow, respectively, a subject is.

### Lexicographic ranking

Once the profiles have been constructed, the next challenge lies in ranking them. After all, the goal is to empirically verify that higher-ranked profiles are more likely to behave in line with backward induction. However, this test demands a definition of what it means when one profile is ranked higher than another.

There is not much to discuss regarding ranking methods for scalar profiles. However, for two-dimensional profiles like (*RRT*1, *TT*), ranking is not a trivial task as there are several ways to rank vectors. We rely on the fact that solving dynamic problems is a cognitive task. If a person does not understand how to find the optimal choice, additional time will not help them with the task. Hence, how people allocate their thinking time (*RRT*1) is more important than how much total time they spend thinking (*TT*). Therefore, a lexicographic ranking is the natural solution.

Consequently, a savvy profile (larger *RRT*1) is higher than a naive profile (smaller *RRT*1), no matter how fast/slow both are. However, the second dimension (*TT*) becomes useful for ranking profiles who are equally savvy (the same *RRT*1). Subjects who are more proficient or experienced with making dynamic decisions reason faster (a lower *TT*) as thinking is cognitively costly and spending less time on reasoning is preferable. For instance, an expert and her student are equally savvy as they share the same understanding of how to apply the backward induction algorithm. However, the expert is faster and requires less time to implement the algorithm as she has more experience solving dynamic problems, a result that has been empirically confirmed in the vast body of literature on expertise in chess (e.g., [41–44]). Consequently, assuming that the two profiles are equally savvy, the faster one is a higher profile.

To summarize, Ann with (*RRT*1_*A*_, *TT*_*A*_) is said to have a higher profile than Bob with (*RRT*1_*B*_, *TT*_*B*_) if one of the following holds:

1. Ann is savvier than Bob; i.e., *RRT*1_*A*_ > *RRT*1_*B*_.
2. Ann and Bob are equally savvy but she is faster; i.e., if *RRT*1_*A*_ = *RRT*1_*B*_, then *TT*_*A*_ < *TT*_*B*_.

Ann is the highest profile in Table 2, followed by Bob, then Chris, with David as the lowest profile. The main predictive claim of this article is that a higher profile is more likely to correctly backward induct.

We provide further empirical evidence about the importance of using a lexicographic ranking in Appendix B in S1 Appendix. Using regression analysis, we show that *TT* has predictive power about the probability of a subject correctly backward inducting only after we control by *RRT*1.

## Profiles: Predicting behavior

In this section, we empirically validate the predictive power of our profiling method. The various exercises that we conduct have the same goal: to verify whether higher profiles are more likely to behave in accordance with backward induction.

In Section “Testing predictive power: 22 replications,” we consider each dynamic task as a separate experiment. In every task, we replicate the same test to determine whether the profiling method is able to differentiate subjects according to their likelihood to correctly backward induct. In Section “Predictive power of a profile constructed from a single game,” we study whether a profile constructed from the data in a given task predicts the behavior in subsequent tasks. We also test for how long (or, for how many tasks after it was constructed) a profile maintains its predictive power. Finally, in Section “Predictive power of a profile constructed from a group of consecutive games,” we study whether constructing the profiles using multiple tasks instead of a single task improves the profiles’ predictive power.

In each exercise of this section, we partition the subjects into six profiles. First, the subjects are divided into terciles according to their *RRT*1. Second, for each *RRT*1-tercile, the subjects are further split into upper-*TT* and lower-*TT* halves with the median *TT* as a threshold value. Therefore, we use the 3-*RRT*1 by 2-*TT* division to partition subjects into six profiles.

Profile 1 corresponds to the lowest *RRT*1-tercile and upper *TT*-half—this is the most naive and the slowest profile. Profile 2 corresponds to the lowest *RRT*1-tercile and lower *TT*-half. The profile keeps increasing until we reach Profile 6, which corresponds to the subjects in the highest *RRT*1-tercile and lower *TT*-half—this is the savviest and the fastest profile.

**Fig 3:**
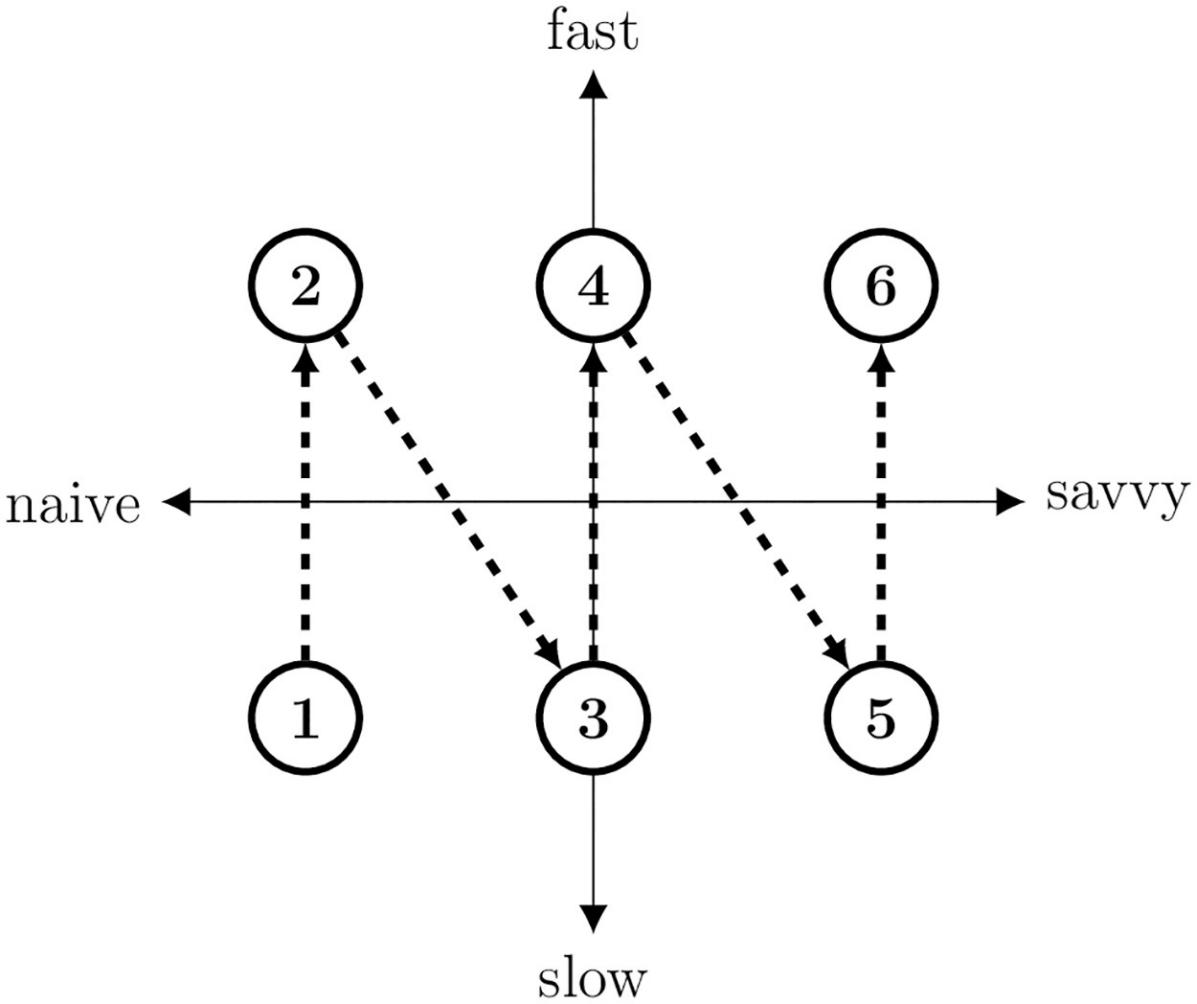
Graphical representation of the main hypothesis. *Notes*. Each dots represents a two-dimensional profile, from the lowest Profile 1 to the highest Profile 6. Probability of subject behaving consistently with backward induction increases with the direction of dashed arrows.

Fig 3 graphically summarizes the main hypothesis tested in this section. A two-dimensional space captures decision-makers’ savviness and speed of reasoning. Each dot represents a profile, from Profile 1 (the lowest) to Profile 6 (the highest). Probability of a profile correctly backward inducing increases with the direction of dashed arrows.

Appendix A in S1 Appendix contains a series of robustness checks prepared for each exercise presented in this section. First, we control for attrition by restricting the data to subjects who completed all 22 tasks analyzed in this article. Second, using the whole sample, we consider alternative partitioning of subjects into profiles: 2-*RRT*1 by 2-*TT* (four profiles), 4-*RRT*1 by 2-*TT* (eight profiles), and 3-*RRT*1 by 3-*TT* (nine profiles).

Appendix B in S1 Appendix provides further empirical evidence that a lexicographic profiling method is necessary by showing in a regression setup that *TT* has predictive power on the probability of a subject correctly backward inducting only after we control for *RRT*1.

### Testing predictive power: 22 replications

In this section, the same test of predictive power is replicated in the 22 various tasks. Each time, the following exercise is conducted.

First, as already explained, we split the subjects into six profiles according to the 3-*RRT*1 by 2-*TT* partition. Data is presented in Table 3. For each task, we provide the number of subjects who played a task (*N*) and, for each profile, we compute the percentage of subjects who backward inducted (i.e., won a task). For profile *i* = 1, …, 6, this percentage is denoted as *P*_*i*_.

**Table 3:** Profiling with the 3-*RRT*1 by 2-*TT* partition.

Task	*N*	Profile 1	Profile 2	Profile 3	Profile 4	Profile 5	Profile 6
2.2.2	1,638	72.16%	93.91%	96.28%	99.36%	98.91%	100%
			(0.00)	(0.10)	(0.01)	(0.72)	(0.04)
2.2.3	1,729	75.53%	92.44%	95.37%	99.28%	98.33%	100%
			(0.00)	(0.07)	(0.00)	(0.86)	(0.01)
2.3.2	1,630	70.18%	93.95%	92.06%	100%	98.17%	99.62%
			(0.00)	(0.81)	(0.00)	(0.99)	(0.05)
2.3.3	1,637	66.41%	95.29%	98.80%	100%	98.91%	99.26%
			(0.00)	(0.01)	(0.04)	(0.96)	(0.33)
3.2.2	1,666	61.73%	89.24%	96.61%	99.37%	97.28%	99.63%
			(0.00)	(0.00)	(0.01)	(0.97)	(0.02)
3.3.2	1,647	65.65%	84.21%	96.37%	99.63%	95.41%	99.65%
			(0.00)	(0.00)	(0.00)	(1.00)	(0.00)
3.2.3	1,628	65.12%	85.61%	96.56%	99.23%	98.81%	100%
			(0.00)	(0.00)	(0.01)	(0.69)	(0.04)
3.3.3	1,638	57.20%	84.35%	97.08%	99.34%	97.35%	99.64%
			(0.00)	(0.00)	(0.02)	(0.97)	(0.01)
4.2.2	1,717	57.00%	87.05%	96.15%	100%	98.00%	98.92%
			(0.00)	(0.00)	(0.00)	(0.99)	(0.18)
2.2.2.2	1,660	7.22%	27.90%	75.18%	94.85%	95.57%	99.64%
			(0.00)	(0.00)	(0.00)	(0.35)	(0.00)
2.2.2.3	1,610	26.92%	60.81%	90.66%	97.84%	96.43%	100%
			(0.00)	(0.00)	(0.00)	(0.84)	(0.00)
2.2.3.2	1,674	25.18%	54.04%	87.83%	97.97%	98.22%	100%
			(0.00)	(0.00)	(0.00)	(0.41)	(0.01)
2.3.2.2	1,606	3.07%	7.99%	49.25%	79.79%	96.30%	99.24%
			(0.01)	(0.00)	(0.00)	(0.00)	(0.01)
3.2.2.2	1,575	14.13%	32.68%	82.61%	95.13%	94.98%	98.17%
			(0.00)	(0.00)	(0.00)	(0.53)	(0.02)
2.2.2.4	1,602	48.18%	68.08%	91.82%	94.85%	96.51%	98.89%
			(0.00)	(0.00)	(0.08)	(0.17)	(0.04)
2.2.4.2	1,673	15.75%	44.36%	80.36%	97.92%	96.13%	99.63%
			(0.00)	(0.00)	(0.00)	(0.90)	(0.00)
2.4.2.2	1,641	35.58%	67.25%	89.51%	93.84%	97.83%	99.26%
			(0.00)	(0.00)	(0.03)	(0.01)	(0.08)
4.2.2.2	1,614	13.46%	42.59%	79.06%	93.51%	95.93%	98.88%
			(0.00)	(0.00)	(0.00)	(0.11)	(0.02)
2.2.2.2.2	1,545	18.22%	61.87%	67.56%	93.31%	93.82%	100%
			(0.00)	(0.09)	(0.00)	(0.41)	(0.00)
3.2.2.2.2	1,550	14.83%	45.10%	64.64%	87.35%	94.12%	97.32%
			(0.00)	(0.00)	(0.00)	(0.00)	(0.04)
4.2.2.2.2	1,566	3.79%	21.79%	27.20%	56.11%	85.50%	93.46%
			(0.00)	(0.08)	(0.00)	(0.00)	(0.00)
2.2.2.2.2.2	1,580	17.29%	24.90%	32.21%	40.93%	82.13%	84.09%
			(0.02)	(0.03)	(0.02)	(0.00)	(0.27)

*Notes*. The table shows the probability that a subject with Profile *i* wins a given task (*P*_*i*_), where subjects are divided into six profiles in each task. The profiles are constructed by dividing *RRT*1 into terciles and, further, dividing each *RRT*1-tercile into two *TT*-halves. Profile 1 corresponds to the lowest *RRT*1-tercile and upper TT-half; Profile 2 corresponds to the lowest *RRT*1-tercile and lower *TT*-half; Profile 3 corresponds to the middle *RRT*1-tercile and upper *TT*-half; Profile 4 corresponds to the middle *RRT*1-tercile and lower *TT*-half; Profile 5 corresponds to the highest *RRT*1-tercile, upper *TT*-half; Profile 6 corresponds to the highest *RRT*1-tercile and lower *TT*-half. Finally, the values in parentheses correspond to the p-value of testing the null hypothesis that *P*_*i*−1_ ≥ *P*_*i*_.

The main hypothesis is that as the profile increases, *P*_*i*_ increases as well. More formally, we test the following null hypothesis *H*_0_ against the alternative hypothesis *H*_1_.

$$\begin{array}{c} H_{0}:P_{i-1}\geq P_{i} \end{array}$$ (1)

$$\begin{array}{c} H_{1}:P_{i-1}<P_{i} \end{array}$$ (2)

Since there are six profiles in each task, we conduct 5 comparisons per task. In Table 3, the value in parentheses below *P*_*i*_ corresponds to the p-value of our test.

Analysis of Table 3 indicates that in 97 out of the 110 pairwise comparisons it is true that *P*_*i*_ > *P*_*i*−*i*_. Moreover, in 89 out of these 97 comparisons, we reject *H*_0_ at the 10% level of significance or less. We conclude that higher profiles are more likely to backward induct and, consequently, establish the predictive power of the two-dimensional profiling.

### Predictive power of a profile constructed from a single task

In this section, we evaluate whether a profile calculated in a given task has predictive power in subsequently played tasks. This exercise sheds a light on how well a profile carries information into the future.

Recall that every subject in the sample is assigned a random sequence of 22 tasks. Let variable *Seq* = 1, …, 22 denote the order at which a given task appeared in a sequence. For *k* = 1, …, 21, let *Seq*−*k* correspond to a task that appeared *k* tasks before the task that was presented in the order *Seq*.

Therefore, for each value of *k* we construct a dataset in which we test if the profiles constructed at tasks played in the order *Seq* − *k* have predictive power in tasks played in the order *Seq*. For example, if we want to test if the profiles assigned to subjects are still informative 6 tasks after they are calculated, we set *k* = 6. Then, we test if the outcome (winning/losing) at tasks in *Seq* > 6 can be predicted with the profiles constructed in tasks *Seq*−6 (i.e., for the outcome of a task in *Seq* = 7 the corresponding profile is the one constructed in task *Seq* − *k* = 1, for the outcome of a task in *Seq* = 8 the corresponding profile is the one constructed in task *Seq* − *k* = 2, and so on).

As in Subsection “Testing predictive power: 22 replications,” we divide subjects into six profiles according to the 3-*RRT*1 by 2-*TT* partition. For each *k* = 1, …, 21, we estimate the following logit model. The choice of the particular econometric model is driven by the objective of this article: namely, evaluating how well the proposed profiling method predicts subjects’ behaviors. For profiling to be efficient and effective, it is important for the subjects’ descriptive characteristics that affect their probability to backward induct to be captured in their profiles. Then, when the method is applied, it is enough that the subjects’ data consists only of their choices.

$$\begin{array}{c} Logit\left(Y_{i}\right)=\alpha +\beta_{S}Seq+\beta_{C}Complex+\beta_{P}Profile_{i,k} \end{array}$$ (3)

*Y*_*i*_ is the dependent variable in the regression and captures whether the subject *i* backward inducted in the task appearing in the order *Seq* (*Y*_*i*_ = 1) or did not backward induct (*Y*_*i*_ = 0), *α* is the intercept, and *Seq* corresponds to the order in which a task appeared in the subject *i*’s sequence of tasks.

To control for individual task complexity, we introduce the variable *Complex* and, following [8], define it as the average response time at the first node calculated from all subjects who played a given task. We control for complexity because the order of tasks a subject plays is random. This implies that for a given value of *Seq*, various subjects participate in tasks of varying complexity. Following our discussion in Section “Lexicographic ranking,” the average response time at the first node is a natural measure of complexity as, especially in the context of tasks studied in this article, it is the reasoning at the first node that is crucial for a subject’s success. More complex tasks require more thinking time at the beginning. Importantly, [8] show that the average response time at the first node is an exceptionally reliable empirical measure of complexity.

Finally, *Profile*_*i*,*k*_, the main variable of interest, is a profile of the subject *i* calculated in a task that appeared in the order *Seq* − *k*. The values of variable *Profile*_*i*,*k*_ range from 1 (lowest profile) to 6 (highest profile). The positive coefficient of *Profile*_*i*,*k*_ verifies the predictive power of our profiling method.

Results are shown in Table 4, where we report in parentheses the p-values from heteroskedastic robust standard errors. (Estimations using clustered standard errors at the subjects’ level do not qualitatively modify any result).

**Table 4:** Predictive power of a profile constructed from a single task.

*k*	*N*	*Seq*	*Complex*	*Profile*_*i*,*k*_
1	28,283	0.069	-0.046	0.253
		(0.00)	(0.00)	(0.00)
2	22,956	0.046	-0.047	0.278
		(0.00)	(0.00)	(0.00)
3	19,157	0.038	-0.047	0.279
		(0.00)	(0.00)	(0.00)
4	16,707	0.038	-0.045	0.280
		(0.00)	(0.00)	(0.00)
5	14,579	0.034	-0.048	0.318
		(0.00)	(0.00)	(0.00)
6	12,827	0.033	-0.046	0.273
		(0.00)	(0.00)	(0.00)
7	11,360	0.021	-0.045	0.312
		(0.00)	(0.00)	(0.00)
8	10,089	0.023	-0.047	0.312
		(0.00)	(0.00)	(0.00)
9	8,931	0.010	-0.046	0.303
		(0.25)	(0.00)	(0.00)
10	7,906	0.000	-0.048	0.334
		(0.97)	(0.00)	(0.00)
11	6,985	0.004	-0.047	0.328
		(0.72)	(0.00)	(0.00)
12	6,133	0.002	-0.046	0.339
		(0.89)	(0.00)	(0.00)
13	5,356	0.017	-0.047	0.332
		(0.32)	(0.00)	(0.00)
14	4,609	0.006	-0.045	0.327
		(0.31)	(0.00)	(0.00)
15	3,921	-0.004	-0.046	0.338
		(0.88)	(0.00)	(0.00)
16	3,282	-0.024	-0.044	0.298
		(0.44)	(0.00)	(0.00)
17	2,676	-0.018	-0.046	0.338
		(0.67)	(0.00)	(0.00)
18	2,101	0.059	-0.043	0.273
		(0.33)	(0.00)	(0.00)
19	1,540	0.058	-0.051	0.311
		(0.55)	(0.00)	(0.00)
20	1,014	-0.101	-0.045	0.301
		(0.67)	(0.00)	(0.00)
21	488	–	-0.032	0.252
			(0.00)	(0.00)

*Notes*. The table shows the results from estimating a logit model. The dependent variable captures whether the subject backward inducted (*Y*_*i*_ = 1) or did not backward induct (*Y*_*i*_ = 0) in a given task. The independent variables are *Seq* (order in the sequence in which a task appeared for the subject), *Complex* (measure of task complexity), and *Profile*_*i*,*k*_ (profile of subject *i* calculated in the tasks that appeared in the order *Seq*−*k* of the sequence). The regression includes an intercept. The parentheses contain p-values calculated using heteroskedastic robust standard errors.

Table 4 provides three interesting results. First and most importantly, the coefficient for *Profile*_*i*,*k*_ is always positive and statistically significant at the 1% level or less. This means that a higher profile implies a higher likelihood of a subject correctly backward inducting. (This is also true in the extreme case of k = 21: a profile calculated in the 1st task the subject plays is still informative in the 22nd played task.) In fact, the coefficient for *Profile*_*i*,*k*_ is fairly stable across all *k* values, which suggests that the information contained in the profiles calculated at any prior task has a relatively similar predictive power.

Second, the order in which a task is played is important. The variable *Seq* shows that as a subject gains experience, the likelihood of correctly solving a task increases. Interestingly, that increment decreases and becomes non-significant once a subject has played nine or more tasks (*k* ≥ 9). This result points towards a limitation of non-supervised learning. However, an in-depth analysis of the impact of non-supervised learning is beyond the scope of this paper and would benefit from future research.

Third and finally, the coefficient of *Complex* is always negative and statistically significant. As expected, facing a more complex task decreases the likelihood of a subject correctly backward inducting.

Note that as we increase *k*, the number of observations used to estimate the model decreases. This is driven by two facts. First, a larger *k* leads to using a lower amount of data per subject. Imagine a subject who played all 22 tasks. For *k* = 1 the model uses 21 observations for this subject, while for *k* = 21 it uses only 1 observation. Second, the larger the *k* is the larger the impact of attrition as most subjects played fewer than the 22 tasks used in this paper. The problem of attrition is controlled for in Appendix A.3.1 in S1 Appendix, where we restrict the data analysis to subjects who played all tasks. Qualitatively, the results remain the same.

### Predictive power of a profile constructed from a group of consecutive tasks

In this section we study the predictive power of the proposed profiling method by constructing the profiles using data that consists of *g* consecutive tasks. The goal is to determine whether a multi-task profile has better predictive power than the single-task profile we have relied on so far. To make the results easier to compare across the different multi-task profiles, we fix the sequence of tasks in which the predictive power of the profiles is tested.

We apply our profiling method using the first *g* tasks (with *g* = 1, …, 9) and test its predictive power in all tasks played in the second part of the sequence of tasks a subject is assigned to (i.e., from *Seq* = 11 to *Seq* = 22). Given that every subject receives a randomly assigned sequence of tasks and every task has a different distribution of *RRT*1 and *TT* (see Table 1), we normalize these metrics by applying the standard min-max normalization method that maps the distribution of the variables to the [0, 1] interval.

More precisely, for each task *s* and each subject *i*, we compute the following variables.

$$\begin{array}{ccc} & & RRT1_{i,s}^{min-max}=\frac{RRT1_{i,s}-min\left(RRT1_{s}\right)}{max\left(RRT1_{s}\right)-min\left(RRT1_{s}\right)} \end{array}$$ (4)

$$\begin{array}{ccc} & & TT_{i,s}^{min-max}=\frac{TT_{i,s}-min\left(TT_{s}\right)}{max\left(TT_{s}\right)-min\left(TT_{s}\right)} \end{array}$$ (5)

Then, for each group of *g*-tasks (where *g* = 1, …, 9) we calculate for each subject the following variables.

$$\begin{array}{ccc} & & RRT1_{i}^{g}=\frac{1}{g}\sum_{s=1}^{g}RRT1_{i,s}^{min-max} \end{array}$$ (6)

$$\begin{array}{ccc} & & TT_{i}^{g}=\frac{1}{g}\sum_{s=1}^{g}TT_{i,s}^{min-max} \end{array}$$ (7)

Then, for each *g*, we use the subjects’ vectors $\left(RRT1_{i}^{g},TT_{i}^{g}\right)$ to create 6 profiles using the two-dimensional profiling with the 3-$RRT1_{i}^{g}$ by 2-$TT_{i}^{g}$ division. (This method is in the same spirit of the technique used when we partitioned subjects into the six profiles in Sections “Testing predictive power: 22 replications” and “Predictive power of a profile constructed from a single game”).

For each *g*, we test the predictive power of our profiling method by estimating the following logit model.

$$\begin{array}{c} Logit\left(Y_{i}\right)=\alpha +\beta_{S}Seq+\beta_{C}Complex+\beta_{P}Profile_{i,g} \end{array}$$ (8)

In the model (8), *Seq* > 10 and *g* = 1, …, 9, and the variables *Y*_*i*_, *Seq*, and *Complex* are the same as defined in the model described in (3) and discussed in Section “Predictive power of a profile constructed from a single game.” For each *g*, the variable *Profile*_*i*,*g*_ corresponds to the subject’s *i* profile calculated using the first *g* tasks. The positive coefficient of *Profile*_*i*,*g*_ confirms the predictive power of our profiling method. Results are in Table 5 below.

**Table 5:** Predictive power of a profile constructed from a group of consecutive tasks.

*g*	*N*	*Seq*	*Complex*	*Profile*_*i*,*g*_
1	7,868	0.008	-0.046	0.294
		(0.43)	(0.00)	(0.00)
2	7,310	0.009	-0.048	0.342
		(0.82)	(0.00)	(0.00)
3	6,882	0.002	-0.049	0.399
		(0.85)	(0.00)	(0.00)
4	6,538	-0.005	-0.049	0.434
		(0.68)	(0.00)	(0.00)
5	6,229	0.000	-0.050	0.504
		(0.94)	(0.00)	(0.00)
6	6,013	-0.005	-0.051	0.521
		(0.68)	(0.00)	(0.00)
7	5,820	-0.006	-0.051	0.553
		(0.65)	(0.00)	(0.00)
8	5,609	-0.001	-0.051	0.544
		(0.91)	(0.00)	(0.00)
9	5,521	0.001	-0.051	0.573
		(0.92)	(0.00)	(0.00)

*Notes*. The table shows the results from estimating a logit model. The dependent variable captures whether the subject backward inducted (*Y*_*i*_ = 1) or did not backward induct (*Y*_*i*_ = 0) in a given task. The independent variables are *Seq* (order in the sequence in which a task appeared for the subject), *Complex* (measure of task complexity), and *Profile*_*i*,*g*_ (profile of subject *i* calculated using the first *g* tasks the subject played). The regression includes an intercept. The parentheses contain p-values calculated using heteroskedastic robust standard errors.

Consistent with our previous results, the variable *Complex* is always negative and statistically significant. The variable *Seq* is always insignificant since we are only estimating the likelihood of a subject correctly solving a task after the 10th task has been played, and, from Table 4, we already know that *Seq* loses its predictive power after the 8th task.

The most important result pertains to the main variable of interest in this exercise, namely the multi-task profile, *Profile*_*i*,*g*_. The coefficient *β*_*P*_ is significant for all values of *g* (from 1 to 9). The results show that the more tasks we use to construct the profiles, the larger the coefficient. However, the coefficient’s increments after using 5 tasks (*g* = 5) become quite small. In fact, in Table A.3 in Appendix A.3.1 in S1 Appendix, where we restrict the analysis to subjects who played all tasks, the largest coefficient for *Profile*_*i*,*g*_ is found at *g* = 5.

This result implies that looking only at the first five tasks the subject plays offers almost all the necessary information to discriminate subjects by their likelihood to correctly backward induct in future tasks. This is captured in Table 6, which depicts the marginal effect of each profile (from Profile 1 to Profile 6) across the nine *g* groups of tasks used to create the 6 profiles.

**Table 6:** Marginal effects.

*g*	Profile 1	Profile 2	Profile 3	Profile 4	Profile 5	Profile 6
1	78.98%	83.03%	86.45%	89.30%	91.63%	93.51%
2	75.01%	80.26%	84.66%	88.25%	91.12%	93.37%
3	73.78%	79.99%	85.06%	89.05%	92.12%	94.42%
4	73.51%	80.26%	85.67%	89.83%	92.93%	95.17%
5	71.54%	79.64%	85.92%	90.56%	93.84%	96.07%
6	71.44%	79.80%	86.22%	90.90%	94.16%	96.34%
7	70.52%	79.50%	86.31%	91.19%	94.50%	96.66%
8	71.17%	79.87%	86.49%	91.24%	94.49%	96.62%
9	71.01%	80.16%	86.99%	91.79%	94.99%	97.02%

*Notes*. The estimated marginal effects correspond to the logit model presented in Table 5.

Table 6 shows that the difference in marginal effect between two consecutive profiles increases when we use more tasks to calculate the profiles. This means that increasing the number of tasks to construct the profiles improves their predictive capacity.

For example, for *g* = 1, *P*(*win*|*Profile* = 1) = 78.98% and *P*(*win*|*Profile* = 6) = 93.51%. On the other hand, for *g* = 5, *P*(*win*|*Profile* = 1) = 71.54% and *P*(*win*|*Profile* = 6) = 96.07%. Interestingly, after *g* = 5, the change in difference between the profiles’ marginal effects becomes quite small. For example, for *g* = 9, *P*(*win*|*Profile* = 1) = 70.01% and *P*(*win*|*Profile* = 6) = 97.02%, which is quite similar to that of *g* = 5.

**Fig 4:**
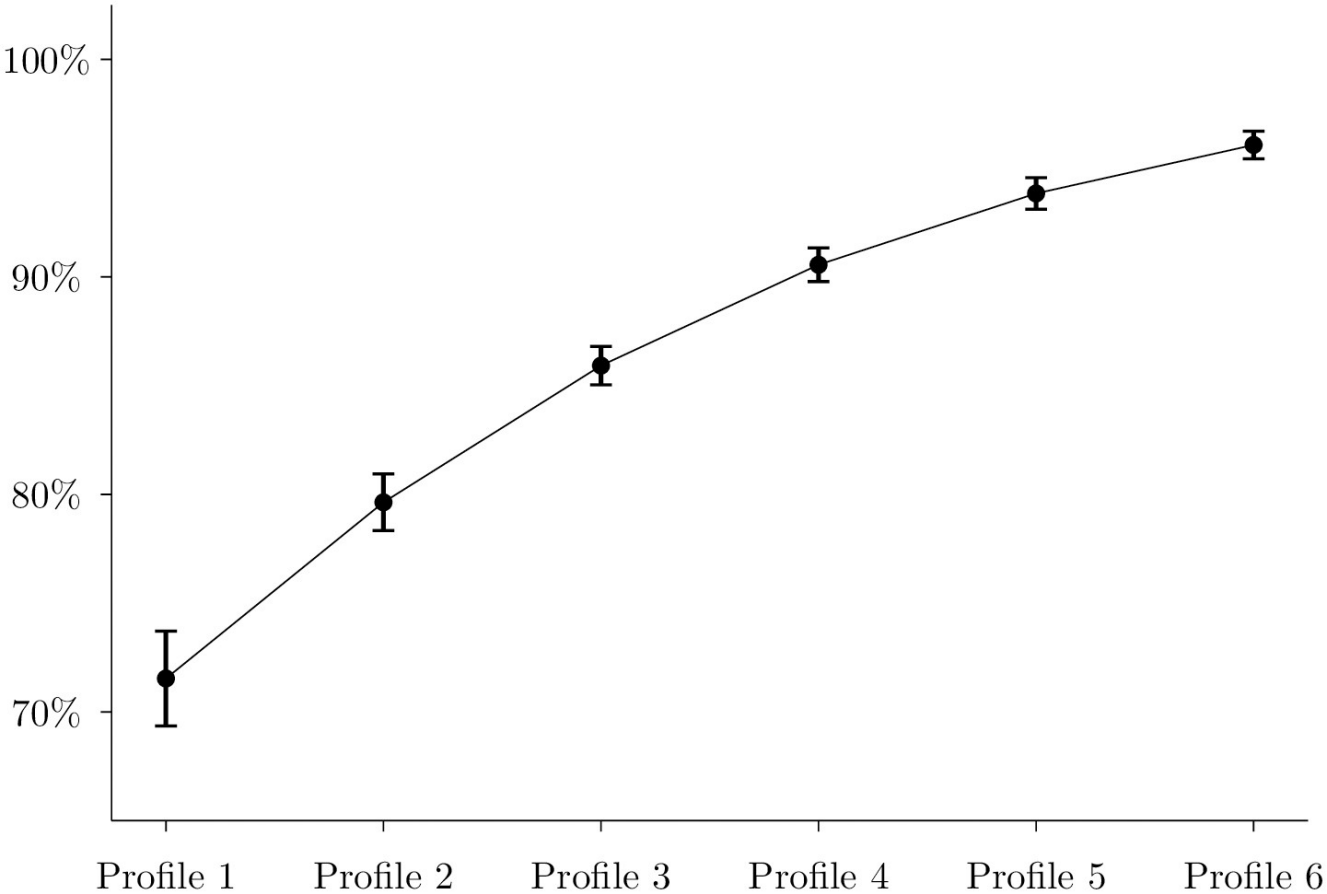
Marginal effects for *g* = 5. *Notes*. The estimated marginal effects and confidence intervals correspond to the logit model presented in Table 5 for the case *g* = 5.

Finally, Fig 4 below shows the marginal effects for *g* = 5 only, where we added the 95% confidence interval to show that the profiling method successfully distinguishes profiles by their likelihood to correctly solve the tasks.

In summary, the three exercises presented in this section in tandem with a series of robustness analyses included in Appendix A in S1 Appendix have established that the proposed profiling method has excellent predictive power. First, we showed that, in each of the 22 tasks analyzed in this article, the subjects’ profiles correctly distinguish subjects by their probability of backward inducting. Second, we confirmed that profiles carry information into the future. Finally, we found that while using more than one task to construct the profiles increases their predictive power, relying on just 5 tasks suffices.

Appendix A in S1 Appendix contains a series of robustness checks prepared for each exercise presented in this section. First, we control for attrition by restricting the data to subjects who completed all 22 tasks analyzed in this article. Second, using the whole sample, we consider alternative partitioning of subjects into profiles: 2−*RRT*1 by 2−*TT* (four profiles), 4−*RRT*1 by 2−*TT* (eight profiles), and 3 − *RRT*1 by 3 − *TT* (nine profiles). The optimal granularity of the partition used for profiling subjects depends on the dispersion of a problem’s response times. Our results suggest that if response times are condensed around the mean, a higher granularity might reduce accuracy, while if response times are more disperse, a lower granularity might reduce accuracy.

## Conclusions

This article develops a novel taxonomy of decision makers who solve dynamic tasks. The proposed profiling method is parsimonious and highly predictive of a subject’s likelihood to act in accordance with backward induction.

A profile consists of two dimensions. The first dimension relates the thinking process of a subject to the implementation of the backward induction algorithm. It is quantified as the relative response time at the first round (*RRT*1). The second dimension relates the thinking process of a subject to the effort that is required to implement the backward induction algorithm. It is measured by the total time spent on solving the task (*TT*).

Ann with (*RRT*1_*A*_, *TT*_*A*_) has a higher profile than Bob with (*RRT*1_*B*_, *TT*_*B*_) if she is either savvier (i.e., *RRT*1_*A*_ > *RRT*1_*B*_) or they are equally savvy but she is faster (i.e., if *RRT*1_*A*_ = *RRT*1_*B*_, then *TT*_*A*_ < *TT*_*B*_). We hypothesize that higher profiles are more likely to behave in accordance with backward induction.

To test the predictive power of the proposed profiling, we use a database generated by *Blues and Reds*, a mobile app for Android and iOS that was developed with the intention to take advantage of the omnipresence of mobile technology. The objective of *Blues and Reds* is to conduct mobile experiments, that is, experiments in which people install the app on their mobile devices and become the subjects of an experiment by using the app.

The database used in this paper consists of 35,826 observations from 6,463 subjects located in 141 countries. The experiment consists of 22 dynamic tasks with perfect and complete information played by human subjects against Artificial Intelligence. Subjects either win or lose, and winning is indicative of backward inducting. For each subject and each task, *Blues and Reds* records the response time at each round of the task and whether the subject wins.

A variety of empirical exercises accompanied by several robustness checks offer support for our main hypothesis and validate the predictive power of our profiling method: higher profiles are more likely to make choices consistent with the backward induction algorithm.

## Supporting information

S1 Appendix (ZIP)

S1 File (PDF)

S2 File (ZIP)
