# Supplementary material for: Profiling dynamic decision-makers
Source: PLoS One. 2022 Apr 14;17(4):e0266366. doi: 10.1371/journal.pone.0266366 (PMC9009624; doi:10.1371/journal.pone.0266366)

A Appendix: robustness checks

This appendix provides robustness checks for the results presented in Section "Profiles: Predicting behavior." First, for completeness purposes, in Section A.1, we list the three exercises that constitute the core of Section "Profiles: Predicting behavior." Second, in Section A.2, we describe the robustness analyses. Finally, in Section A.3, we present the results.

A.1 Empirical exercises in the main body

Exercise A in Section "Testing predictive power: 22 replications." Consider a task in our sample. First, we partition subjects into L quantiles in terms of $RRT1$. Second, each $RRT1$-quantile, is further divided into M quantiles in terms of TT. With the L-$RRT1$ by M-TT grid, we obtain $L \times M$ profiles. (In Section "Testing predictive power: 22 replications," we used $L = 3$ and $M = 2$.)

Third, for each profile i we compute the percentage of subjects with that profile who correctly backward inducted; this percentage is denoted as P_i. Finally, we test the following null hypothesis H_0 against its alternative H_1.

$$H_0 : P_{i-1} \geq P_i \tag{1}$$

$$H_1 : P_{i-1} < P_i \tag{2}$$

With $L \times M$ profiles in each task, we conduct $L \times M - 1$ comparisons per task. Our main interest lies in the number of these comparisons that reject H_0 as this supports our profiling method as a tool to predict behavior. The same analysis is replicated in each of the 22 tasks.

Exercise B in Section "Predictive power of a profile constructed from a single game." Every subject was assigned a random sequence of 22 tasks. Let $Seq = 1, ..., 22$ denote the order in which a given task appeared in a sequence. Let $k = 1, ..., 21$ indicate a task that appeared in the order $Seq - k$ in a sequence. For each value of k, we construct a dataset in which we verify whether the profiles constructed at tasks played in the order $Seq - k$ have predictive powers in tasks played in the order Seq.

Consider a task in our sample. First, as in Exercise A, we use the L-$RRT1$ by M-TT grid to partition subjects into $L \times M$ profiles. Second, for each $k = 1, ..., 21$, we estimate the

following logit model.

$$Logit(Y_i) = \alpha + \beta_S Seq + \beta_C Complex + \beta_P Profile_{i,k} \quad (3)$$

Y_i is the dependent variable in the regression and captures whether the subject i backward inducted in the task appearing in the order Seq ($Y_i = 1$) or did not backward induct ($Y_i = 0$), α is the intercept, Seq corresponds to the order in which a task appeared in the subject i's sequence of tasks, and $Complex$ captures task complexity.

The main variable of interest is $Profile_{i,k}$, a profile of subject i calculated in the task that appears in the order Seq–k. The coefficient of $Profile_{i,k}$ being positive verifies the predictive power of our profiling method.

Exercise C in Section "Predictive power of a pro

le constructed from a group of consecutive tasks." In Exercise B, we rely on only one task to construct the subjects' profiles. In Exercise C, we expand on this approach and use a group g of tasks to build the profiles. In particular, we consider $g = 1, ..., 9$ and test how well the profiles predict the subjects' behavior in tasks in the order $Seq = 11, ..., 22$. To address the fact that subjects are assigned different sequences, we normalize the metrics $RRT1$ and TT using the min-max method.

As in Exercises B and C, we use the L-$RRT1$ by M-TT grid to partition subjects into $L \times M$ profiles. Next, for each $g = 1, ..., 9$, we estimate the following logit model.

$$Logit(Y_i) = \alpha + \beta_S Seq + \beta_C Complex + \beta_P Profile_{i,g} \quad (4)$$

Variables Y_i, α, Seq, and $Complex$ are the same as in Exercise B (see model (3)). Our main focus is on $Profile_{i,g}$, a profile of subject i constructed using a group g of tasks. If the coefficient of $Profile_{i,g}$ is positive, then this establishes the predictive power of the proposed profiling method.

A.2 Robustness checks

In Section "Testing predictive power: 22 replications" of the main text, we conducted the empirical analysis using the whole sample and the 3-$RRT1$ by 2-TT grid (i.e., six profiles) and depicted the results from Exercises A, B, and C in Tables 3, 4, and 5, respectively. Now, we replicate all the three exercises using the following robustness checks.

First, since attrition is a potential problem in any experiment in which the subjects have to solve multiple tasks and are free to stop at any moment, it is imperative to analyze whether attrition has any impact on our results. Our sample contains 541 subjects who played all 22 tasks and 11,303 total observations.[1] In Section A.3.1, we limit our sample to these subjects. We find that all the qualitative results from the main body of the paper remain the same, which indicates that attrition has no significant impact on our study.

Second, using the whole sample, we consider alternative values of L and M to partition subjects into $L \times M$ profiles using the L-$RRT1$ by M-TT grid. We analyze three cases. The results with the 2-$RRT1$ by 2-TT partition (four profiles) are presented in Section A.3.2, those with the 4-$RRT1$ by 2-TT partition (eight profiles) are in Section A.3.3, and, finally, the 3-$RRT1$ by 3-TT partition (nine profiles) is discussed in Section A.3.4. Again, we find that all the qualitative results from the main body of the paper remain the same.

Third, in Section A.3.5, we trim data at 99% percentile (in the main body of the paper, we trimmed data at 95% percentile) and replicate the results presented in Tables 3, 4, and 5. The results are qualitatively the same as those obtained using the sample trimmed at the 95% percentile.

A.3 Results

A.3.1 Subjects who played all 22 tasks

Tables 3, 4, and 5 are replicated in Tables A.1, A.2, and A.3, respectively, by using the sub-sample that consists of 541 subjects who played all the 22 tasks.

Analysis of Table A.1 indicates that in 97 out of the 110 pairwise profile comparisons, we observe that $P_i > P_{i-1}$; this is the same qualitative result as presented in Table 3. The results in Tables A.2 and A.3 are qualitatively the same as those obtained using the full sample in Tables 4 and 5, respectively.

[1] Note that $541 \times 22 = 11,902$ observations, while we have 11,303 observations. The difference is because, from the data, we remove observations with TT above the 95th percentile, where we calculate the percentile within the entire sample.

Table A.1: Exercise A: subjects who played all 22 tasks.

Task	N	Profile 1	Profile 2	Profile 3	Profile 4	Profile 5	Profile 6
2.2.2	520	88.10%	96.25%	98.94%	99.05%	100%	100%
			(0.02)	(0.13)	(0.47)	(0.16)	(NA)
2.2.3	510	87.78%	96.30%	96.77%	100%	96.39%	100%
			(0.02)	(0.43)	(0.04)	(0.96)	(0.04)
2.3.2	521	82.14%	97.62%	96.34%	100%	100%	100%
			(0.00)	(0.68)	(0.04)	(NA)	(NA)
2.3.3	512	85.88%	98.78%	98.94%	100%	100%	98.85%
			(0.00)	(0.46)	(0.16)	(NA)	(0.84)
3.2.2	519	77.27%	89.47%	100%	100%	96.88%	100%
			(0.02)	(0.00)	(NA)	(0.96)	(0.04)
3.3.2	524	78.41%	92.47%	100%	100%	94.57%	100%
			(0.00)	(0.00)	(NA)	(0.99)	(0.01)
3.2.3	520	77.33%	95.70%	100%	97.70%	97.87%	100%
			(0.00)	(0.02)	(0.92)	(0.47)	(0.08)
3.3.3	510	78.82%	94.32%	98.73%	98.78%	98.89%	100%
			(0.00)	(0.06)	(0.49)	(0.47)	(0.16)
4.2.2	514	81.71%	95.70%	98.78%	100%	100%	100%
			(0.00)	(0.10)	(0.16)	(NA)	(NA)
2.2.2.2	511	20.23%	50.00%	82.93%	97.75%	96.39%	98.84%
			(0.00)	(0.00)	(0.00)	(0.70)	(0.15)
2.2.2.3	513	46.59%	80.72%	93.18%	100%	98.81%	100%
			(0.00)	(0.01)	(0.01)	(0.84)	(0.16)
2.2.3.2	515	41.86%	82.61%	88.10%	100%	100%	100%
			(0.00)	(0.15)	(0.00)	(NA)	(NA)
2.3.2.2	512	10.47%	32.14%	80.00%	100%	97.65%	100%
			(0.00)	(0.00)	(0.00)	(0.92)	(0.08)
3.2.2.2	517	34.52%	58.62%	96.47%	100%	94.19%	98.82%
			(0.00)	(0.00)	(0.04)	(0.99)	(0.05)
2.2.2.4	517	63.64%	89.02%	93.41%	100%	100%	100%
			(0.00)	(0.16)	(0.01)	(NA)	(NA)
2.2.4.2	517	29.41%	68.24%	93.18%	100%	96.47%	98.82%
			(0.00)	(0.00)	(0.01)	(0.96)	(0.16)
2.4.2.2	511	61.91%	78.82%	95.35%	97.70%	98.81%	100%
			(0.01)	(0.00)	(0.20)	(0.29)	(0.16)
4.2.2.2	515	32.56%	60.67%	90.70%	96.34%	98.84%	100%
			(0.00)	(0.00)	(0.07)	(0.15)	(0.16)
2.2.2.2.2	510	27.38%	61.18%	79.07%	98.84%	97.67%	100%
			(0.00)	(0.00)	(0.00)	(0.72)	(0.08)
3.2.2.2.2	503	34.12%	53.01%	86.75%	96.43%	94.12%	96.39%
			(0.01)	(0.00)	(0.01)	(0.76)	(0.25)
4.2.2.2.2	508	9.41%	25.00%	48.24%	76.19%	91.86%	94.05%
			(0.00)	(0.00)	(0.00)	(0.00)	(0.29)
2.2.2.2.2.2	504	19.05%	30.95%	53.01%	62.35%	92.94%	93.98%
			(0.04)	(0.00)	(0.11)	(0.00)	(0.39)

Notes. The table shows the probability P_i of Profile i winning a task. The values in parentheses correspond to the p-value of testing the null hypothesis $H_0 : P_{i-1} \geq P_i$ against its alternative $H_1 : P_{i-1} < P_i$. When $P_i = P_{i-1} = 100\%$, we report NA.

Table A.2: Exercise B: subjects who played all 22 tasks.

k	N	Seq	$Complex$	$Profile_{i,k}$
1	10,287	0.040	-0.034	0.279
		(0.00)	(0.00)	(0.00)
2	9,818	0.030	-0.035	0.328
		(0.00)	(0.00)	(0.00)
3	9,332	0.028	-0.034	0.301
		(0.00)	(0.00)	(0.00)
4	8,842	0.026	-0.033	0.290
		(0.00)	(0.00)	(0.00)
5	8,374	0.023	-0.034	0.332
		(0.00)	(0.00)	(0.00)
6	7,862	0.025	-0.034	0.257
		(0.00)	(0.00)	(0.00)
7	7,380	0.012	-0.034	0.305
		(0.14)	(0.00)	(0.00)
8	6,884	0.024	-0.034	0.303
		(0.01)	(0.00)	(0.00)
9	6,373	0.009	-0.034	0.306
		(0.38)	(0.00)	(0.00)
10	5,884	0.005	-0.034	0.352
		(0.69)	(0.00)	(0.00)
11	5,379	0.002	-0.034	0.326
		(0.91)	(0.00)	(0.00)
12	4,880	-0.002	-0.033	0.311
		(0.90)	(0.00)	(0.00)
13	4,392	0.012	-0.034	0.322
		(0.53)	(0.00)	(0.00)
14	3,882	0.008	-0.033	0.311
		(0.72)	(0.00)	(0.00)
15	3,389	-0.002	-0.033	0.316
		(0.95)	(0.00)	(0.00)
16	2,892	-0.021	-0.031	0.282
		(0.52)	(0.00)	(0.00)
17	2,406	0.004	-0.033	0.344
		(0.93)	(0.00)	(0.00)
18	1,921	0.075	-0.031	0.249
		(0.24)	(0.00)	(0.00)
19	1,444	0.059	-0.036	0.283
		(0.55)	(0.00)	(0.00)
20	972	-0.092	-0.032	0.313
		(0.71)	(0.00)	(0.00)
21	488	–	-0.024	0.241
			(0.00)	(0.00)

Notes. The table shows the results from estimating a logit model. The dependent variable captures whether the subject backward inducted ($Y_i = 1$) or did not backward induct ($Y_i = 0$) in a given task. The independent variables are Seq (order in the sequence in which a task appeared for the subject), $Complex$ (measure of task complexity), and $Profile_{i,k}$ (profile of subject i calculated in the tasks that appeared in the order $Seq - k$ of the sequence). The regression includes an intercept. The parentheses contain p-values calculated using heteroskedastic robust standard errors.

Table A.3: Exercise C: subjects who played all 22 tasks.

g	N	Seq	$Complex$	$Profile_{i,g}$
1	5,836	0.012	-0.033	0.261
		(0.34)	(0.00)	(0.00)
2	5,399	0.012	-0.034	0.363
		(0.34)	(0.00)	(0.00)
3	5,043	0.003	-0.035	0.472
		(0.82)	(0.00)	(0.00)
4	4,807	-0.005	-0.035	0.501
		(0.72)	(0.00)	(0.00)
5	4,620	-0.003	0.037	0.597
		(0.82)	(0.00)	(0.00)
6	4,482	-0.002	-0.037	0.572
		(0.91)	(0.00)	(0.00)
7	4,378	-0.003	-0.038	0.560
		(0.86)	(0.00)	(0.00)
8	4,231	-0.002	-0.037	0.564
		(0.90)	(0.00)	(0.00)
9	4,186	-0.001	-0.038	0.581
		(0.93)	(0.00)	(0.00)

Notes. The table shows the results from estimating a logit model. The dependent variable captures whether the subject backward inducted ($Y_i = 1$) or did not backward induct ($Y_i = 0$) in a given task. The independent variables are *Seq* (order in the sequence in which a task appeared for the subject), *Complex* (measure of task complexity), and $Profile_{i,g}$ (profile of subject i calculated using the first g tasks the subject played). The regression includes an intercept. The parentheses contain p-values calculated using heteroskedastic robust standard errors.

A.3.2 Alternative partition: 2-$RRT1$ by 2-TT

Tables 3, 4, and 5 are replicated in Tables A.4, A.5, and A.6, respectively using the 2-$RRT1$ by 2-TT partition of subjects into four profiles.

Analysis of Table A.4 indicates that in 64 out of the 66 pairwise profile comparisons, we observe that $P_i > P_{i-1}$. The difference is statistically significant at the 10% level or less in 62 out of the 64 comparisons. On the other hand, in none of the 2 comparisons in which a higher profile is not more likely to outperform the lower profile, the difference is statistically significant. In other words, the results in Table A.4 are qualitatively the same as the results presented in Table 3. It also is the case that the results in Tables A.5 and A.6 are qualitatively the same as the results depicted in Tables 4 and 5, respectively.

Table A.4: Exercise A: 2-$RRT1$ by 2-TT partition.

Task	N	Profile 1	Profile 2	Profile 3	Profile 4
2.2.2	1,638	80.73%	95.88%	97.83%	99.5%
			(0.00)	(0.05)	(0.02)
2.2.3	1,729	81.88%	94.57%	98.17%	100%
			(0.00)	(0.00)	(0.00)
2.3.2	1,630	75.80%	94.76%	96.79%	99.07%
			(0.00)	(0.07)	(0.01)
2.3.3	1,637	78.86%	96.50%	98.53%	99.76%
			(0.00)	(0.03)	(0.03)
3.2.2	1,666	69.81%	93.42%	97.86%	99.31%
			(0.00)	(0.00)	(0.04)
3.3.2	1,647	75.29%	90.34%	95.67%	99.48%
			(0.00)	(0.00)	(0.00)
3.2.3	1,628	75.77%	91.95%	98.21%	99.26%
			(0.00)	(0.00)	(0.09)
3.3.3	1,638	71.14%	90.14%	97.26%	99.76%
			(0.00)	(0.00)	(0.00)
4.2.2	1,717	68.79%	92.35%	97.85%	99.05%
			(0.00)	(0.00)	(0.07)
2.2.2.2	1,660	13.13%	63.22%	93.89%	97.12%
			(0.00)	(0.00)	(0.01)
2.2.2.3	1,610	46.43%	75.12%	94.85%	99.49%
			(0.00)	(0.00)	(0.00)
2.2.3.2	1,674	43.12%	70.49%	96.61%	99.29%
			(0.00)	(0.00)	(0.00)
2.3.2.2	1,606	8.52%	31.07%	90.82%	96.53%
			(0.00)	(0.00)	(0.00)
3.2.2.2	1,575	31.35%	54.86%	93.22%	98.97%
			(0.00)	(0.00)	(0.00)
2.2.2.4	1,602	61.38%	75.25%	96.71%	98.04%
			(0.00)	(0.00)	(0.12)
2.2.4.2	1,673	28.98%	68.60%	94.76%	98.80%
			(0.00)	(0.00)	(0.00)
2.4.2.2	1,641	52.69%	74.24%	96.66%	97.77%
			(0.00)	(0.00)	(0.17)
4.2.2.2	1,614	31.00%	60.10%	93.15%	96.99%
			(0.00)	(0.00)	(0.01)
2.2.2.2.2	1,545	31.07%	70.44%	89.82%	97.69%
			(0.00)	(0.00)	(0.00)
3.2.2.2.2	1,550	29.08%	53.26%	91.19%	94.86%
			(0.00)	(0.00)	(0.02)
4.2.2.2.2	1,566	7.42%	28.83%	79.28%	76.28%
			(0.00)	(0.00)	(0.84)
2.2.2.2.2.2	1,580	21.66%	29.59%	74.94%	61.36%

Notes. The table shows the probability P_i of Profile i winning a task. The values in parentheses correspond to the p-value of testing the null hypothesis $H_0 : P_{i-1} \geq P_i$ against its alternative $H_1 : P_{i-1} < P_i$.

Table A.5: Exercise B: 2-$RRT1$ by 2-TT partition.

k	N	Seq	$Complex$	$Profile_{i,k}$
1	28,283	0.069	-0.045	0.334
		(0.00)	(0.00)	(0.00)
2	22,956	0.046	-0.046	0.363
		(0.00)	(0.00)	(0.00)
3	19,157	0.039	-0.047	0.366
		(0.00)	(0.00)	(0.00)
4	16,707	0.039	-0.045	0.364
		(0.00)	(0.00)	(0.00)
5	14,579	0.034	-0.047	0.428
		(0.00)	(0.00)	(0.00)
6	12,827	0.032	-0.046	0.352
		(0.00)	(0.00)	(0.00)
7	11,360	0.021	-0.045	0.400
		(0.00)	(0.00)	(0.00)
8	10,089	0.025	-0.046	0.400
		(0.00)	(0.00)	(0.00)
9	8,931	0.012	-0.045	0.407
		(0.17)	(0.00)	(0.00)
10	7,906	0.003	-0.047	0.434
		(0.74)	(0.00)	(0.00)
11	6,985	0.005	-0.047	0.443
		(0.63)	(0.00)	(0.00)
12	6,133	0.003	-0.045	0.433
		(0.81)	(0.00)	(0.00)
13	5,356	0.017	-0.047	0.447
		(0.30)	(0.00)	(0.00)
14	4,609	0.008	-0.045	0.458
		(0.68)	(0.00)	(0.00)
15	3,921	0.001	-0.045	0.436
		(0.98)	(0.00)	(0.00)
16	3,282	-0.021	-0.044	0.350
		(0.50)	(0.00)	(0.00)
17	2,676	-0.012	-0.045	0.448
		(0.77)	(0.00)	(0.00)
18	2,101	0.058	-0.042	0.311
		(0.98)	(0.00)	(0.00)
19	1,540	0.063	-0.051	0.369
		(0.51)	(0.00)	(0.00)
20	1,014	-0.087	-0.044	0.352
		(0.70)	(0.00)	(0.00)
21	488	–	-0.033	0.365
			(0.00)	(0.00)

Notes. The table shows the results from estimating a logit model. The dependent variable captures whether the subject backward inducted ($Y_i = 1$) or did not backward induct ($Y_i = 0$) in a given task. The independent variables are Seq (order in the sequence in which a task appeared for the subject), $Complex$ (measure of task complexity), and $Profile_{i,k}$ (profile of subject i calculated in the tasks that appeared in the order $Seq - k$ of the sequence). The regression includes an intercept. The parentheses contain p-values calculated using heteroskedastic robust standard errors.

Table A.6: Exercise C: 2-$RRT1$ by 2-TT partition.

g	N	Seq	$Complex$	$Profile_{i,g}$
1	7,868	0.009	-0.046	0.371
		(0.96)	(0.00)	(0.00)
2	7,310	0.010	-0.048	0.473
		(0.99)	(0.00)	(0.00)
3	6,882	0.005	-0.049	0.540
		(0.67)	(0.00)	(0.00)
4	6,538	0.000	-0.048	0.561
		(0.93)	(0.00)	(0.00)
5	6,229	0.000	-0.048	0.621
		(0.97)	(0.00)	(0.00)
6	6,013	-0.004	-0.048	0.648
		(0.76)	(0.00)	(0.00)
7	5,820	-0.003	-0.049	0.648
		(0.78)	(0.00)	(0.00)
8	5,609	0.000	-0.050	0.676
		(0.98)	(0.00)	(0.00)
9	5,521	0.000	-0.049	0.685
		(0.98)	(0.00)	(0.00)

Notes. The table shows the results from estimating a logit model. The dependent variable captures whether the subject backward inducted ($Y_i = 1$) or did not backward induct ($Y_i = 0$) in a given task. The independent variables are *Seq* (order in the sequence in which a task appeared for the subject), *Complex* (measure of task complexity), and $Profile_{i,g}$ (profile of subject i calculated using the first g tasks the subject played). The regression includes an intercept. The parentheses contain p-values calculated using heteroskedastic robust standard errors.

A.3.3 Alternative partition: 4-$RRT1$ by 2-TT

Tables 3, 4, and 5 are replicated in Tables A.7, A.8, and A.9, respectively using the 4-$RRT1$ by 2-TT partition of subjects into four profiles.

Analysis of Table A.7 indicates that in 123 out of the 154 pairwise profile comparisons we observe that $P_i > P_{i-1}$. In 107 out of the 123 comparisons, the difference is statistically significant at the 10% level or less. On the other hand, in 18 of the 23 comparisons in which a higher profile is not more likely to outperform the lower profile the difference is statistically significant.[2] In other words, the results in Table A.7 are qualitatively the same as the results presented in Table 3. It also is the case that the results in Tables A.8 and A.9 are qualitatively the same as the results depicted in Tables 4 and 5, respectively.

[2]This exercise shows that a more granular profiling might reduce accuracy since it makes it harder to differentiate between subjects belonging to different $RRT1$-quartiles. The optimal division of $RRT1$ and TT should depend on the quantity and dispersion of the $RRT1$ and TT data in a given task. Nevertheless, the two-dimensional profile still accurately ranks subjects in each task by their probability to correctly backward induct.

Table A.7: Exercise A: 4-$RRT1$ by 2-TT partition.

Task	N	Profile 1	Profile 2	Profile 3	Profile 4	Profile 5	Profile 6	Profile 7	Profile 8
2.2.2	1,638	70.64%	93.10%	96.94%	99.37%	96.17%	99.49%	98.97%	100%
			(0.00)	(0.03)	(0.04)	(0.98)	(0.01)	(0.72)	(0.08)
2.2.3	1,729	67.93%	90.56%	95.09%	99.04%	98.10%	100%	98.10%	100%
			(0.00)	(0.03)	(0.01)	(0.79)	(0.02)	(0.98)	(0.02)
2.3.2	1,630	65.14%	90.14%	92.39%	99.41%	93.56%	99.58%	98.43%	100%
			(0.00)	(0.21)	(0.00)	(1.00)	(0.00)	(0.88)	(0.04)
2.3.3	1,637	59.79%	89.39%	97.13%	100%	97.69%	100%	99.49%	99.52%
			(0.00)	(0.00)	(0.01)	(0.99)	(0.01)	(0.84)	(0.48)
3.2.2	1,666	54.63%	83.16%	94.76%	97.73%	97.71%	99.53%	97.55%	99.54%
			(0.00)	(0.00)	(0.06)	(0.51)	(0.05)	(0.95)	(0.05)
3.3.2	1,647	55.83%	78.71%	96.15%	97.81%	95.00%	100%	97.07%	98.46%
			(0.00)	(0.00)	(0.15)	(0.94)	(0.00)	(0.99)	(0.17)
3.2.3	1,628	58.25%	81.16%	95.19%	99.57%	98.00%	98.64%	98.40%	100%
			(0.00)	(0.00)	(0.00)	(0.93)	(0.31)	(0.58)	(0.04)
3.3.3	1,638	50.25%	76.09%	95.31%	99.08%	96.15%	99.52%	98.45%	100%
			(0.00)	(0.00)	(0.01)	(0.98)	(0.01)	(0.85)	(0.04)
4.2.2	1,717	47.73%	84.72%	91.71%	98.52%	96.14%	100%	98.59%	99.10%
			(0.00)	(0.01)	(0.00)	(0.94)	(0.00)	(0.96)	(0.31)
2.2.2.2	1,660	7.66%	15.05%	46.22%	85.64%	90.50%	96.35%	96.23%	99.50%
			(0.01)	(0.00)	(0.00)	(0.06)	(0.01)	(0.53)	(0.01)
2.2.2.3	1,610	21.95%	48.47%	79.55%	95.77%	93.04%	99.50%	96.55%	99.49%
			(0.00)	(0.00)	(0.00)	(0.88)	(0.00)	(0.98)	(0.02)
2.2.3.2	1,674	17.91%	37.33%	78.37%	91.08%	93.52%	99.51%	99.05%	100%
			(0.00)	(0.00)	(0.00)	(0.17)	(0.00)	(0.71)	(0.08)
2.3.2.2	1,606	3.00%	5.94%	18.40%	53.30%	78.80%	97.12%	98.01%	99.51%
			(0.08)	(0.00)	(0.00)	(0.00)	(0.00)	(0.28)	(0.09)
3.2.2.2	1,575	9.00%	17.37%	63.90%	82.81%	90.95%	99.47%	95.34%	98.98%
			(0.01)	(0.00)	(0.00)	(0.01)	(0.00)	(0.99)	(0.02)
2.2.2.4	1,602	38.57%	62.18%	85.22%	89.64%	94.74%	99.03%	96.62%	99.00%
			(0.00)	(0.00)	(0.09)	(0.03)	(0.01)	(0.95)	(0.05)
2.2.4.2	1,673	12.38%	32.85%	59.43%	90.29%	91.71%	99.5%	96.24%	100%
			(0.00)	(0.00)	(0.00)	(0.31)	(0.00)	(0.99)	(0.00)
2.4.2.2	1,641	24.65%	63.24%	78.89%	90.69%	92.97%	97.24%	99.03%	99.50%
			(0.00)	(0.00)	(0.00)	(0.20)	(0.02)	(0.09)	(0.29)
4.2.2.2	1,614	10.61%	26.83%	57.64%	87.50%	86.29%	97.02%	97.13%	99.50%
			(0.00)	(0.00)	(0.00)	(0.64)	(0.00)	(0.47)	(0.03)
2.2.2.2.2	1,545	16.33%	55.26%	45.88%	86.98%	82.35%	97.51%	94.85%	100%
			(0.00)	(0.97)	(0.00)	(0.89)	(0.00)	(0.92)	(0.00)
3.2.2.2.2	1,550	9.57%	30.85%	47.06%	74.87%	84.18%	96.35%	93.78%	97.94%
			(0.00)	(0.00)	(0.00)	(0.01)	(0.00)	(0.88)	(0.02)
4.2.2.2.2	1,566	2.58%	17.17%	12.12%	40.93%	53.09%	75.25%	87.69%	94.90%
			(0.00)	(0.92)	(0.00)	(0.01)	(0.00)	(0.00)	(0.01)
2.2.2.2.2.2	1,580	13.02%	25.12%	29.80%	34.18%	46.73%	51.52%	86.87%	87.76%
			(0.00)	(0.15)	(0.18)	(0.01)	(0.17)	(0.00)	(0.40)

Notes. The table shows the probability P_i of Profile i winning a task. The values in parentheses correspond to the p-value of testing the null hypothesis $H_0 : P_{i-1} \geq P_i$ against its alternative $H_1 : P_{i-1} < P_i$.

Table A.8: Exercise B: 4-$RRT1$ by 2-TT partition.

k	N	Seq	$Complex$	$Profile_{i,k}$
1	28,283	0.069	-0.046	0.201
		(0.00)	(0.00)	(0.00)
2	22,956	0.046	-0.047	0.214
		(0.00)	(0.00)	(0.00)
3	19,157	0.038	-0.047	0.213
		(0.00)	(0.00)	(0.00)
4	16,707	0.039	-0.045	0.212
		(0.00)	(0.00)	(0.00)
5	14,579	0.035	-0.048	0.244
		(0.00)	(0.00)	(0.00)
6	12,827	0.034	-0.046	0.207
		(0.00)	(0.00)	(0.00)
7	11,360	0.022	-0.046	0.230
		(0.00)	(0.00)	(0.00)
8	10,089	0.024	-0.047	0.233
		(0.00)	(0.00)	(0.00)
9	8,931	0.012	-0.046	0.217
		(0.16)	(0.00)	(0.00)
10	7,906	0.001	-0.049	0.259
		(0.94)	(0.00)	(0.00)
11	6,985	0.005	-0.047	0.252
		(0.70)	(0.00)	(0.00)
12	6,133	0.002	-0.046	0.254
		(0.90)	(0.00)	(0.00)
13	5,356	0.015	-0.047	0.253
		(0.36)	(0.00)	(0.00)
14	4,609	0.005	-0.046	0.251
		(0.79)	(0.00)	(0.00)
15	3,921	-0.005	-0.046	0.253
		(0.86)	(0.00)	(0.00)
16	3,282	-0.027	-0.044	0.218
		(0.39)	(0.00)	(0.00)
17	2,676	-0.019	-0.046	0.259
		(0.65)	(0.00)	(0.00)
18	2,101	0.050	-0.043	0.215
		(0.41)	(0.00)	(0.00)
19	1,540	0.052	-0.051	0.227
		(0.59)	(0.00)	(0.00)
20	1,014	-0.080	-0.045	0.202
		(0.73)	(0.00)	(0.00)
21	488	–	-0.033	0.181
			(0.00)	(0.00)

Notes. The table shows the results from estimating a logit model. The dependent variable captures whether the subject backward inducted ($Y_i = 1$) or did not backward induct ($Y_i = 0$) in a given task. The independent variables are Seq (order in the sequence in which a task appeared for the subject), $Complex$ (measure of task complexity), and $Profile_{i,k}$ (profile of subject i calculated in the tasks that appeared in the order $Seq - k$ of the sequence). The regression includes an intercept. The parentheses contain p-values calculated using heteroskedastic robust standard errors.

Table A.9: Exercise C: 4-$RRT1$ by 2-TT partition.

g	N	Seq	$Complex$	$Profile_{i,g}$
1	7,868	0.008	-0.046	0.207
		(0.43)	(0.00)	(0.00)
2	7,310	0.008	-0.048	0.276
		(0.43)	(0.00)	(0.00)
3	6,882	0.000	-0.050	0.327
		(0.98)	(0.00)	(0.00)
4	6,538	-0.007	-0.050	0.350
		(0.54)	(0.00)	(0.00)
5	6,229	-0.005	0.052	0.418
		(0.70)	(0.00)	(0.00)
6	6,013	-0.007	-0.051	0.434
		(0.57)	(0.00)	(0.00)
7	5,820	-0.008	-0.053	0.449
		(0.51)	(0.00)	(0.00)
8	5,609	-0.005	-0.053	0.474
		(0.72)	(0.00)	(0.00)
9	5,521	-0.005	-0.053	0.499
		(0.71)	(0.00)	(0.00)

Notes. The table shows the results from estimating a logit model. The dependent variable captures whether the subject backward inducted ($Y_i = 1$) or did not backward induct ($Y_i = 0$) in a given task. The independent variables are Seq (order in the sequence in which a task appeared for the subject), $Complex$ (measure of task complexity), and $Profile_{i,g}$ (profile of subject i calculated using the first g tasks the subject played). The regression includes an intercept. The parentheses contain p-values calculated using heteroskedastic robust standard errors.

A.3.4 Alternative partition: 3-$RRT1$ by 3-TT

Tables 3, 4, and 5 are replicated in Tables A.10, A.11, and A.12, respectively using the 3-$RRT1$ by 3-TT partition of subjects into four profiles.

Analysis of Table A.10 indicates that in 148 out of the 176 pairwise profile comparisons we observe that $P_i > P_{i-1}$. In 104 out of the 148 results that support the profiling method, the difference is statistically significant at the 10% level or less. On the other hand, in only 13 of the 24 comparisons in which a higher profile is not more likely to outperform the lower profile, the difference is statistically significant.[3] In other words, the results in Table A.10 are qualitatively the same as the results presented in Table 3. It also is the case that the results in Tables A.11 and A.12 are qualitatively the same as the results depicted in Tables 4 and 5, respectively.

[3]As in Appendix A.3.3, the exercise in this section shows that a more granular profiling might reduce accuracy.

Table A.10: Exercise A: 3-$RRT1$ by 3-TT partition.

Task	N	Profile 1	Profile 2	Profile 3	Profile 4	Profile 5	Profile 6	Profile 7	Profile 8	Profile 9
2.2.2	1,638	67.55%	90.86%	93.75%	95.69%	98.68%	99.55%	98.82%	99.39%	100%
			(0.00)	(0.16)	(0.21)	(0.04)	(0.20)	(0.78)	(0.29)	(0.16)
2.2.3	1,729	71.78%	87.79%	93.47%	93.68%	99.39%	99.02%	97.99%	99.49%	100%
			(0.00)	(0.03)	(0.47)	(0.00)	(0.66)	(0.80)	(0.09)	(0.16)
2.3.2	1,630	63.78%	86.96%	95.40%	87.91%	100%	100%	97.71%	99.44%	99.44%
			(0.00)	(0.00)	(1.00)	(0.00)	(NA)	(0.98)	(0.09)	(0.50)
2.3.3	1,637	57.22%	90.72%	96.22%	98.38%	100%	100%	98.35%	99.46%	99.44%
			(0.00)	(0.01)	(0.10)	(0.04)	(NA)	(0.96)	(0.16)	(0.51)
3.2.2	1,666	55.56%	80.24%	88.95%	96.54%	97.79%	99.57%	97.14%	98.97%	99.36%
			(0.00)	(0.01)	(0.00)	(0.23)	(0.07)	(0.97)	(0.11)	(0.35)
3.3.2	1,647	54.95%	82.44%	85.55%	96.22%	98.91%	99.33%	95.34%	97.92%	99.46%
			(0.00)	(0.21)	(0.00)	(0.05)	(0.34)	(0.99)	(0.08)	(0.09)
3.2.3	1,628	58.20%	80.85%	90.57%	95.98%	97.93%	100%	98.27%	100%	100%
			(0.00)	(0.00)	(0.02)	(0.13)	(0.02)	(0.96)	(0.04)	(NA)
3.3.3	1,638	47.85%	77.92%	84.16%	95.75%	99.44%	99.52%	96.09%	99.46%	100%
			(0.00)	(0.07)	(0.00)	(0.01)	(0.46)	(0.99)	(0.02)	(0.16)
4.2.2	1,717	48.99%	78.17%	89.77%	94.87%	100%	100%	97.21%	99.07%	98.91%
			(0.00)	(0.00)	(0.03)	(0.00)	(NA)	(0.99)	(0.09)	(0.56)
2.2.2.2	1,660	7.98%	8.84%	35.87%	67.82%	90.28%	95.24%	93.96%	99.45%	99.46%
			(0.38)	(0.00)	(0.00)	(0.00)	(0.03)	(0.70)	(0.00)	(0.49)
2.2.2.3	1,610	21.23%	46.43%	64.52%	87.70%	97.48%	98.41%	95.48%	98.89%	100%
			(0.00)	(0.00)	(0.00)	(0.00)	(0.27)	(0.95)	(0.03)	(0.08)
2.2.3.2	1,674	19.79%	43.68%	54.70%	84.38%	98.38%	97.24%	98.37%	98.95%	100%
			(0.00)	(0.02)	(0.00)	(0.00)	(0.77)	(0.23)	(0.31)	(0.08)
2.3.2.2	1,606	3.35%	3.57%	9.61%	40.32%	74.14%	80.85%	94.94%	99.43%	98.90%
			(0.46)	(0.01)	(0.00)	(0.00)	(0.06)	(0.00)	(0.01)	(0.71)
3.2.2.2	1,575	10.53%	24.18%	34.71%	76.05%	94.02%	96.45%	94.86%	96.22%	98.84%
			(0.00)	(0.02)	(0.00)	(0.00)	(0.14)	(0.77)	(0.27)	(0.05)
2.2.2.4	1,602	37.02%	72.63%	64.37%	88.44%	96.59%	94.79%	96.00%	98.90%	98.25%
			(0.00)	(0.95)	(0.00)	(0.00)	(0.80)	(0.29)	(0.04)	(0.69)
2.2.4.2	1,673	11.35%	29.03%	50.85%	74.05%	94.36%	98.94%	95.75%	97.79%	100%
			(0.00)	(0.00)	(0.00)	(0.00)	(0.01)	(0.97)	(0.13)	(0.02)
2.4.2.2	1,641	28.89%	56.59%	69.31%	85.63%	94.41%	94.74%	96.65%	100%	98.94%
			(0.00)	(0.01)	(0.00)	(0.00)	(0.45)	(0.18)	(0.01)	(0.92)
4.2.2.2	1,614	10.92%	24.47%	47.73%	70.86%	93.09%	93.75%	95.43%	97.75%	98.91%
			(0.00)	(0.00)	(0.00)	(0.00)	(0.40)	(0.24)	(0.12)	(0.20)
2.2.2.2.2	1,545	16.76%	34.68%	69.23%	56.40%	89.82%	94.35%	91.81%	98.85%	100%
			(0.00)	(0.00)	(0.99)	(0.00)	(0.06)	(0.82)	(0.00)	(0.08)
3.2.2.2.2	1,550	12.72%	25.29%	51.46%	60.12%	80.22%	86.75%	93.10%	95.93%	98.24%
			(0.00)	(0.00)	(0.05)	(0.00)	(0.05)	(0.03)	(0.12)	(0.10)
4.2.2.2.2	1,566	2.27%	9.94%	25.86%	24.86%	38.98%	61.27%	84.48%	90.23%	93.68%
			(0.00)	(0.00)	(0.59)	(0.00)	(0.00)	(0.00)	(0.05)	(0.12)
2.2.2.2.2.2	1,580	13.22%	26.40%	23.43%	31.43%	37.64%	40.46%	82.49%	85.14%	81.71%
			(0.00)	(0.74)	(0.05)	(0.11)	(0.29)	(0.00)	(0.25)	(0.81)

Notes. The table shows the probability P_i of Profile i winning a task. The values in parentheses correspond to the p-value of testing the null hypothesis $H_0 : P_{i-1} \geq P_i$ against its alternative $H_1 : P_{i-1} < P_i$. When $P_i = P_{i-1} = 100\%$, we report NA.

Table A.11: Exercise B: 3-$RRT1$ by 3-TT partition.

k	N	Seq	$Complex$	$Profile_{i,k}$
1	28,283	0.069	-0.046	0.166
		(0.00)	(0.00)	(0.00)
2	22,956	0.045	-0.047	0.180
		(0.00)	(0.00)	(0.00)
3	19,157	0.038	-0.047	0.184
		(0.00)	(0.00)	(0.00)
4	16,707	0.038	-0.045	0.186
		(0.00)	(0.00)	(0.00)
5	14,579	0.034	-0.048	0.206
		(0.00)	(0.00)	(0.00)
6	12,827	0.033	-0.046	0.178
		(0.00)	(0.00)	(0.00)
7	11,360	0.021	-0.046	0.201
		(0.00)	(0.00)	(0.00)
8	10,089	0.022	-0.047	0.207
		(0.00)	(0.00)	(0.00)
9	8,931	0.009	-0.046	0.195
		(0.25)	(0.00)	(0.00)
10	7,906	0.000	-0.048	0.218
		(0.97)	(0.00)	(0.00)
11	6,985	0.003	-0.047	0.218
		(0.78)	(0.00)	(0.00)
12	6,133	0.002	-0.046	0.216
		(0.89)	(0.00)	(0.00)
13	5,356	0.016	-0.047	0.215
		(0.34)	(0.00)	(0.00)
14	4,609	0.006	-0.045	0.213
		(0.76)	(0.00)	(0.00)
15	3,921	-0.003	-0.046	0.222
		(0.90)	(0.00)	(0.00)
16	3,282	-0.024	-0.044	0.196
		(0.44)	(0.00)	(0.00)
17	2,676	-0.019	-0.046	0.218
		(0.66)	(0.00)	(0.00)
18	2,101	0.059	-0.043	0.179
		(0.33)	(0.00)	(0.00)
19	1,540	0.056	-0.051	0.197
		(0.56)	(0.00)	(0.00)
20	1,014	-0.093	-0.045	0.204
		(0.69)	(0.00)	(0.00)
21	488	–	-0.032	0.163
			(0.00)	(0.00)

Notes. The table shows the results from estimating a logit model. The dependent variable captures whether the subject backward inducted ($Y_i = 1$) or did not backward induct ($Y_i = 0$) in a given task. The independent variables are Seq (order in the sequence in which a task appeared for the subject), $Complex$ (measure of task complexity), and $Profile_{i,k}$ (profile of subject i calculated in the tasks that appeared in the order $Seq - k$ of the sequence). The regression includes an intercept. The parentheses contain p-values calculated using heteroskedastic robust standard errors.

Table A.12: Exercise C: 3-$RRT1$ by 3-TT partition.

g	N	Seq	$Complex$	$Profile_{i,g}$
1	7,868	0.008	-0.047	0.194
		(0.83)	(0.00)	(0.00)
2	7,310	0.009	-0.048	0.220
		(0.41)	(0.00)	(0.00)
3	6,882	0.002	-0.049	0.267
		(0.87)	(0.00)	(0.00)
4	6,538	-0.004	-0.049	0.280
		(0.72)	(0.00)	(0.00)
5	6,229	-0.003	-0.050	0.324
		(0.82)	(0.00)	(0.00)
6	6,013	-0.005	-0.051	0.338
		(0.69)	(0.00)	(0.00)
7	5,820	-0.004	-0.051	0.353
		(0.73)	(0.00)	(0.00)
8	5,609	-0.001	-0.051	0.350
		(0.91)	(0.00)	(0.00)
9	5,521	0.001	-0.051	0.372
		(0.91)	(0.00)	(0.00)

Notes. The table shows the results from estimating a logit model. The dependent variable captures whether the subject backward inducted ($Y_i = 1$) or did not backward induct ($Y_i = 0$) in a given task. The independent variables are *Seq* (order in the sequence in which a task appeared for the subject), *Complex* (measure of task complexity), and $Profile_{i,g}$ (profile of subject i calculated using the first g tasks the subject played). The regression includes an intercept. The parentheses contain p-values calculated using heteroskedastic robust standard errors.

A.3.5 Data trimmed at the 99% percentile

Tables 3, 4, and 5 are replicated in Tables A.13, A.14, and A.15, respectively, by using a sample that consists of 37,311. In this larger sample data in each task has been trimmed at the 99% percentile of total time spent solving the task.

The maximum time spent solving a task in this sample is 85,467 seconds (almost a day). The maximum time spent solving a task in the original sample trimmed at the 95% percentile is 534 seconds (less than 10 minutes). This difference might be due to a subject stop playing *Blues and Reds* and restarting in a later time.

Analysis of Table A.13 indicates that in 94 out of the 110 pairwise profile comparisons, we observe that $P_i > P_{i-i}$; this is the same qualitative result as presented in Table 3. The results in Tables A.14 and A.15 are qualitatively the same as those obtained using the sample trimmed at the 95% percentile of total time spent playing a task in Tables 4, and 5, respectively.

Table A.13: Exercise A: data trimmed at the 99% percentile.

Task	N	Profile 1	Profile 2	Profile 3	Profile 4	Profile 5	Profile 6
2.2.2	1,638	69.44%	93.91%	96.3%	99.36%	97.07%	100%
			(7.96)	(1.3)	(2.48)	(-2.05)	(2.87)
2.2.3	1,729	72.52%	92.44%	95.43%	99.28%	97.25%	99.67%
			(6.71)	(1.54)	(3.05)	(-1.87)	(2.38)
2.3.2	1,630	65.4%	92.81%	91.79%	100%	97.89%	99.65%
			(8.65)	(-0.46)	(5)	(-2.47)	(1.9)
2.3.3	1,637	64.24%	95.29%	97.75%	100%	98.98%	99.64%
			(10.06)	(1.61)	(2.47)	(-1.74)	(0.95)
3.2.2	1,666	56.25%	88%	96.31%	99.37%	97.13%	99.64%
			(8.74)	(3.82)	(2.6)	(-2.05)	(2.36)
3.3.2	1,647	61.46%	84.01%	95.75%	99.68%	93.92%	98.55%
			(6.3)	(4.78)	(3.16)	(-3.82)	(2.82)
3.2.3	1,628	60.78%	85.61%	96.03%	99.23%	98.23%	100%
			(6.91)	(4.36)	(2.57)	(-1.05)	(2.25)
3.3.3	1,638	54.8%	84.35%	96.1%	99.34%	97.66%	99.64%
			(7.92)	(4.65)	(2.6)	(-1.69)	(2.1)
4.2.2	1,717	51.93%	85.3%	95.44%	100%	96.38%	99.31%
			(9.33)	(4.3)	(3.68)	(-3.37)	(2.48)
2.2.2.2	1,660	7.72%	27.9%	74.48%	94.31%	92.31%	99.65%
			(6.48)	(12.46)	(6.82)	(-0.97)	(4.54)
2.2.2.3	1,610	27.14%	61.97%	89.86%	98.25%	95.65%	99.28%
			(8.87)	(8.17)	(4.24)	(-1.78)	(2.72)
2.2.3.2	1,674	26.13%	52.07%	86.46%	97.97%	96.86%	100%
			(6.61)	(9.64)	(5.28)	(-0.84)	(3.04)
2.3.2.2	1,606	4.14%	7.53%	50.7%	80%	93.73%	99.26%
			(1.7)	(12.86)	(7.72)	(4.95)	(3.63)
3.2.2.2	1,575	15.07%	32.73%	83.51%	95.02%	93.55%	97.82%
			(4.94)	(14.09)	(4.42)	(-0.74)	(2.49)
2.2.2.4	1,602	46.04%	68.18%	91.27%	95.11%	95.37%	98.55%
			(5.37)	(7.12)	(1.8)	(0.15)	(2.19)
2.2.4.2	1,673	15.9%	43.16%	80.07%	97.95%	95.07%	99.66%
			(7.45)	(9.88)	(7.3)	(-1.88)	(3.45)
2.4.2.2	1,641	35%	67.25%	89.56%	94.04%	95.71%	99.3%
			(8.08)	(6.74)	(1.97)	(0.9)	(2.73)
4.2.2.2	1,614	13.98%	41.22%	77.66%	93.54%	96.1%	98.91%
			(7.54)	(9.38)	(5.47)	(1.39)	(2.14)
2.2.2.2.2	1,545	18.56%	58.09%	67.77%	93.49%	91.14%	100%
			(10.3)	(2.35)	(7.99)	(-1.01)	(5.12)
3.2.2.2.2	1,550	14.76%	44.74%	65.93%	88.35%	92.94%	97.77%
			(8.01)	(5.05)	(6.43)	(1.82)	(2.68)
4.2.2.2.2	1,566	3.3%	20.59%	29.04%	56.09%	83.15%	92.99%
			(6.44)	(2.29)	(6.61)	(7.16)	(3.58)
2.2.2.2.2.2	1,580	16.37%	25.75%	32.36%	43.12%	79.63%	84.12%
			(2.7)	(1.7)	(2.61)	(9.44)	(1.36)

Notes. The table shows the probability P_i of Profile i winning a task. The values in parentheses correspond to the p-value of testing the null hypothesis $H_0 : P_{i-1} \geq P_i$ against its alternative $H_1 : P_{i-1} < P_i$. When $P_i = P_{i-1} = 100\%$, we report NA.

Table A.14: Exercise B: data trimmed at the 99% percentile.

k	N	Seq	$Complex$	$Profile_{i,k}$
1	30,550	0.079	-0.007	0.11
		(0.00)	(0.00)	(0.00)
2	24,781	0.052	-0.007	0.137
		(0.00)	(0.00)	(0.00)
3	20,694	0.047	-0.007	0.095
		(0.00)	(0.00)	(0.00)
4	18,051	0.048	-0.007	0.113
		(0.00)	(0.00)	(0.00)
5	15,744	0.041	-0.007	0.109
		(0.00)	(0.00)	(0.00)
6	13,886	0.045	-0.007	0.077
		(0.00)	(0.00)	(0.00)
7	12,290	0.033	-0.008	0.108
		(0.00)	(0.00)	(0.00)
8	10,913	0.033	-0.007	0.111
		(0.00)	(0.00)	(0.00)
9	9,683	0.018	-0.007	0.11
		(0.06)	(0.00)	(0.00)
10	8,575	0.012	-0.008	0.133
		(0.31)	(0.00)	(0.00)
11	7,594	0.027	-0.008	0.168
		(0.03)	(0.00)	(0.00)
12	6,678	0.018	-0.008	0.173
		(0.24)	(0.00)	(0.00)
13	5,831	0.034	-0.008	0.152
		(0.08)	(0.00)	(0.00)
14	5,034	0.026	-0.008	0.176
		(0.28)	(0.00)	(0.00)
15	4,288	0.005	-0.007	0.193
		(0.87)	(0.00)	(0.00)
16	3,599	-0.008	-0.007	0.182
		(0.84)	(0.00)	(0.00)
17	2,934	-0.046	-0.008	0.288
		(0.35)	(0.00)	(0.00)
18	2,308	0.084	-0.007	0.23
		(0.23)	(0.00)	(0.00)
19	1,695	0.032	-0.008	0.281
		(0.77)	(0.00)	(0.00)
20	1,111	-0.472	-0.006	0.283
		(0.06)	(0.00)	(0.00)
21	0,538	–	-0.004	0.225
			(0.00)	(0.00)

Notes. The table shows the results from estimating a logit model. The dependent variable captures whether the subject backward inducted ($Y_i = 1$) or did not backward induct ($Y_i = 0$) in a given task. The independent variables are Seq (order in the sequence in which a task appeared for the subject), $Complex$ (measure of task complexity), and $Profile_{i,k}$ (profile of subject i calculated in the tasks that appeared in the order $Seq - k$ of the sequence). The regression includes an intercept. The parentheses contain p-values calculated using heteroskedastic robust standard errors.

Table A.15: Exercise C: data trimmed at the 99% percentile.

g	N	Seq	$Complex$	$Profile_{i,g}$
1	8,637	0.017	-0.008	0.337
		(0.14)	(0.00)	(0.34)
2	8,514	0.016	-0.008	0.44
		(0.15)	(0.00)	(0.44)
3	8,373	0.011	-0.008	0.542
		(0.35)	(0.00)	(0.54)
4	8,259	0.008	-0.008	0.571
		(0.47)	(0.00)	(0.57)
5	8,186	0.011	-0.008	0.574
		(0.34)	(0.00)	(0.57)
6	8,111	0.011	-0.008	0.609
		(0.35)	(0.00)	(0.61)
7	8,022	0.01	-0.008	0.659
		(0.39)	(0.00)	(0.66)
8	7,925	0.012	-0.008	0.666
		(0.31)	(0.00)	(0.67)
9	7,838	0.011	-0.008	0.69
		(0.37)	(0.00)	(0.69)

Notes. The table shows the results from estimating a logit model. The dependent variable captures whether the subject backward inducted ($Y_i = 1$) or did not backward induct ($Y_i = 0$) in a given task. The independent variables are Seq (order in the sequence in which a task appeared for the subject), $Complex$ (measure of task complexity), and $Profile_{i,g}$ (profile of subject i calculated using the first g tasks the subject played). The regression includes an intercept. The parentheses contain p-values calculated using heteroskedastic robust standard errors.

B Appendix: one-dimensional types

In this appendix we provide further empirical evidence that a lexicographic ranking based on $RRT1$ and TT is needed to rank subjects according to their probability of correctly backward inducting.

For this purpose, we investigate the predictive power of the two dimensions ($RRT1$ and TT) used to construct the profiles separately and in tandem. More precisely, the following three logit models are estimated for each of the 22 tasks.

$$\text{Model A1. } Logit(Y) = \alpha + \beta_1 RRT1 + \beta_3 Seq \tag{5}$$

$$\text{Model A2. } Logit(Y) = \alpha + \beta_2 TT + \beta_3 Seq \tag{6}$$

$$\text{Model A3. } Logit(Y) = \alpha + \beta_1 RRT1 + \beta_2 TT + \beta_3 Seq \tag{7}$$

Y is the dependent variable in the regression and captures whether the subject backward inducted ($Y_i = 1$) or did not backward induct ($Y_i = 0$), α is the intercept, and $RRT1$, TT, and Seq are the independent variables. The independent variable Seq is an integer number which corresponds to the order in which a task appeared in the subject's sequence of tasks. Results are presented in Table B.1, where p-values of the estimated parameters are in parenthesis.

Table B.1: Results from regressions.

		Model A1		Model A2		Model A3		
Task	N	$RRT1$	Seq	TT	Seq	$RRT1$	TT	Seq
2.2.2	1,638	11.129	0.143	-0.085	0.128	10.063	-0.068	0.139
		(0.00)	(0.00)	(0.00)	(0.00)	(0.00)	(0.00)	(0.00)
2.2.3	1,729	12.779	0.115	-0.066	0.063	12.285	-0.058	0.119
		(0.00)	(0.00)	(0.00)	(0.00)	(0.00)	(0.01)	(0.00)
2.3.2	1,630	11.280	0.063	-0.083	0.064	10.206	-0.071	0.056
		(0.00)	(0.00)	(0.00)	(0.00)	(0.00)	(0.00)	(0.00)
2.3.3	1,637	12.856	0.110	-0.058	0.122	12.585	-0.063	0.116
		(0.00)	(0.00)	(0.00)	(0.00)	(0.00)	(0.00)	(0.00)
3.2.2	1,666	13.238	0.092	-0.065	0.076	12.536	-0.052	0.087
		(0.00)	(0.00)	(0.00)	(0.00)	(0.00)	(0.00)	(0.00)
3.3.2	1,647	10.728	0.077	-0.051	0.076	10.370	-0.046	0.079
		(0.00)	(0.00)	(0.00)	(0.00)	(0.00)	(0.00)	(0.00)
3.2.3	1,628	12.522	0.056	-0.054	0.051	11.935	-0.038	0.057
		(0.00)	(0.01)	(0.00)	(0.00)	(0.00)	(0.04)	(0.01)
3.3.3	1,638	13.188	0.085	-0.038	0.089	13.289	-0.044	0.083
		(0.00)	(0.00)	(0.00)	(0.00)	(0.00)	(0.00)	(0.00)
4.2.2	1,717	13.483	0.108	-0.050	0.093	13.080	-0.031	0.111
		(0.00)	(0.00)	(0.00)	(0.00)	(0.00)	(0.02)	(0.00)
2.2.2.2	1,660	14.045	0.049	-0.019	0.063	15.339	-0.048	0.044
		(0.00)	(0.00)	(0.00)	(0.00)	(0.00)	(0.00)	(0.00)
2.2.2.3	1,610	11.764	0.079	-0.008	0.087	13.430	-0.041	0.076
		(0.00)	(0.00)	(0.03)	(0.00)	(0.00)	(0.00)	(0.00)
2.2.3.2	1,674	13.047	0.052	-0.003	0.085	13.890	-0.029	0.048
		(0.00)	(0.00)	(0.38)	(0.00)	(0.00)	(0.00)	(0.00)
2.3.2.2	1,606	16.693	0.040	-0.001	0.068	19.621	-0.045	0.032
		(0.00)	(0.00)	(0.61)	(0.00)	(0.00)	(0.00)	(0.01)
3.2.2.2	1,575	15.589	0.050	0.006	0.076	18.785	-0.037	0.049
		(0.00)	(0.00)	(0.03)	(0.00)	(0.00)	(0.00)	(0.00)
2.2.2.4	1,602	10.999	0.082	0.008	0.092	11.615	-0.019	0.080
		(0.00)	(0.00)	(0.07)	(0.00)	(0.00)	(0.01)	(0.00)
2.2.4.2	1,673	12.814	0.021	-0.010	0.056	14.906	-0.041	0.021
		(0.00)	(0.06)	(0.00)	(0.00)	(0.00)	(0.00)	(0.07)
2.4.2.2	1,641	11.416	0.048	0.007	0.076	13.240	-0.032	0.046
		(0.00)	(0.00)	(0.07)	(0.00)	(0.00)	(0.00)	(0.00)
4.2.2.2	1,614	15.598	0.008	0.004	0.049	19.239	-0.038	0.005
		(0.00)	(0.46)	(0.14)	(0.00)	(0.00)	(0.00)	(0.71)
2.2.2.2.2	1,545	7.715	0.017	-0.002	0.051	10.587	-0.030	0.016
		(0.00)	(0.11)	(0.18)	(0.00)	(0.00)	(0.00)	(0.19)
3.2.2.2.2	1,550	7.781	0.031	0.006	0.072	9.744	-0.017	0.029
		(0.00)	(0.00)	(0.00)	(0.00)	(0.00)	(0.00)	(0.01)
4.2.2.2.2	1,566	8.068	0.001	0.006	0.043	9.953	-0.007	0.001
		(0.00)	(0.89)	(0.00)	(0.00)	(0.00)	(0.00)	(0.90)
2.2.2.2.2.2	1,580	5.777	0.022	0.007	0.051	6.544	-0.004	0.023
		(0.00)	(0.01)	(0.00)	(0.00)	(0.00)	(0.00)	(0.01)

Notes. The table shows the results from estimating three logit models. The dependent variable in all models captures whether the subject backward inducted ($Y_i = 1$) or did not backward induct ($Y_i = 0$) in a given task. The independent variables are Seq (order in the sequence in which a task appeared for the subject), and $RRT1$ for Model A1; Seq and TT for Model A2; and Seq, $RRT1$, and TT for Model A3. The regressions include an intercept. The parentheses contain p-values calculated using heteroskedastic robust standard errors.

First, for Model A1 we observe that the estimated parameter accompanying $RRT1$ (β_1) is always positive and statistically significant. This means that a higher $RRT1$ corresponds to a higher probability of correctly backward inducting. The model also shows that the estimated parameter accompanying Seq (β_3) is always positive and in 19 out of 22 tasks it is also statistically significant. As we already showed in the paper, experience has a positive effect in the subject's probability of backward inducting.

Model A2 shows that TT by itself is not a reliable predictor of the probability of a subject backward inducting. In 15 out of 22 cases a lower TT implies a higher probability of backward inducting and in 7 cases a higher TT implies a higher probability of backward inducting. In 6 cases the estimated parameter is not statistically significant at the 5% level of confidence.

Model A3 shows that when $RRT1$ and TT are used in tandem, in all 22 tasks a higher $RRT1$ implies a higher probability of backward inducting, and a lower TT also implies a higher probability of backward inducting. In every task the estimated parameters accompanying both variables (β_1 and β_2) are highly statistically significant.

To sum up, our results show unequivocal power for $RRT1$ to predict the probability of a subject correctly backward inducting. Importantly, it also shows that TT is a reliable predictor only when controlling for $RRT1$, empirically supporting the use of a lexicographic ranking in out profiling method.

C Appendix: mandatory tutorial

Experimental instructions are provided via the mandatory tutorial. Subjects play two tutorial tasks. In the first task, subjects do not make any choices because all the moves are indicated by the tutorial. The goal is to force the subject to learn how to play the tasks and become familiar with the rules and objectives of *Blues and Reds*.

In the second task, subjects are not guided anymore and can choose whatever actions they want. However, there is a costless hint option accessible to the subjects by clicking a magnifying glass icon. This hint option is not available in the 22 tasks that generate data for this article.

Importantly, in order to progress, subjects must win the second tutorial task. They can attempt playing this task as many times as desired. In addition, once the tutorial is finished, subjects can revisit it at any time while playing the non-tutorial tasks. Recall that beyond the tutorial, each task can be played only once.

Below, we present the screenshots from the tutorial ordered chronologically.

Figure C.1: First tutorial task (screenshot 1).

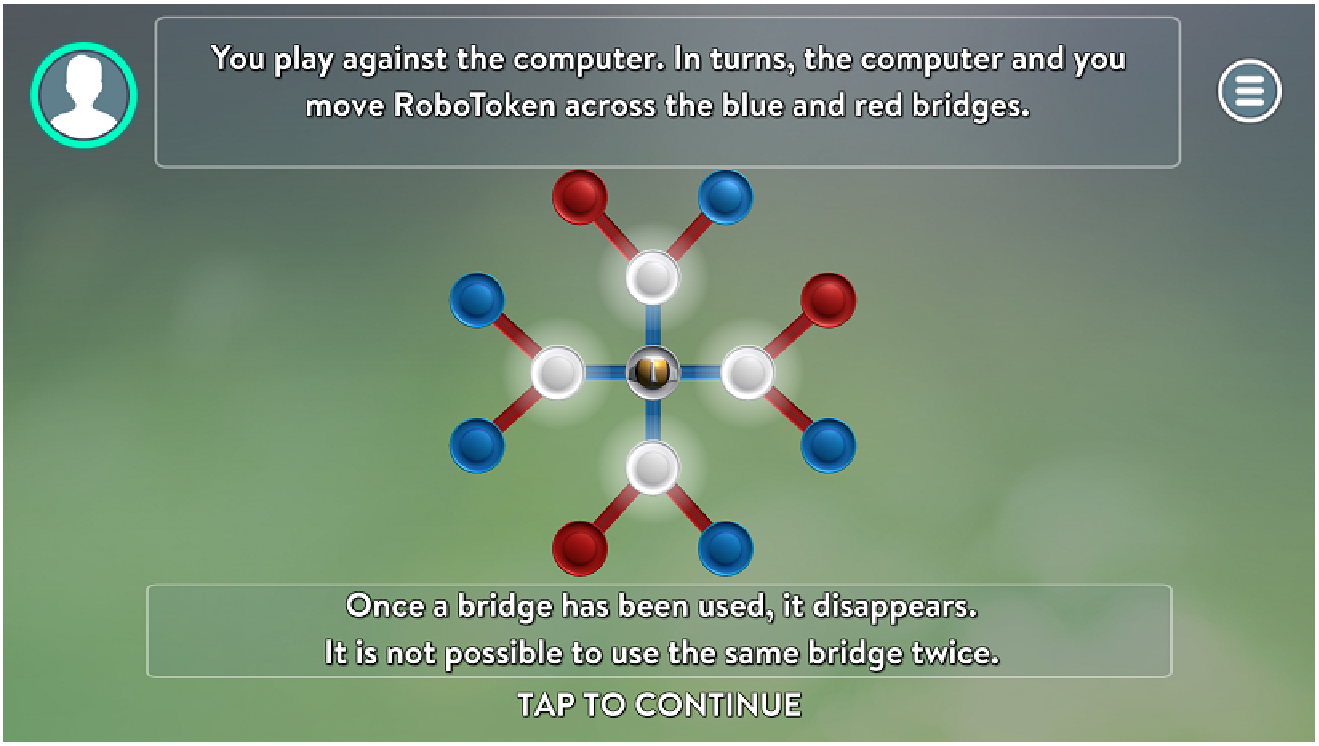

Figure C.2: First tutorial task (screenshot 2).

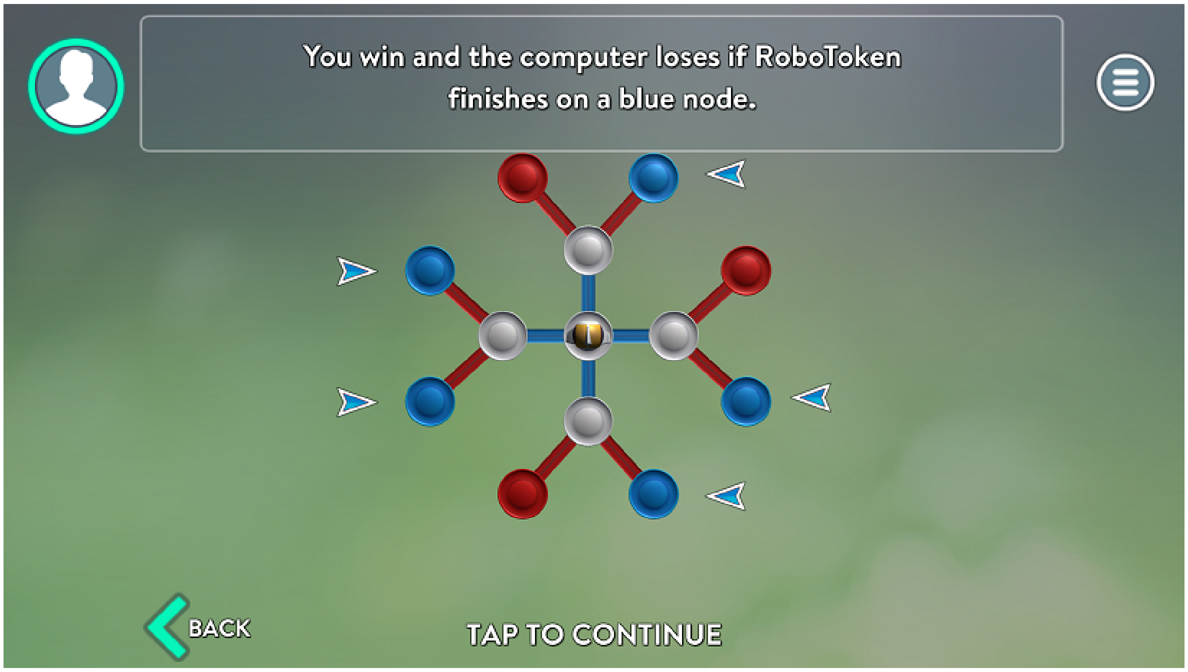

Figure C.3: First tutorial task (screenshot 3).

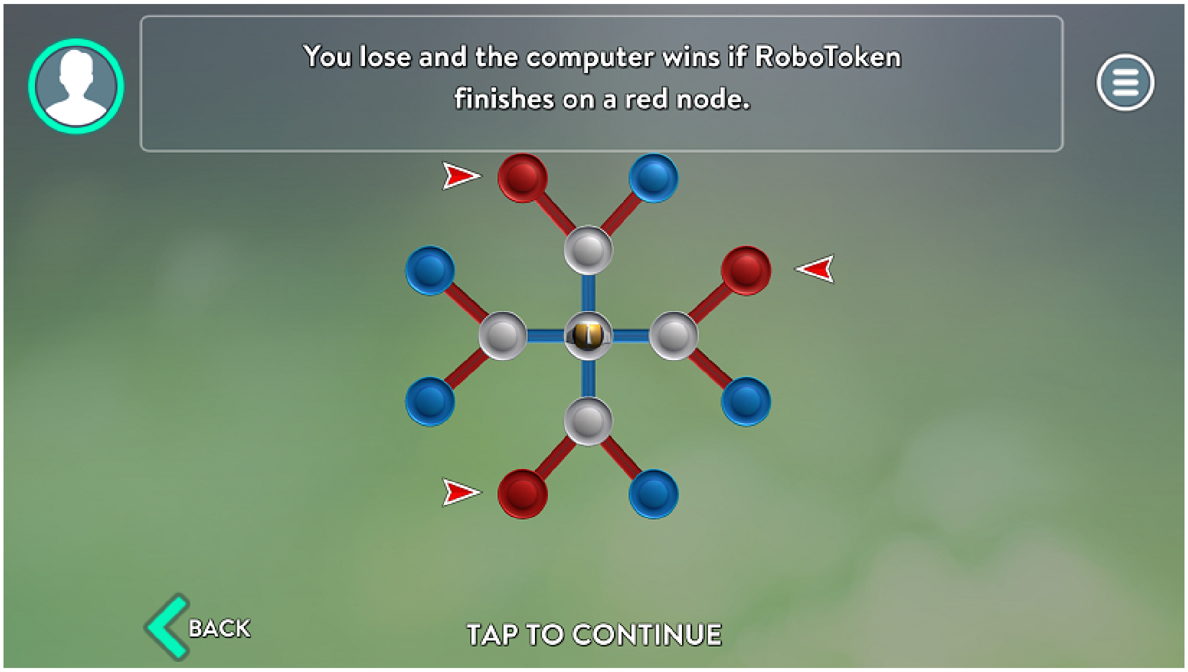

Figure C.4: First tutorial task (screenshot 4).

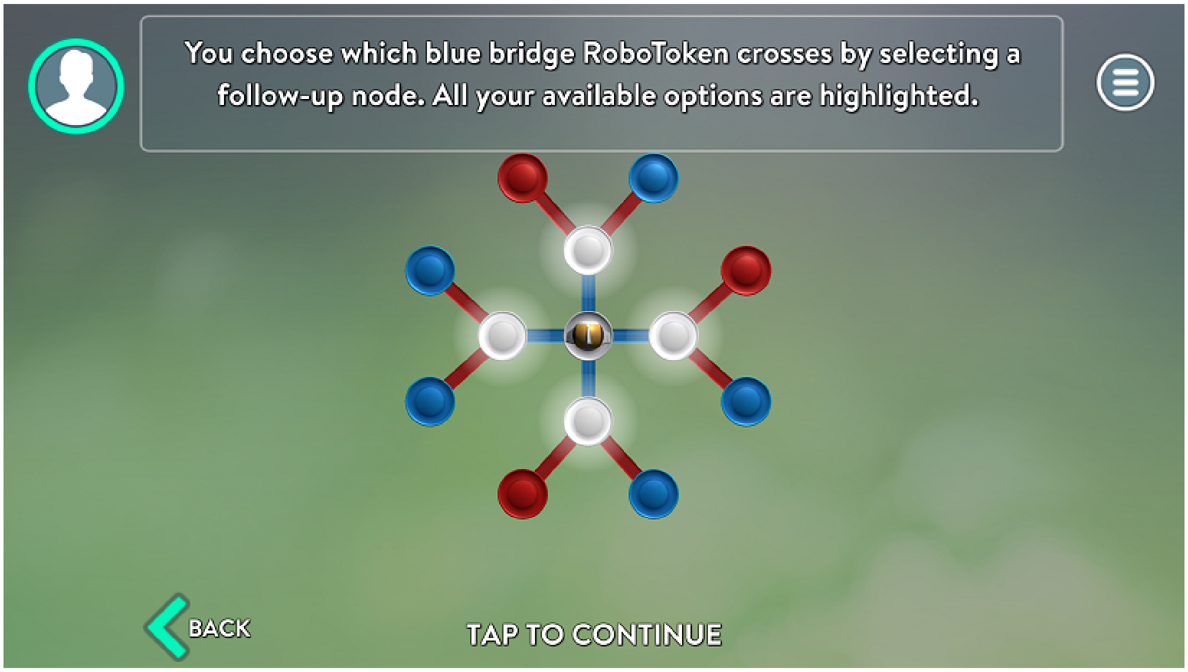

Figure C.5: First tutorial task (screenshot 5).

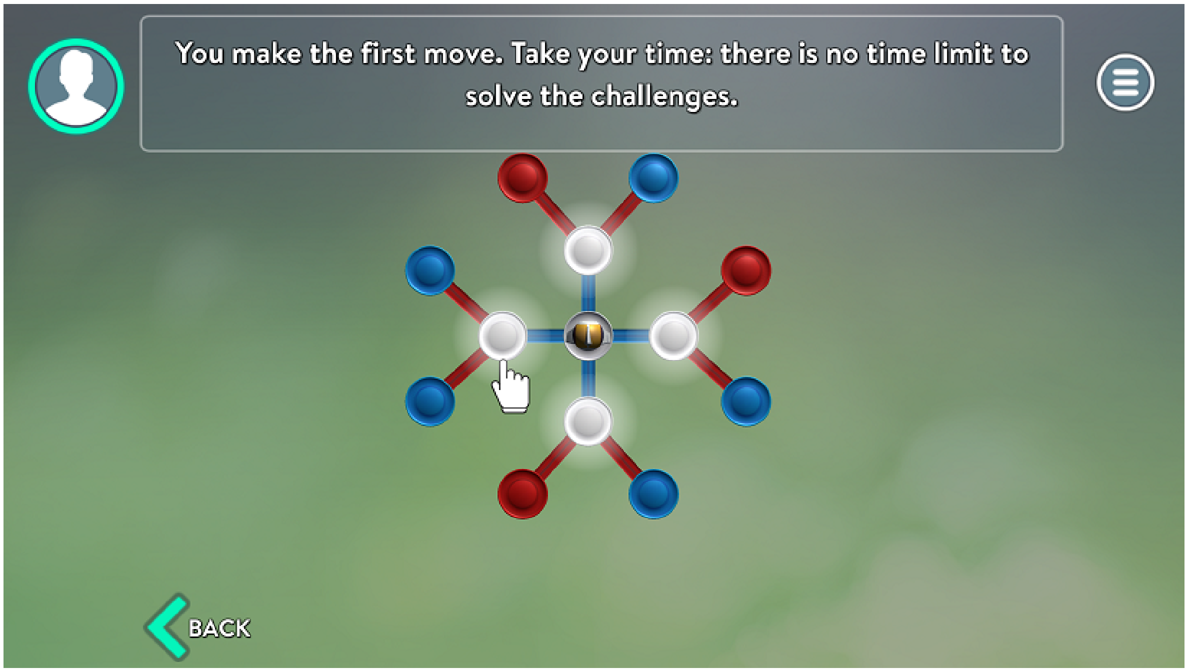

Figure C.6: First tutorial task (screenshot 6).

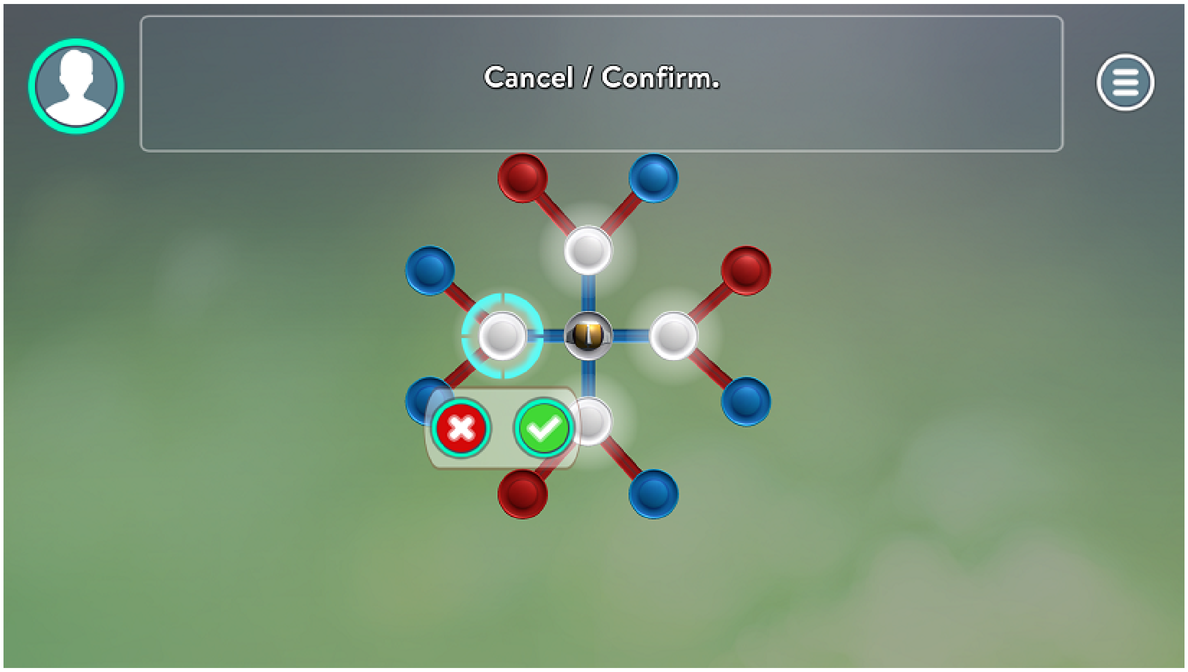

Figure C.7: First tutorial task (screenshot 7).

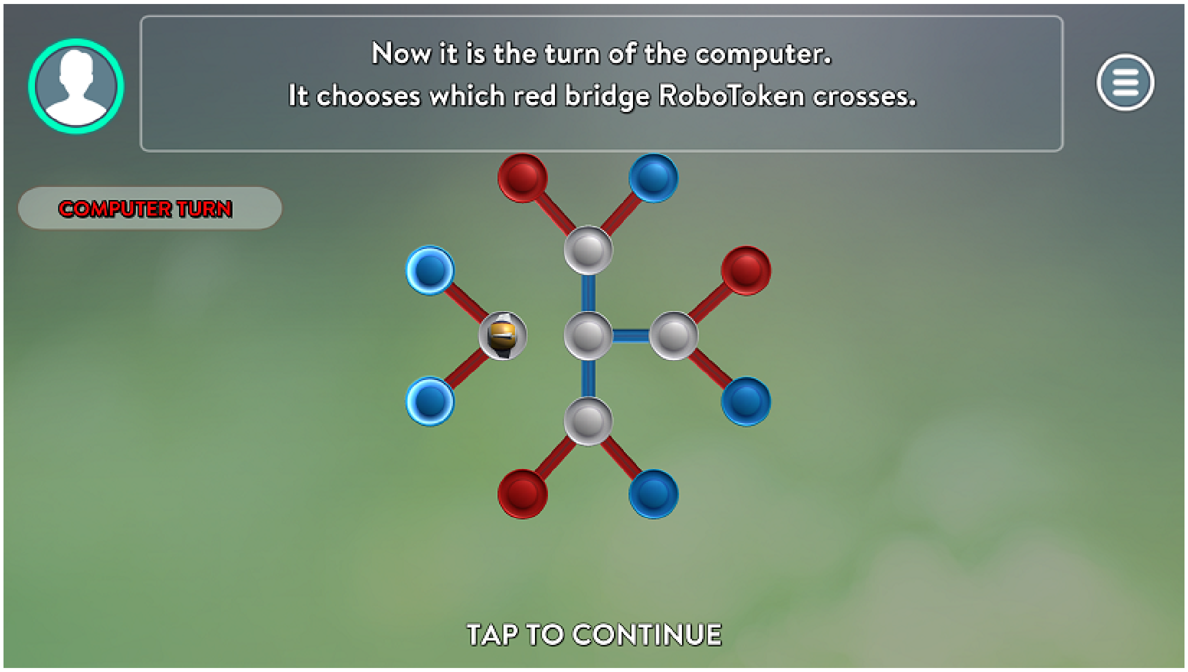

Figure C.8: Second tutorial task.

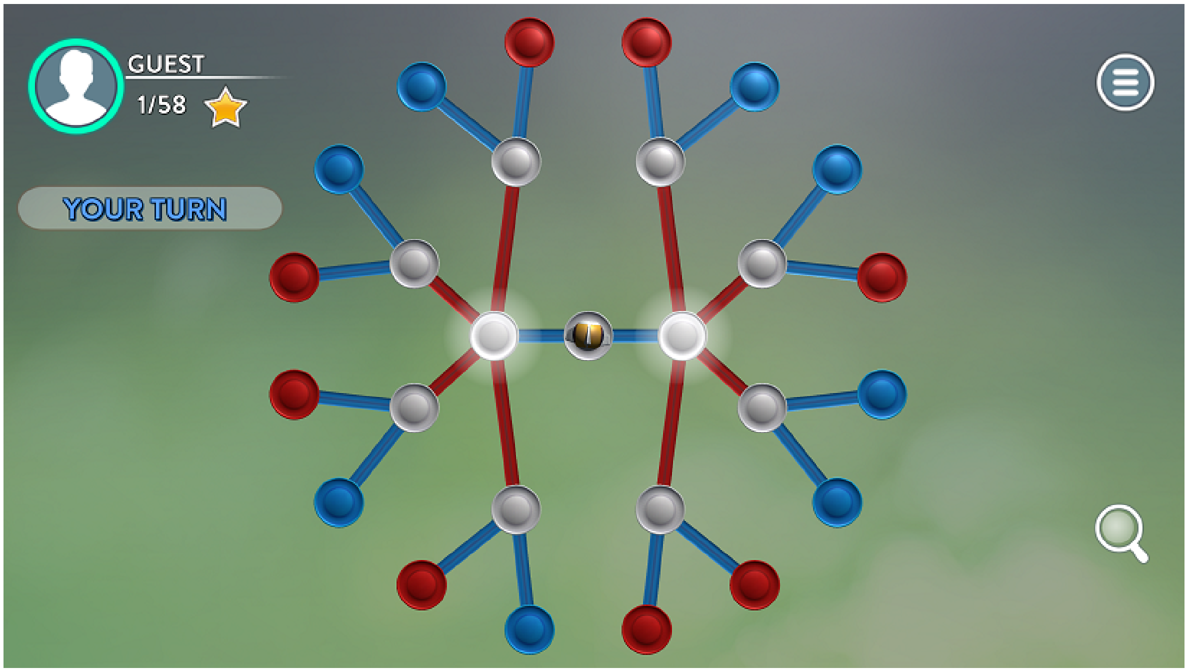

D Appendix: screenshots of tasks in *Blues and Reds*

Figure D.1: Task 2.2.2

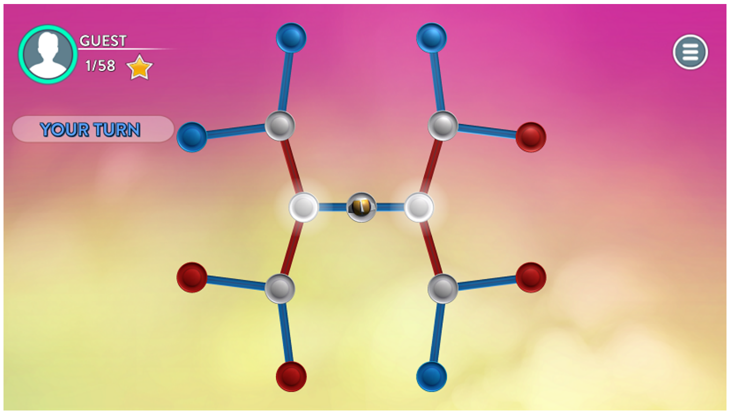

Figure D.2: Task 2.2.3

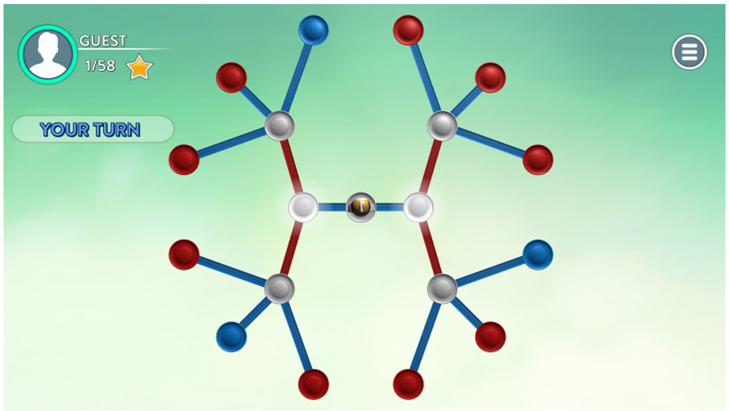

Figure D.3: Task 2.3.2

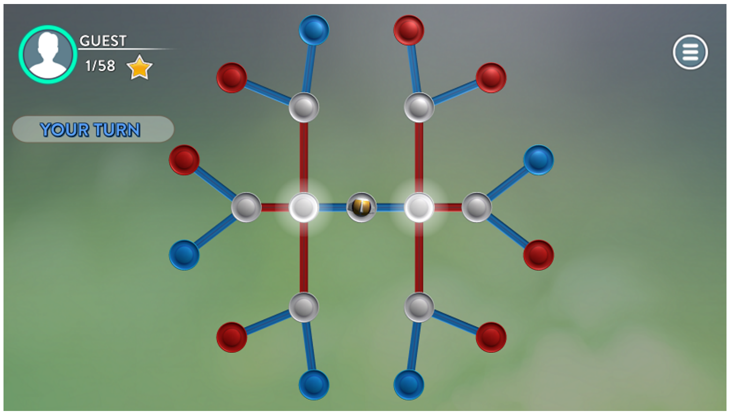

Figure D.4: Task 2.3.3

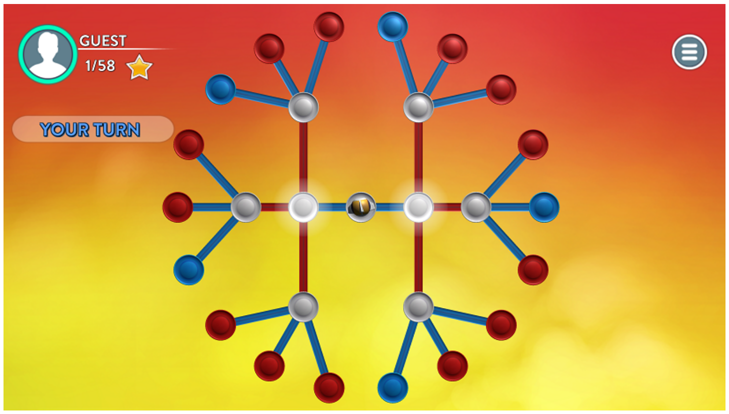

Figure D.5: Task 3.2.2

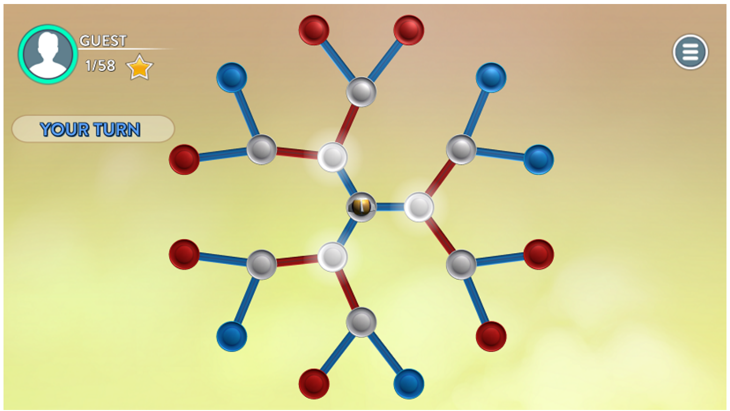

Figure D.6: Task 3.3.2

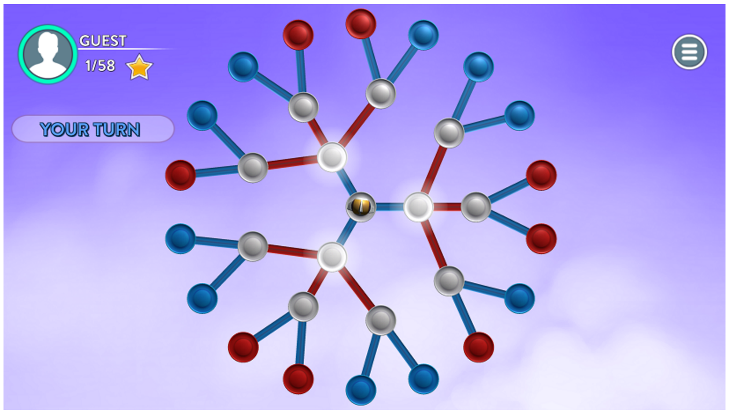

Figure D.7: Task 3.2.3

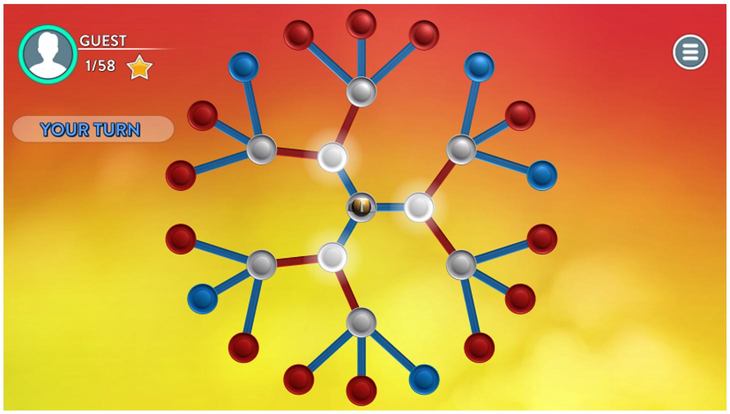

Figure D.8: Task 3.3.3

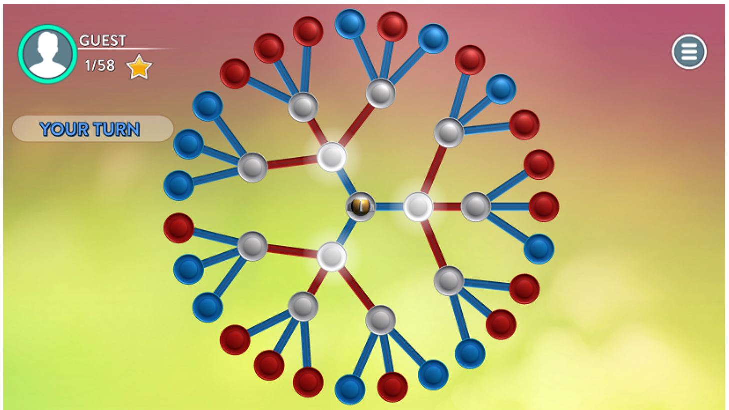

Figure D.9: Task 4.2.2

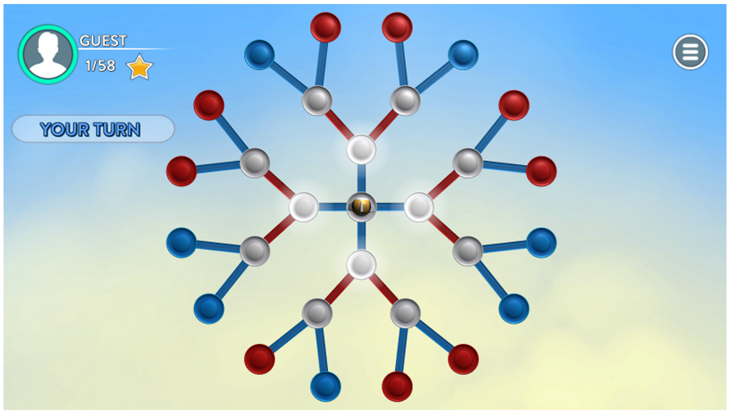

Figure D.10: Task 2.2.2.2

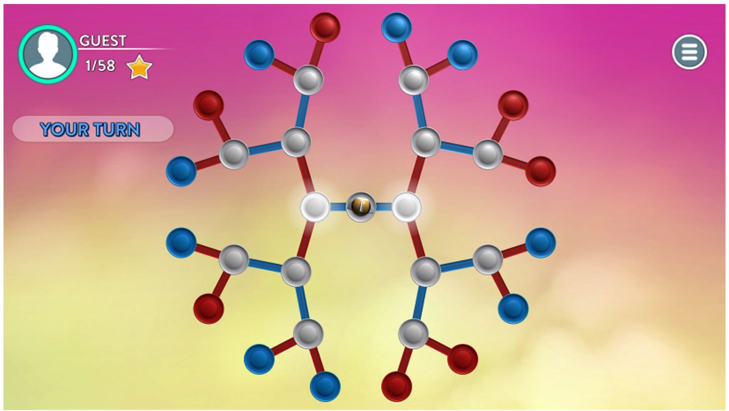

Figure D.11: Task 2.2.2.3

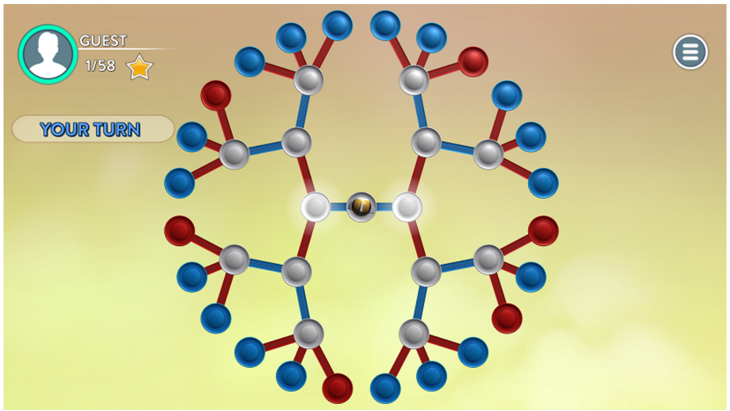

Figure D.12: Task 2.2.3.2

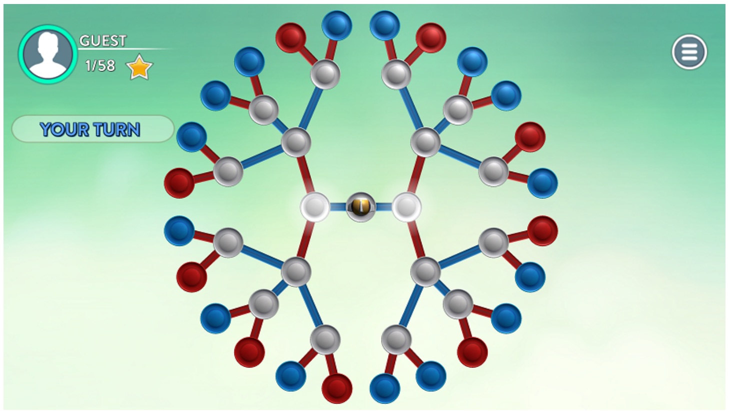

Figure D.13: Task 2.3.2.2

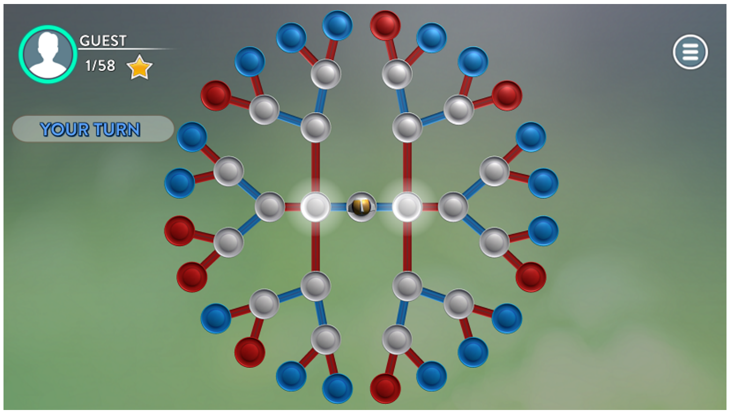

Figure D.14: Task 3.2.2.2

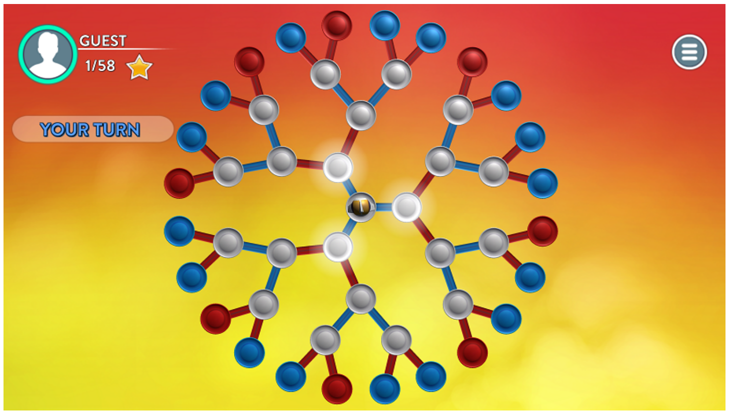

Figure D.15: Task 2.2.2.4

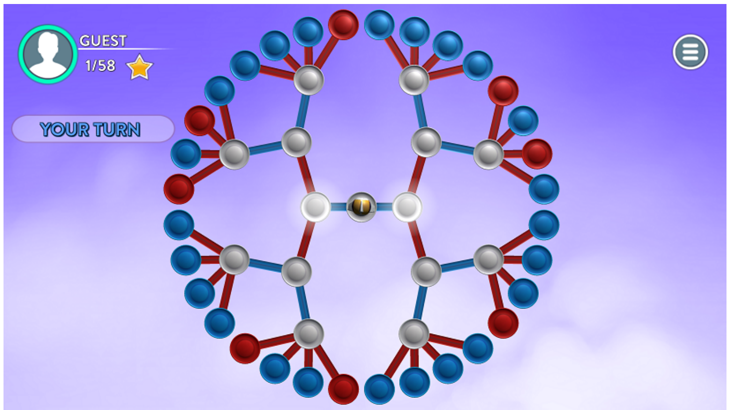

Figure D.16: Task 2.2.4.2

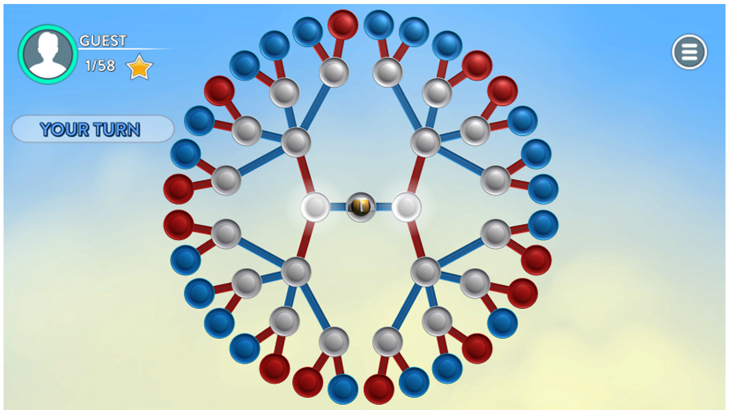

Figure D.17: Task 2.4.2.2

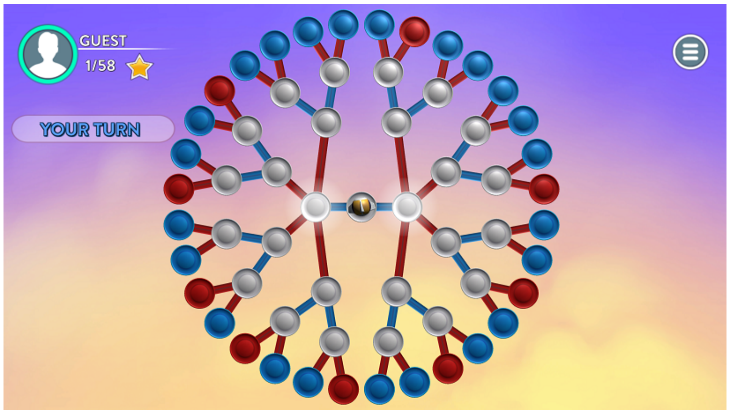

Figure D.18: Task 4.2.2.2

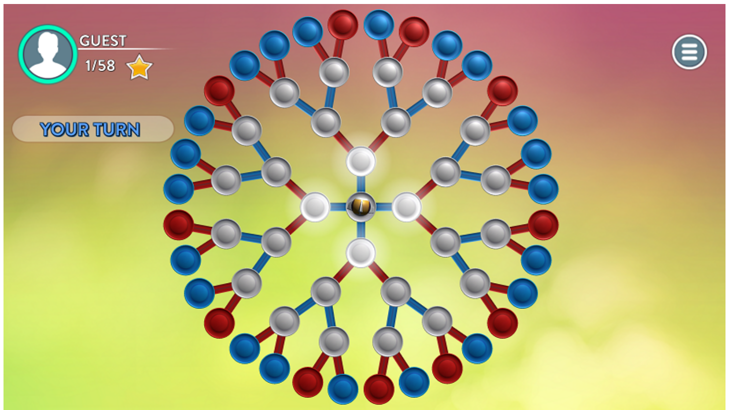

Figure D.19: Task 2.2.2.2.2

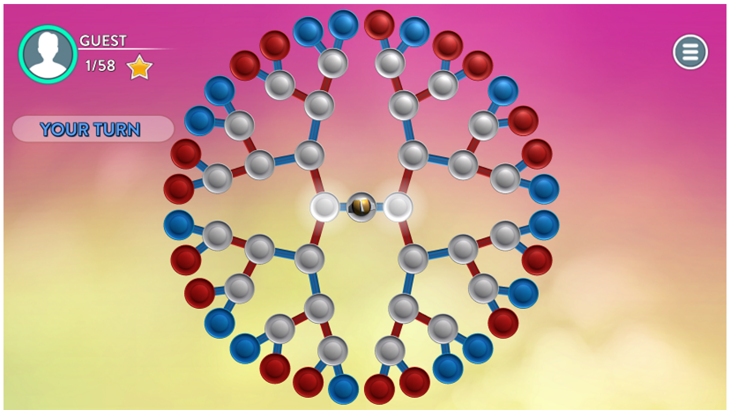

Figure D.20: Task 3.2.2.2.2

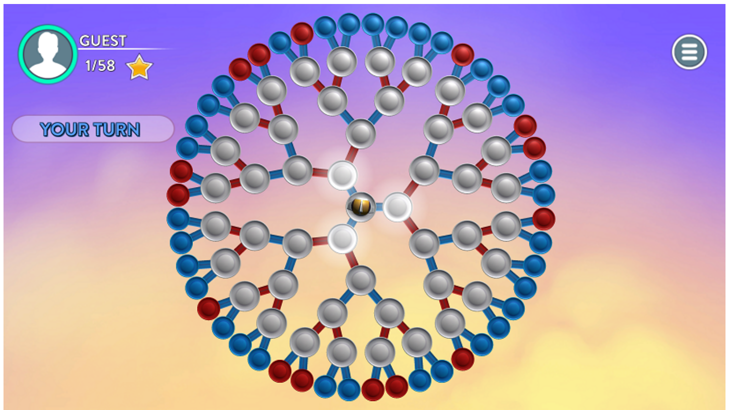

Figure D.21: Task 4.2.2.2.2

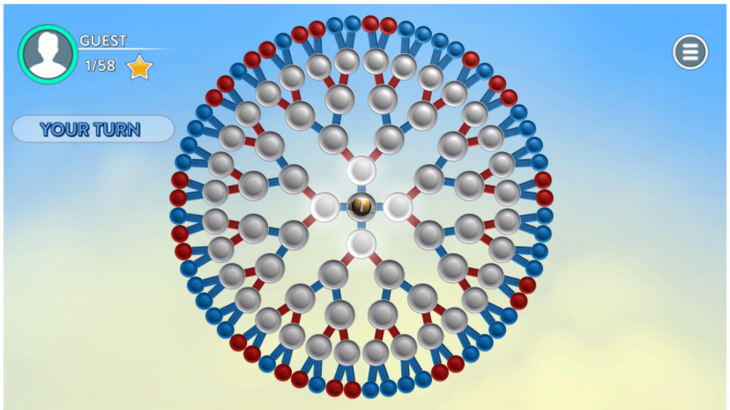

Figure D.22: Task 2.2.2.2.2.2

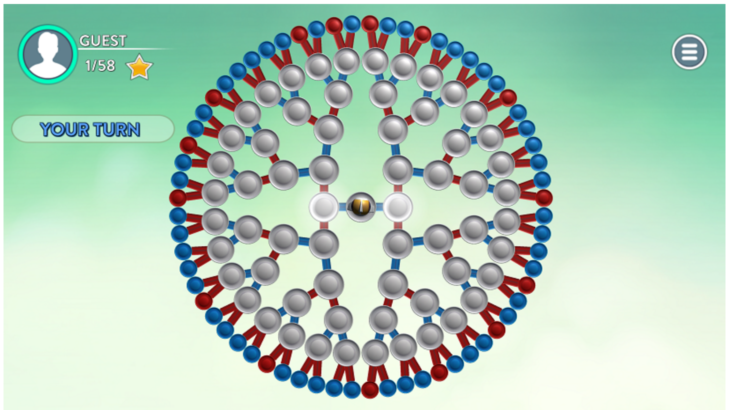

Supplement: S1 Appendix — (ZIP) [file pone.0266366.s001.zip › profiling_appendix.pdf]
