# Supplementary figures and images for: Profiling dynamic decision-makers
Source: PLoS One. 2022 Apr 14;17(4):e0266366. doi: 10.1371/journal.pone.0266366 (PMC9009624; doi:10.1371/journal.pone.0266366)

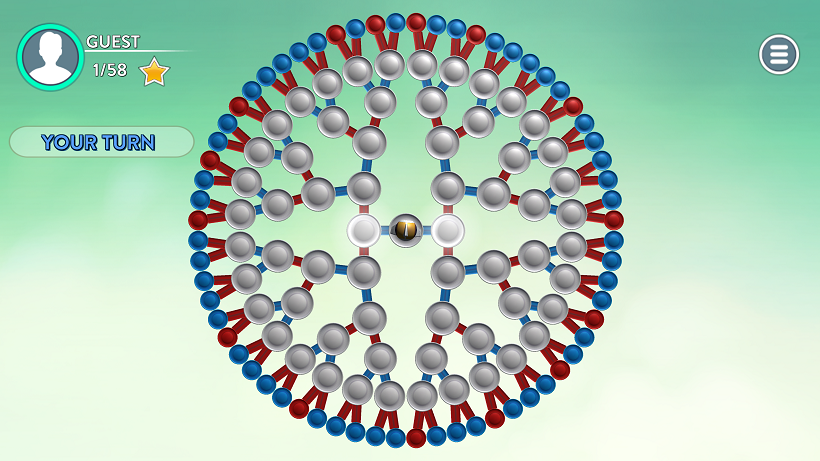

Supplement: S2 File — (ZIP) [file pone.0266366.s003.zip › 222222s.png]

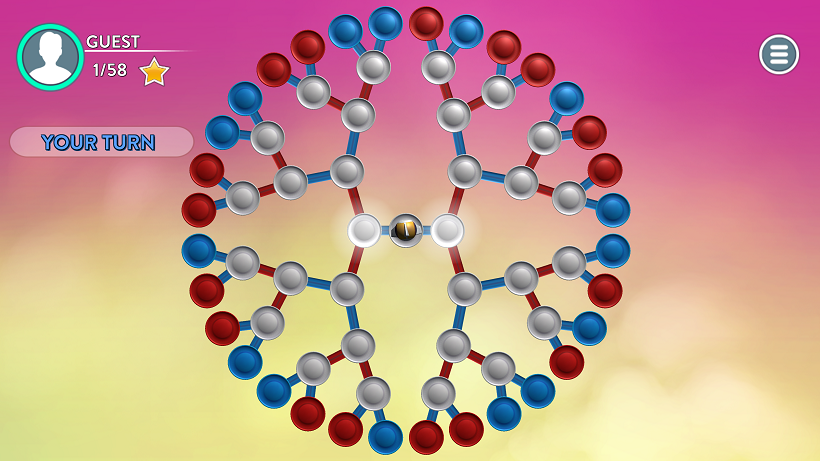

Supplement: S2 File — (ZIP) [file pone.0266366.s003.zip › 22222s.png]

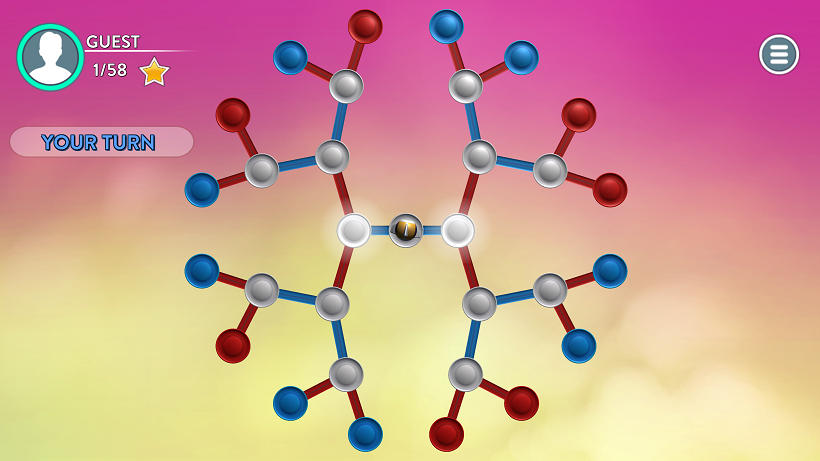

Supplement: S2 File — (ZIP) [file pone.0266366.s003.zip › 2222s.png]

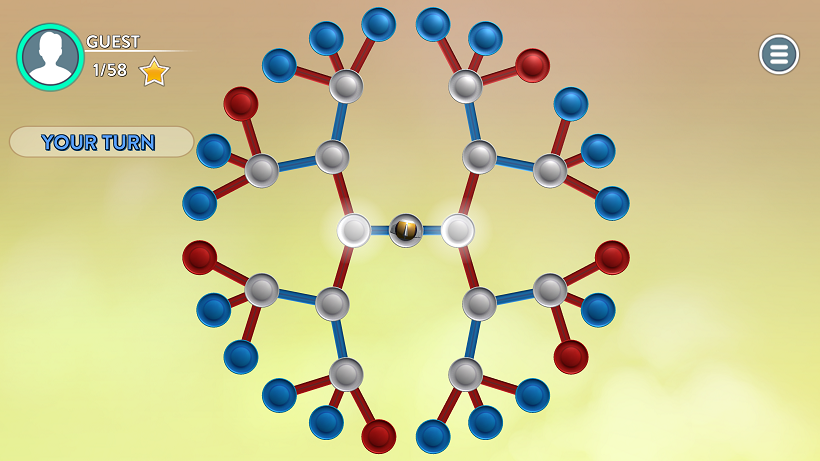

Supplement: S2 File — (ZIP) [file pone.0266366.s003.zip › 2223s.png]

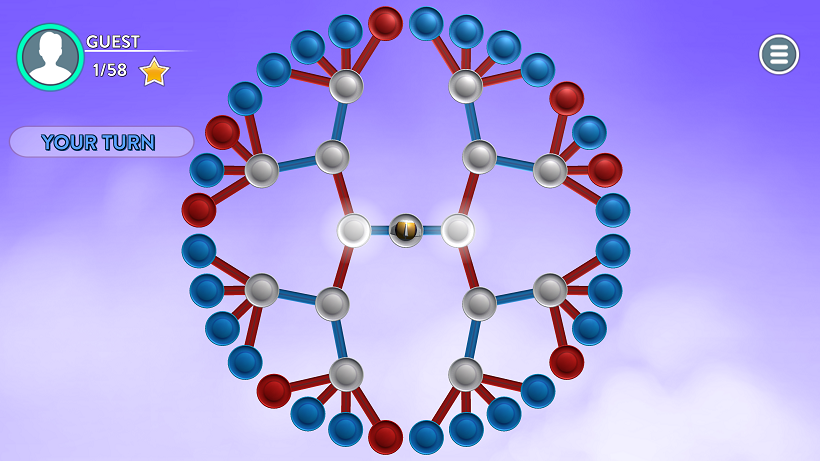

Supplement: S2 File — (ZIP) [file pone.0266366.s003.zip › 2224s.png]

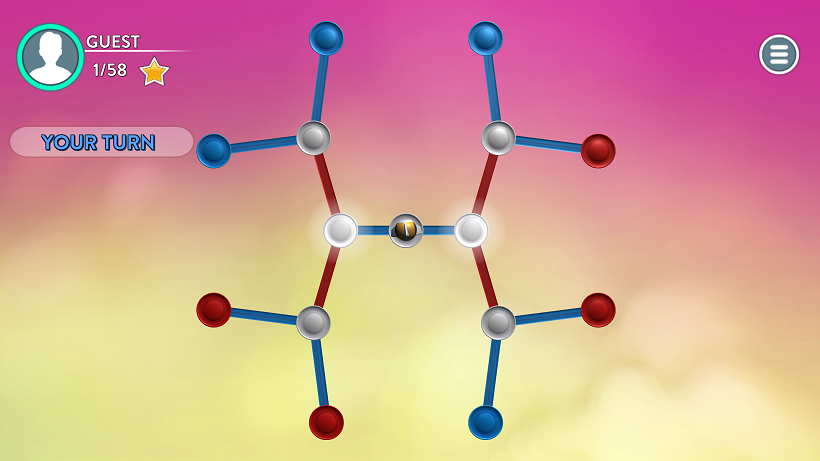

Supplement: S2 File — (ZIP) [file pone.0266366.s003.zip › 222s.png]

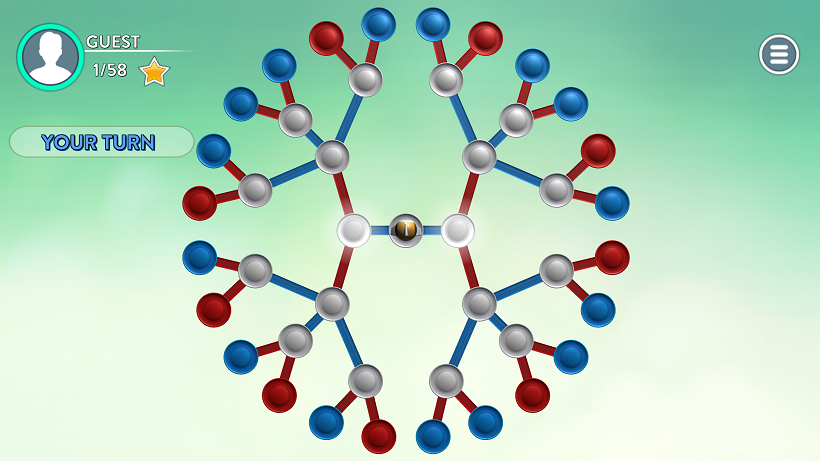

Supplement: S2 File — (ZIP) [file pone.0266366.s003.zip › 2232s.png]

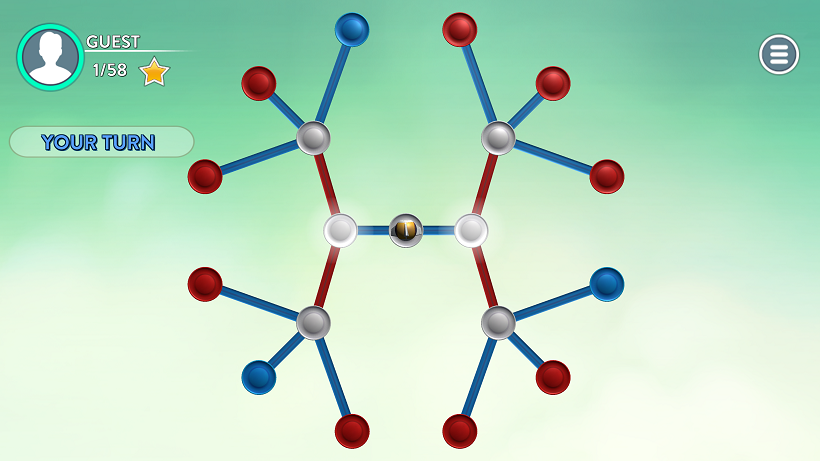

Supplement: S2 File — (ZIP) [file pone.0266366.s003.zip › 223s.png]

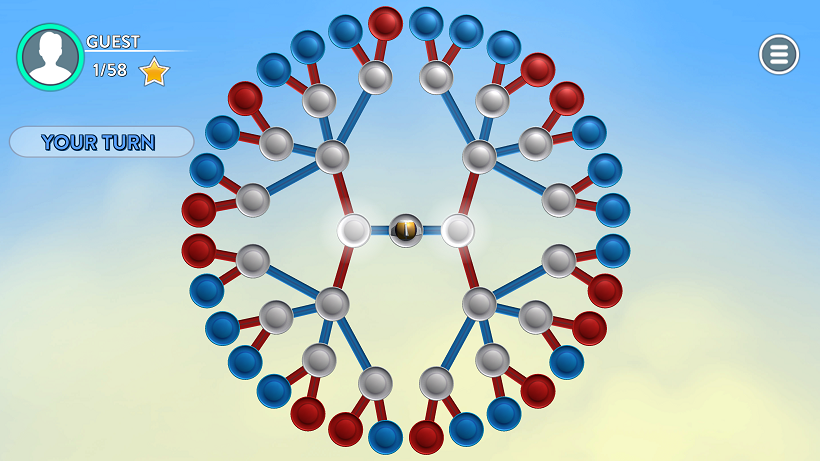

Supplement: S2 File — (ZIP) [file pone.0266366.s003.zip › 2242s.png]

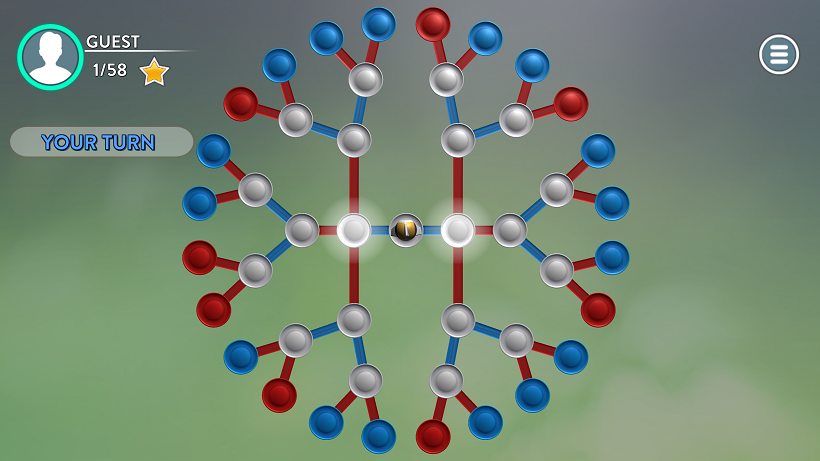

Supplement: S2 File — (ZIP) [file pone.0266366.s003.zip › 2322s.png]

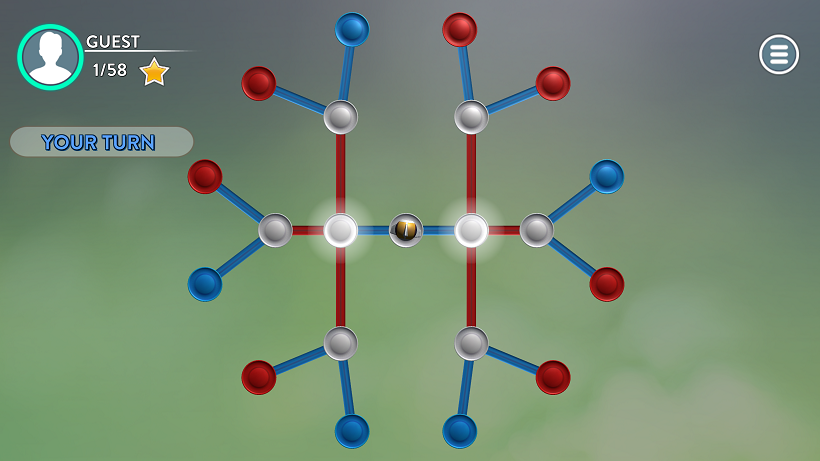

Supplement: S2 File — (ZIP) [file pone.0266366.s003.zip › 232s.png]

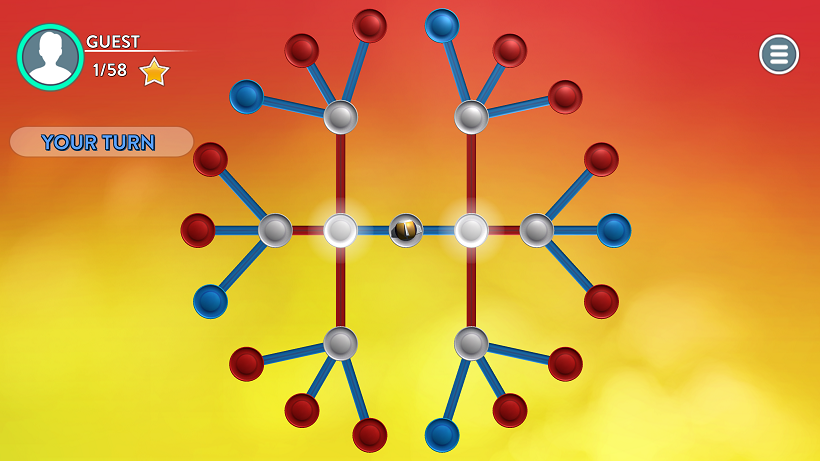

Supplement: S2 File — (ZIP) [file pone.0266366.s003.zip › 233s.png]

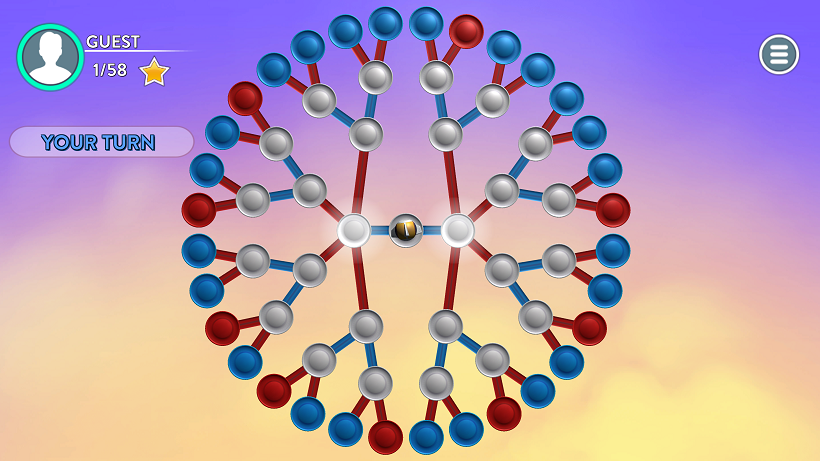

Supplement: S2 File — (ZIP) [file pone.0266366.s003.zip › 2422s.png]

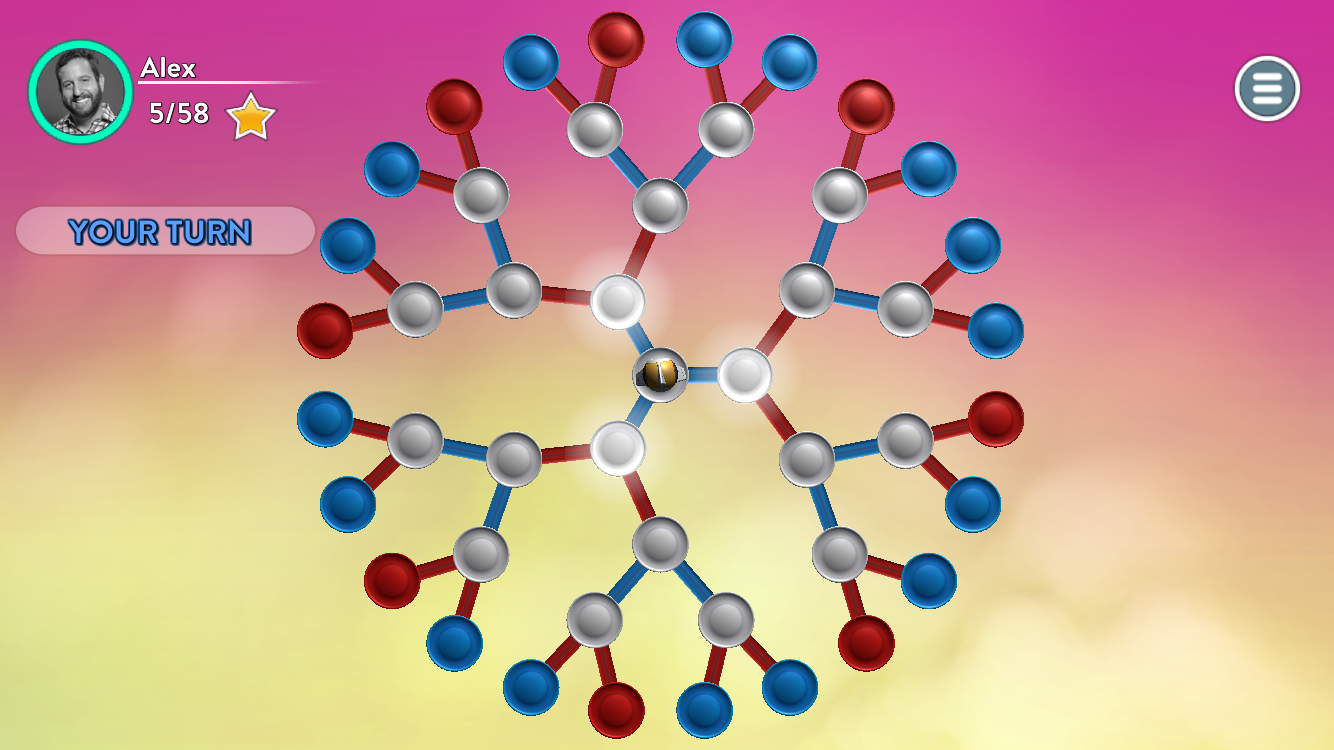

Supplement: S2 File — (ZIP) [file pone.0266366.s003.zip › 3222_tree.PNG]

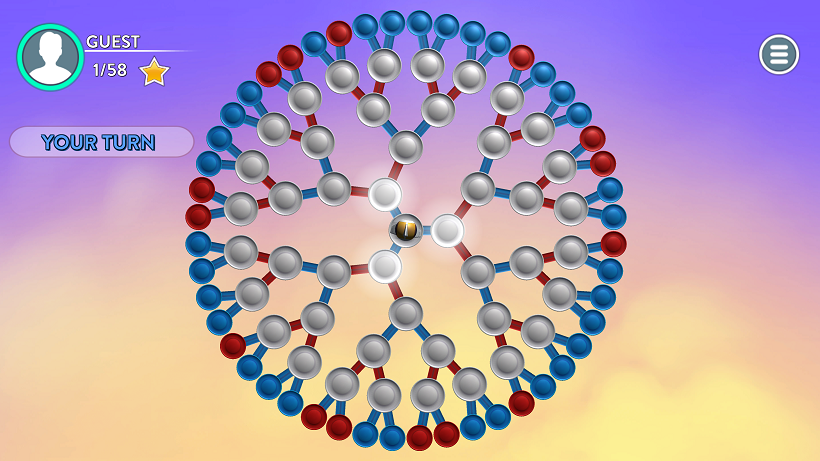

Supplement: S2 File — (ZIP) [file pone.0266366.s003.zip › 32222s.png]

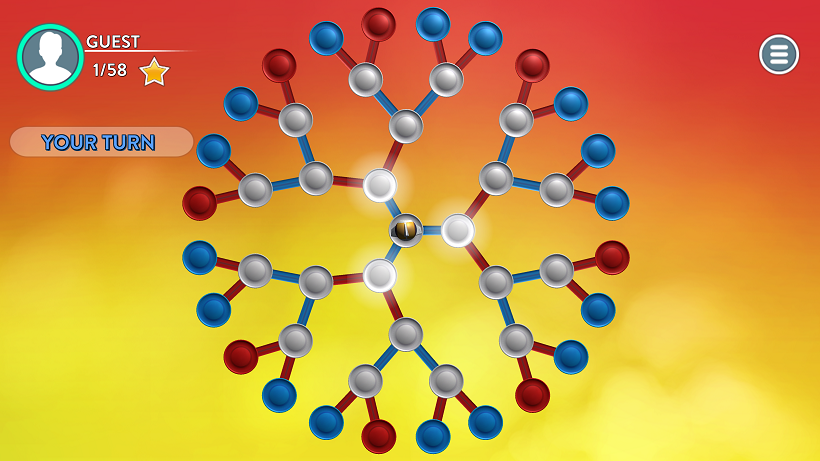

Supplement: S2 File — (ZIP) [file pone.0266366.s003.zip › 3222s.png]

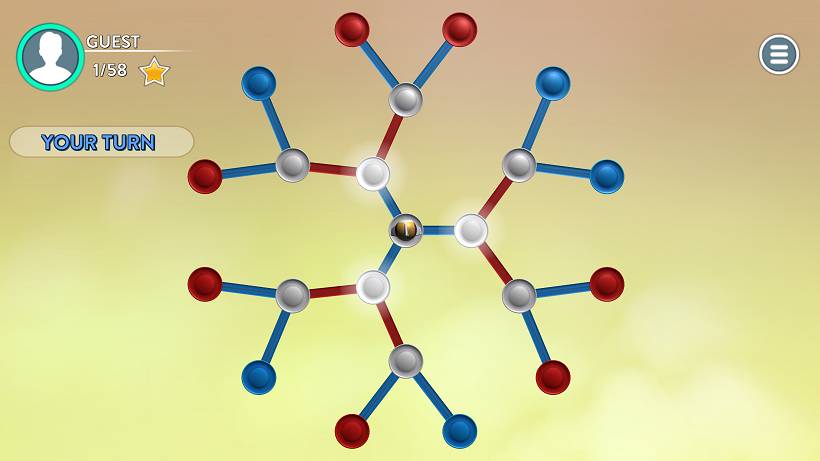

Supplement: S2 File — (ZIP) [file pone.0266366.s003.zip › 322s.png]

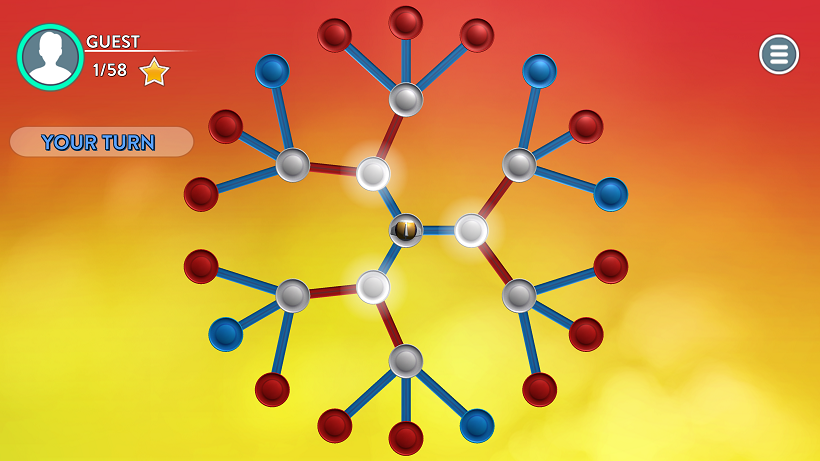

Supplement: S2 File — (ZIP) [file pone.0266366.s003.zip › 323s.png]

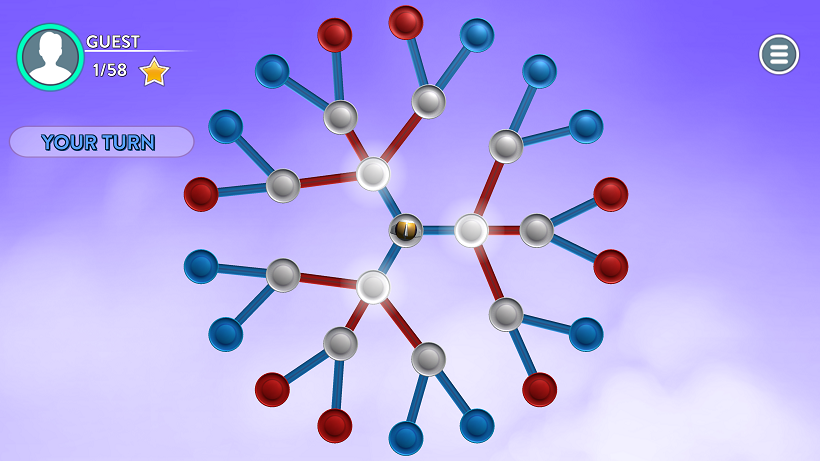

Supplement: S2 File — (ZIP) [file pone.0266366.s003.zip › 332s.png]

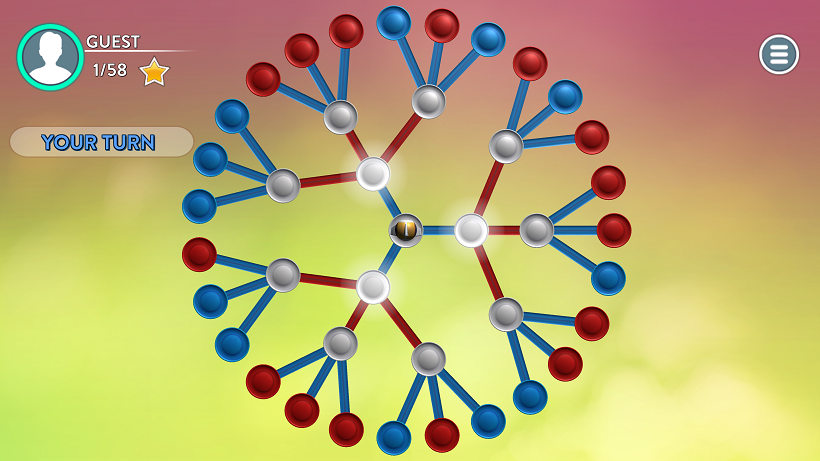

Supplement: S2 File — (ZIP) [file pone.0266366.s003.zip › 333s.png]

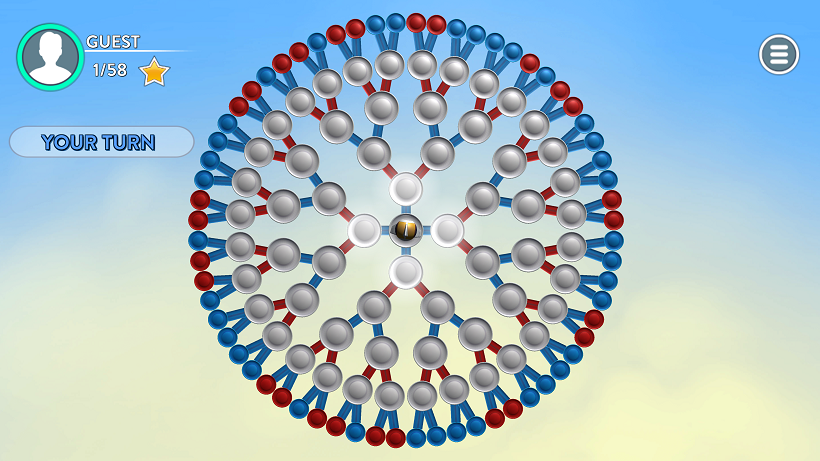

Supplement: S2 File — (ZIP) [file pone.0266366.s003.zip › 42222s.png]

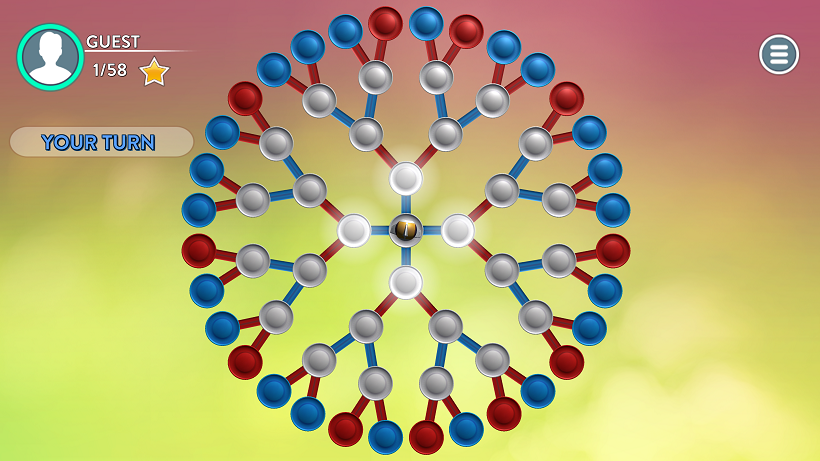

Supplement: S2 File — (ZIP) [file pone.0266366.s003.zip › 4222s.png]

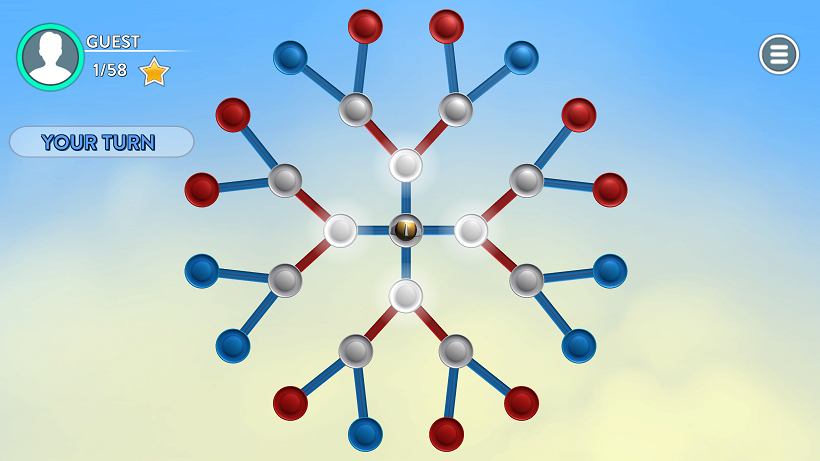

Supplement: S2 File — (ZIP) [file pone.0266366.s003.zip › 422s.png]

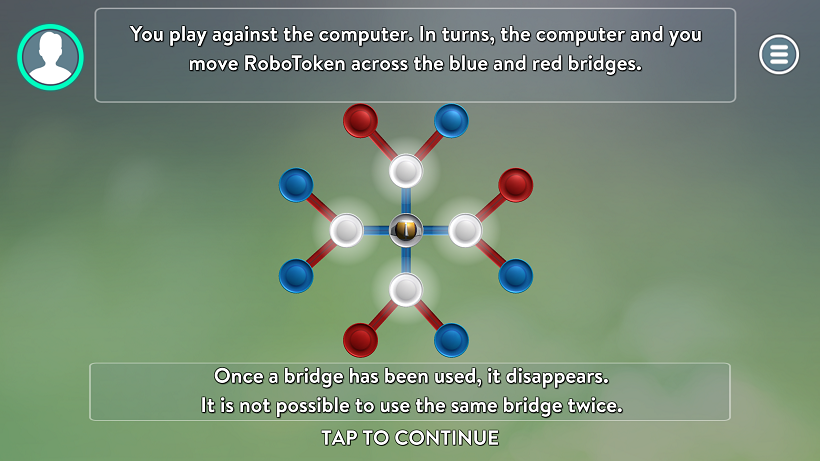

Supplement: S2 File — (ZIP) [file pone.0266366.s003.zip › T11s.png]

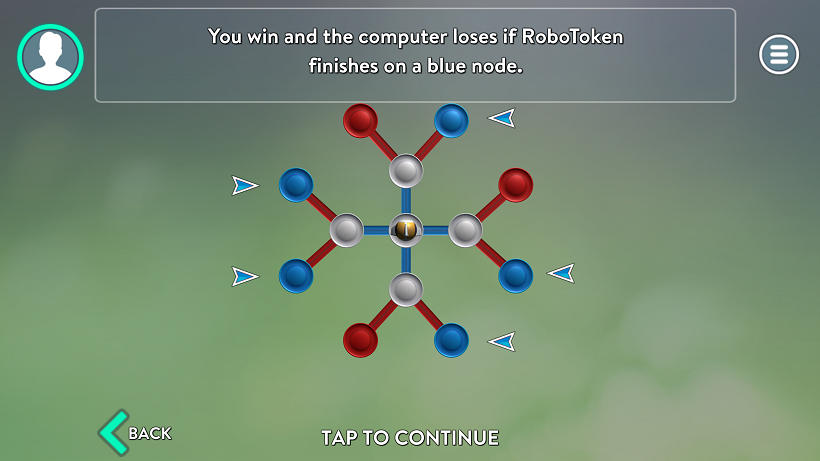

Supplement: S2 File — (ZIP) [file pone.0266366.s003.zip › T12s.png]

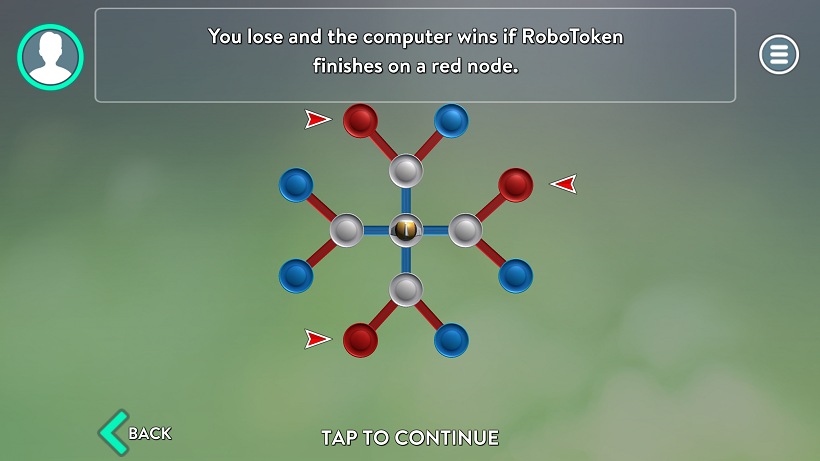

Supplement: S2 File — (ZIP) [file pone.0266366.s003.zip › T13s.png]

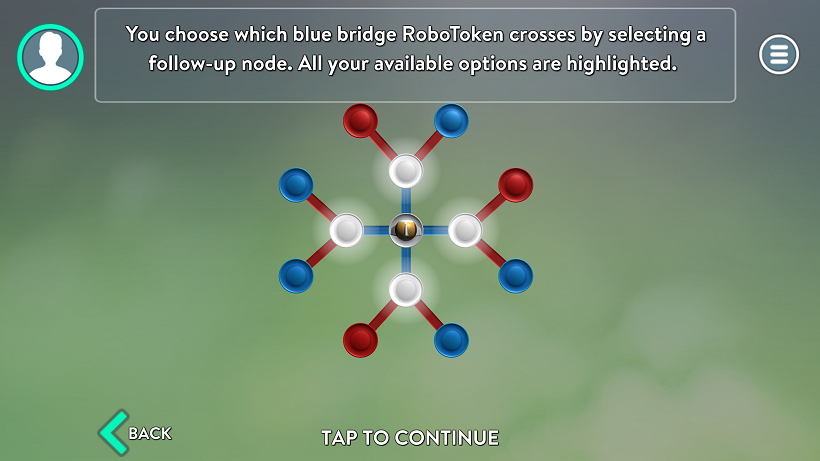

Supplement: S2 File — (ZIP) [file pone.0266366.s003.zip › T14s.png]

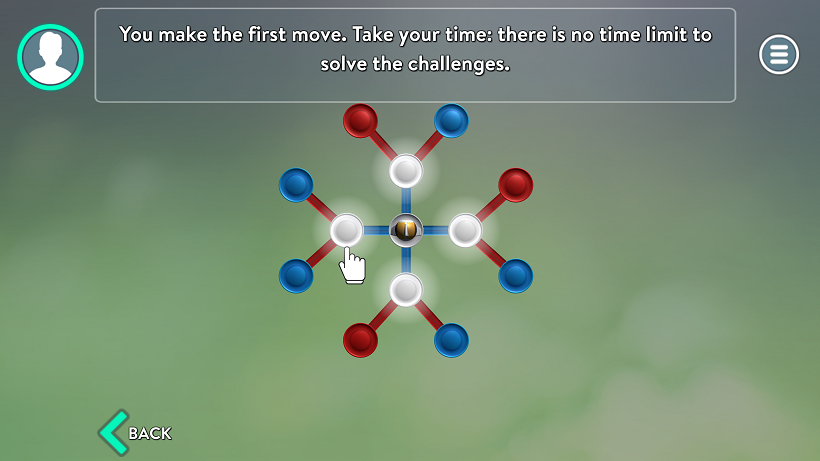

Supplement: S2 File — (ZIP) [file pone.0266366.s003.zip › T15s.png]

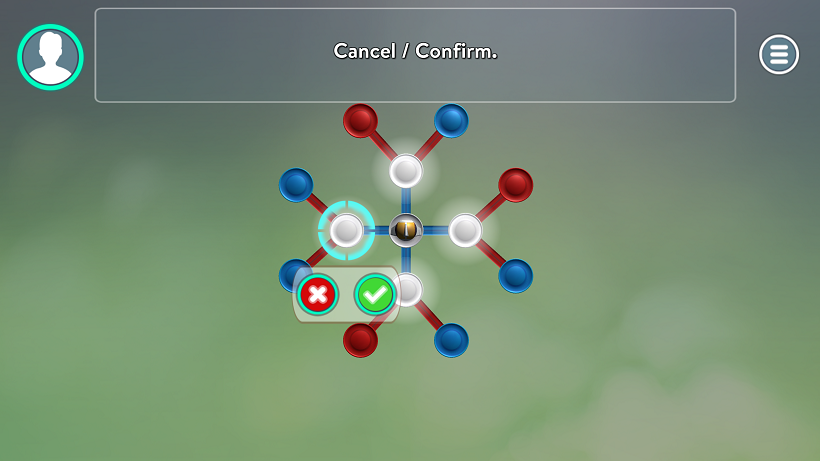

Supplement: S2 File — (ZIP) [file pone.0266366.s003.zip › T16s.png]

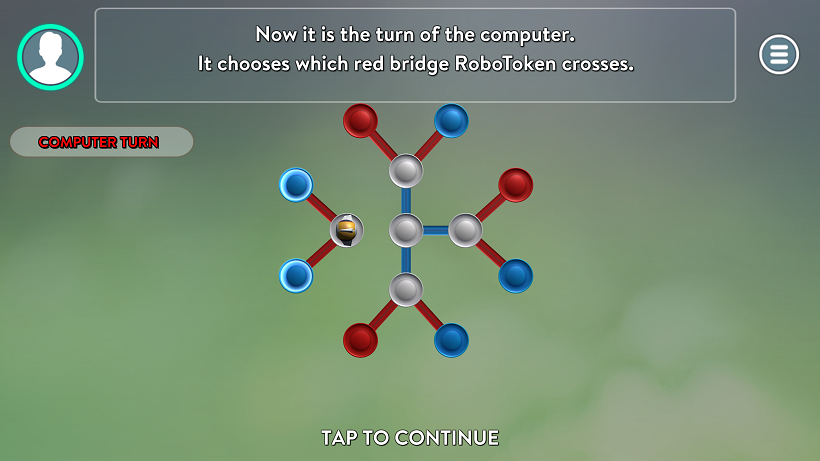

Supplement: S2 File — (ZIP) [file pone.0266366.s003.zip › T17s.png]

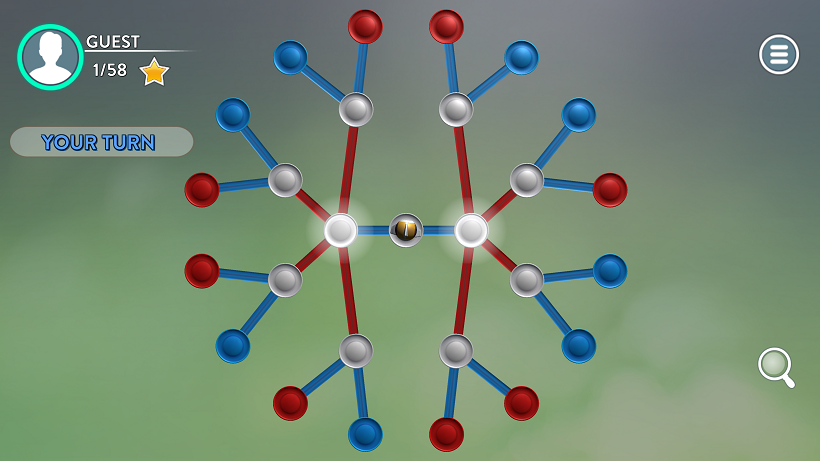

Supplement: S2 File — (ZIP) [file pone.0266366.s003.zip › T21s.png]
