# Supplementary material for: Profiling dynamic decision-makers
Source: PLoS One. 2022 Apr 14;17(4):e0266366. doi: 10.1371/journal.pone.0266366 (PMC9009624; doi:10.1371/journal.pone.0266366)
Supplement: S1 File — (PDF) [file pone.0266366.s002.pdf]

Dear Editor,

**Funding and IRB review.** The project has been founded by the University of Miami where both of us were faculty members. Currently, one of the authors (Grabiszewski) is not employed at the University of Miami while the other (Horenstein) is. Since our study is experimental, we requested to review our experiment by the IRB at the University of Miami. We received the following response:

> *The IRB determined that the proposed activity is not research involving human subjects as defined by DHHS and FDA regulations.*
>
> *IRB review and approval by this organization is not required. This determination applies only to the activities described in the IRB submission and does not apply should any changes be made. If changes are made and there are questions about whether these activities are research involving human in which the organization is engaged, please submit a study modification to the IRB for a determination.*

**Data.** We will provide data and code files as supplementary materials if the paper is published. If needed during the revision process, please let us know and we will provide everything in advance.

We confirm that none of the material has been published or is under consideration for publication elsewhere.

Thank you for your consideration.

Sincerely,

Alex Horenstein

Konrad Grabiszewski
